# Ecological corridors for the amphibians and reptiles in the Natura 2000 sites of Romania

**DOI:** 10.1038/s41598-020-76596-z

**Published:** 2020-11-10

**Authors:** Tiberiu C. Sahlean, Monica Papeș, Alexandru Strugariu, Iulian Gherghel

**Affiliations:** 1 Department of Patrimony Research, “Grigore Antipa” National Museum of Natural History, 011341 Bucharest, Romania; 2 Department of Ecology and Evolutionary Biology, University of Tennessee, Knoxville, TN 37996 USA; 3 Interdisciplinary Research Institute, “Alexandru Ioan Cuza” University of Iasi, 700505 Iasi, Romania; 4 Department of Biology, Case Western Reserve University, Cleveland, OH 44106 USA; 5 Faculty of Geography and Geology, “Alexandru Ioan Cuza” University of Iasi, 700505 Iasi, Romania

**Keywords:** Conservation biology, Ecological networks

## Abstract

Landscape heterogeneity and fragmentation are key challenges for biodiversity conservation. As Earth’s landscape is increasingly dominated by anthropogenic land use, it is clear that broad-scale systems of nature reserves connected by corridors are needed to enable the dispersal of flora and fauna. The European Union currently supports a continent-wide network of protected areas, the Natura 2000 program, but this program lacks the necessary connectivity component. To examine whether a comprehensive network could be built in order to protect amphibians and reptiles, two taxonomic groups sensitive to environmental changes due to their physiological constrains and low dispersal capacity, we used species’ distribution maps, the sites of community interest (SCIs) in Romania, and landscape resistance rasters. Except *Vipera ursinii rakosiensis*, all amphibians and reptiles had corridors mapped that, when assembled, provided linkages for up to 27 species. Natura 2000 species were not good candidates for umbrella species as these linkages covered only 17% of the corridors for all species. Important Areas for Connectivity were identified in the Carpathian Mountains and along the Danube River, further confirming these regions as hot spots for biodiversity in Europe, where successful linkages are most likely. In the end, while such corridors may not be created just for amphibians and reptiles, they can easily be incorporated into more complex linkages with corridors for more charismatic species, therefore enhancing the corridors’ value in terms of quality and structure.

## Introduction

Earth is experiencing widespread changes to natural environments: during the last century land use trends have changed and intensified, leading to a decrease in landscape heterogeneity and an increase in fragmentation^1–4^. These processes have been recognized as key challenges for the conservation of biological diversity across the world^5,6^, and have been identified as more important threats than climate change at regional and local scales^7,8^. The ecological consequences of habitat fragmentation have been reviewed extensively and are known to lead to loss of species in the remaining fragments, alter the composition of faunal assemblages, and change the ecological processes^9–11^. Species that are most sensitive to habitat fragmentation are either large-bodied animals that require extensive areas, species high on the food chain (such as birds of prey or large carnivorous mammals) or species with specialized food or habitat requirements^12–14^. The degree to which animal populations in fragments are isolated from those in nearby habitats varies greatly with land use and the biology of the species^14^. The reduced ability of animals to move through the landscape limits their capacity to rescue declining populations, to recolonize habitats where extinction may have occurred, or to colonize newly suitable habitats^15–19^.

A challenge coupled with large scale loss of natural habitats is preserving biodiversity in areas dominated by anthropogenic land use, where natural environments occur mostly as remnant patches of various sizes, but upon which the conservation of flora and fauna ultimately depends^14^. The traditional approach to nature conservation has been to select areas based on a set of criteria and manage them as different types of reserves, either by giving high priority to nature conservation or by balancing the conservation efforts with other types of land use. Currently, conservation biologists view this approach as insufficient for long-term conservation and propose to extend nature conservation through management of whole landscapes^20–25^. Among the strategies for nature conservation, stepping stones or preferably continuous corridors of habitat were recommended to facilitate the movement of animals between isolates^26,27^ as a less fragmented landscape pattern enables genetic exchange and supports metapopulations with higher prospects for survival, based on elevated levels of physical and functional connectivity^28–30^. The presence of corridors can also lead to a “rescue effect”—slowing down the rate of extinction through immigration to declining populations^31^. Corridors can be effective tools for conserving species that are habitat specialists or those that have a limited range of movement^14^.

Corridors can be defined as linear patches of habitat that are embedded in other types of land uses and are connecting two or more large blocks of habitat, usually referred to as “core areas”; corridors will maintain or enhance the viability of the populations in the core areas^30,32^. The main functions of a corridor are to provide habitat for certain, focal species, to allow movement of focal species, and to act as a barrier against unfavorable conditions^33^. Evidence in support of the utility of corridors as a conservation tool exist in literature^30,34,35^, revealing that features consistently direct the movement of diverse taxa^17^. Patch linkages are also an important response to climate change, as these can assist organisms in tracking suitable climatic conditions and extending their geographic range, maximizing the persistence of a species in their present geographic range^2,36,37^. Most importantly, linkages can counteract climate change by connecting protected areas to maximize the resilience of existing conservation networks^14,38^.

A system composed of nature reserves or protected areas and corridors enabling dispersal of flora and fauna, embedded within a matrix of diverse land uses represents an ecological network^39^, a concept which has evolved from the theory of island biogeography and metapopulations theory^40–42^. The European Union (EU) primarily relies on the Natura 2000 network for nature conservation, a system that covers approximately 18% of EU’s land surface^43–45^. The network comprises sites designated under the Birds (79/409/EEC; Special Protection Areas (SPAs)) and Habitats Directives (92/43/EEC; Special Areas of Conservation (SACs) & Sites of Community Interest (SCIs)), with the goal of ensuring long-term sustainability of habitats and species^46,47^, but until recently, the Natura 2000 scheme had not shown a significant level of ecological coherence^39^. In Romania, the Natura 2000 network lists 435 SCIs and 171 SPAs, covering ~ 22.8% (54 360 km^2^) of the land surface of the country. In 2013 the EU launched its Green Infrastructure (GI) Strategy, defined as “a strategically planned network of natural and semi-natural areas with other environmental features designed and managed to deliver a wide range of ecosystem services”^48^. The follow-up technical document on GI does not mention ecological corridors specifically, connectivity seemingly hoped to be provided by protected areas, extensive natural features, and low-intensity agricultural landscapes while also integrating existing green infrastructure in member countries^49^.

Amphibians and reptiles are sensitive to environmental changes, especially to climate change^50^, but also to fragmentation and degradation of their habitats as a result of human activities^8,51–53^; both taxonomic groups have low dispersal capacity^54–56^ and are exposed to predation, desiccation, and artificial barriers during migration or dispersal^8,57,58^, and habitat fragmentation is eroding their genetic diversity^56^. As a result of their intrinsic internal limitations, coupled with the fact that these two taxonomic groups are among the most understudied^59–61^, with little information available regarding their distribution, life history, ecological significance and abundance, it is easy to understand why they are generally not factored in when designing any types of corridors. But the reality is that amphibians and reptiles fit perfectly within the definition of focal species^62^, as most species are ecologically important^63^, area-sensitive^64^, dispersal limited, and sensitive to barriers^65^, thus making them ideal candidates for habitat connectivity modeling^65^. The majority of corridor designs tend to use mammalian models, and in many cases large carnivores are the prime targets, because they are charismatic^66^, well-studied^67^ and are more likely to receive funding^68^, sometimes even being proposed as umbrella species for whole communities^68^. However, large carnivores are habitat generalists, and can move through degraded habitats^69^ thus, from a corridor point of view, they are *passage species*, so only a limited number of their life-cycle requirements have to be met inside corridors^70^; amphibians and reptiles on the other hand make up the *corridor dwellers*, therefore all their life-cycle requirements have to be met inside the corridor, as they can take many generations to move through^70^. As a plus, amphibians and reptiles select habitat structure over size^71^, therefore the presence of these dwellers insures a more natural corridor.

The goal of our work was to examine whether the Romanian sites within the Natura 2000 network, responsible for the protection of amphibian and reptile species (i.e. SCIs), could be connected with habitat corridors (within a timeframe of 50 years), thus integrating Natura 2000 sites within an ecological network. First, we created corridors for each species to connect all SCIs (used here as core areas) where a taxon was deemed present, and we examined the degree of connectivity. Here we included a novel method of scoring resistances that is less opinion-based because it relies more on the statistics obtained from the actual distribution range of the species, therefore reflecting the range of environmental conditions that are presently tolerated by each species. Next, we examined the viability of multi-species corridors, which would further increase their value: as species included in the Annex II of the Habitats Directive are prioritized for conservation, we explored the idea of using such taxa as umbrella species. Finally, by pooling the least-cost paths (LCPs) that resulted from the corridor modeling phase we were able to highlight areas that are important for the connectivity of amphibian and reptile species, but which are not protected, as they are currently outside the existing network of protected areas.

## Results

### Single species corridors

Corridor maps for each species are available in the Supplementary Material S2—Maps (Figures S1–S41). The length of the mapped corridors ranged from 200 m to 114 km for amphibians, and from 200 m to 89 km for reptiles. Mean corridor length was ~ 7.6 km for amphibians and ~ 8.8 km for reptiles. Among amphibian species, *Bufo bufo* had the longest mapped continuous habitat corridor at 114 km, while the reptile with the longest link (89 km) was *Natrix tessellata*. Connectivity ranged from 33 to 80% for amphibians and from 0 to 100% for reptiles (Table 1). In the case of amphibians, *Triturus dobrogicus* had the lowest connectivity (33%), followed by *Bombina bombina* (36%), while the sites for *Ichthyosaura alpestris* were best connected by habitat corridors (80%). Among reptiles, vipers’ sites were both the best and least connected: *Vipera ammodytes ammodytes* obtained a perfect connectivity between sites (100%), while *V. ursinii moldavica* and *V. nikolskii* sites had among the lowest connectivity of all reptiles (22% and 18%); no sites were connected through habitat corridors for the other meadow viper (*V. u. rakosiensis*).

**Table 1:** Connectivity of Natura 2000 sites for each species of amphibian and reptile.

Species	No. of sites	No. of sites connected (% connected)
**Amphibians**		
*Salamandra salamandra*	144	92 (64)
*Lissotriton vulgaris vulgaris*	217	103 (48)
*Lissotriton vulgaris ampelensis*^a^	43	20 (47)
*Lissotriton montandoni*^a^	56	42 (75)
*Ichthyosaura alpestris*	102	82 (80)
*Triturus cristatus*^a^	185	82 (44)
*Triturus dobrogicus*^a^	33	11 (33)
*Bombina bombina*^a^	162	59 (36)
*Bombina variegata*^a^	208	118 (57)
*Bufo bufo*	212	136 (64)
*Bufo viridis*	222	112 (51)
*Pelobates fuscus*	105	42 (40)
*Pelobates syriacus balcanicus*	32	12 (38)
*Rana dalmatina*	207	100 (48)
*Rana temporaria*	176	122 (69)
*Rana arvalis*	54	26 (48)
*Hyla arborea*	211	137 (65)
**Reptiles**		
*Emys orbicularis*^a^	141	68 (48)
*Testudo graeca*^a^	27	13 (48)
*Testudo hermanni*^a^	10	6 (60)
*Ablepharus kitaibelii*	27	12 (44)
*Eremias arguta*	5	3 (60)
*Darevskia praticola*	23	12 (52)
*Lacerta agilis*	244	123 (50)
*Lacerta viridis*	189	84 (44)
*Lacerta trilineata*	17	10 (59)
*Podarcis muralis*	92	66 (72)
*Podarcis tauricus*	53	24 (45)
*Zootoca vivipara*	94	81 (86)
*Anguis fragilis*	156	86 (55)
*Natrix tessellata*	118	77 (65)
*Eryx jaculus*	6	2 (33)
*Coronella austriaca*	129	74 (57)
*Dolichophis caspius*	42	19 (45)
*Elaphe sauromates*^a^	13	3 (23)
*Zamenis longissimus*	99	61 (62)
*Vipera berus*	126	95 (75)
*Vipera nikolskii*	11	2 (18)
*Vipera ammodytes ammodytes*	25	25 (100)
*Vipera ammodytes montandoni*	11	7 (63.6)
*Vipera ursinii rakosiensis*^a^	4	0 (0)
*Vipera ursinii moldavica*^a^	9	2 (22)
**Multi-species**		
All herptiles	342	211 (62)
All amphibians	323	206 (64)
All reptiles	312	168 (54)

^a^Species listed in Annex II of the Habitats Directive.

### Multispecies corridors

**Figure 1:**
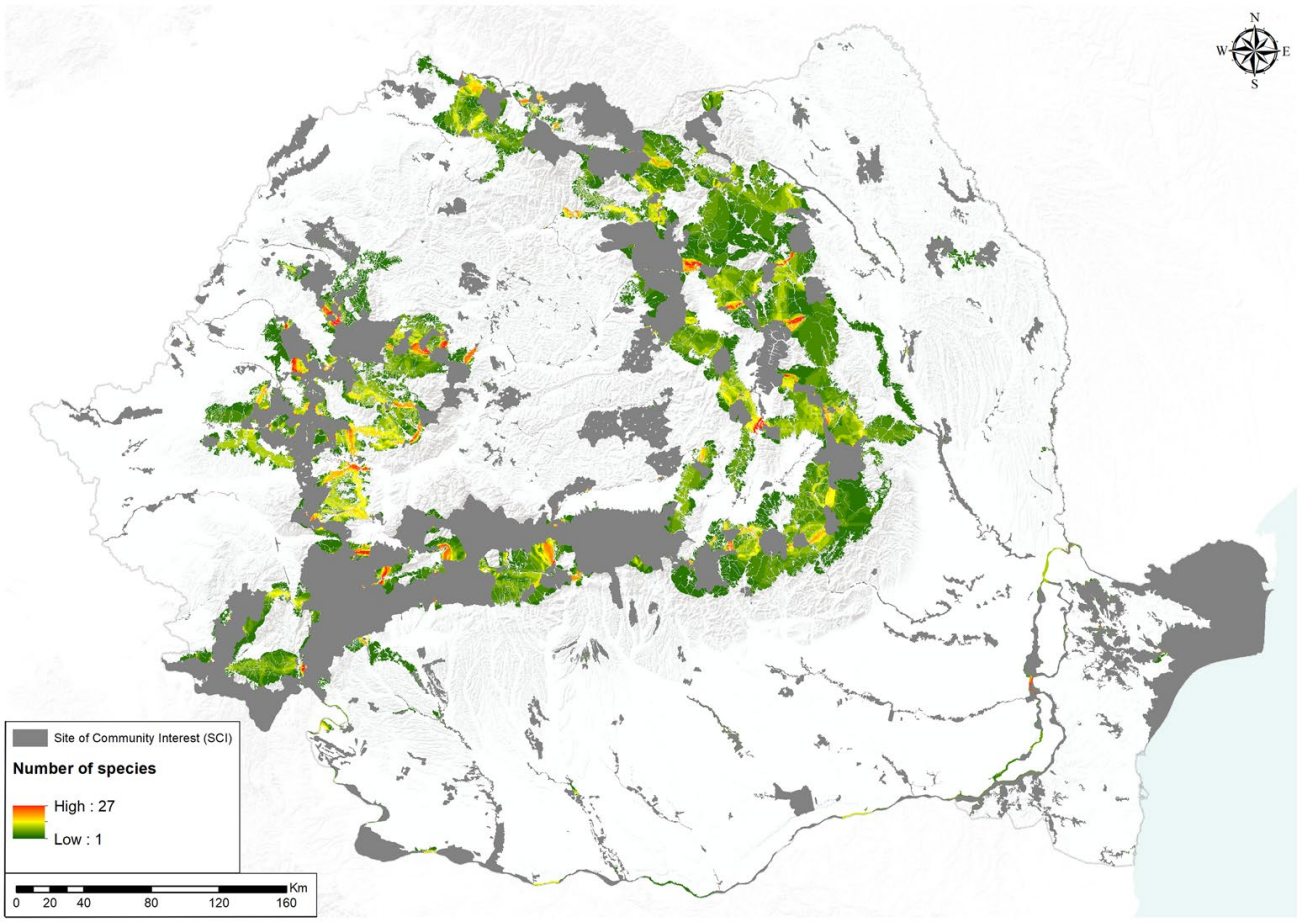
Multi-species corridors obtained by merging individual corridors for each species (map generated using ArcGIS 10.4^72^).

Assembled, the corridors provided linkages for up to 27 species if all herpetofauna was taken into account (Fig. 1), 16 species when reptiles were considered separately (Supplementary Material S2—Maps, Figure S44) and 13 taxa for amphibians (Supplementary Material S2—Maps, Figure S43); 63% of the area covered by the corridors was shared by more than one species if all amphibians and reptiles were combined, 46% when amphibians were considered separately, and 53% for only reptiles. When taking into account only species from Annex II of the Habitats Directive, the maximum number of species in the habitat corridors was 6 for all amphibians and reptiles (Supplementary Material S2—Maps, Figure S45), 5 when assembled only for amphibians and 3 if assembled only for reptiles. The overlap between the corridors involving only Natura 2000 species and corridors where all species were included was 17%; the overlap between the corridors for the amphibians from Annex II and the corridors for all amphibians was 8%, and when analyzing reptiles, the overlap was ~ 20%. Connectivity between Natura 2000 sites was about 60% when links were considered for all herpetofauna species (Table 1) or only for amphibians, and 54% when connectivity was explored only for reptiles.

### SCI connectivity

**Figure 2:**
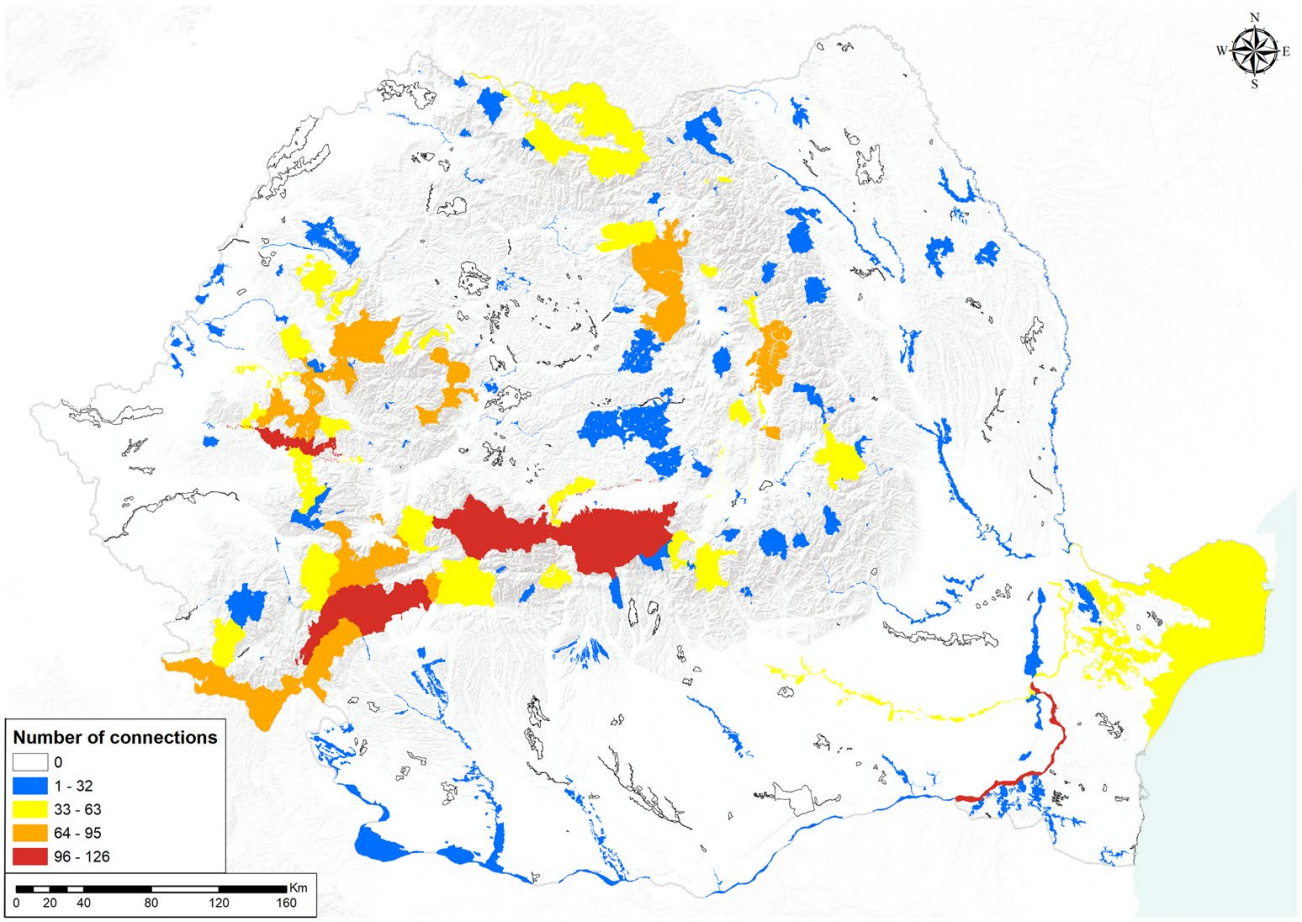
Connectivity of Natura 2000 SCIs based on the number of incoming and outgoing least cost paths (LCPs) for amphibians and reptiles (map generated using ArcGIS 10.4^72^).

When data from all herpetofauna taxa were pooled together, the number of connections for the protected areas ranged from 0 (no connectivity) to 126 connections (Fig. 2). SCIs that lacked any kind of connectivity for amphibians or reptiles were generally located outside the range of Carpathian Mountains, inside the Transylvanian Plateau and in southern Dobrogea, while the core areas with the greatest number of connections were located in the Carpathian Mountains and along the lower Danube River. A similar pattern was obtained when each group was treated individually, with isolated core areas located outside Carpathian Mountains, but the number of connections per core ranged from 0 to 66 for amphibians and from 0 to 62 for reptiles (Supplementary Material S2—Maps, Figures S47–S48).

### Important areas for connectivity (IAC)

**Figure 3:**
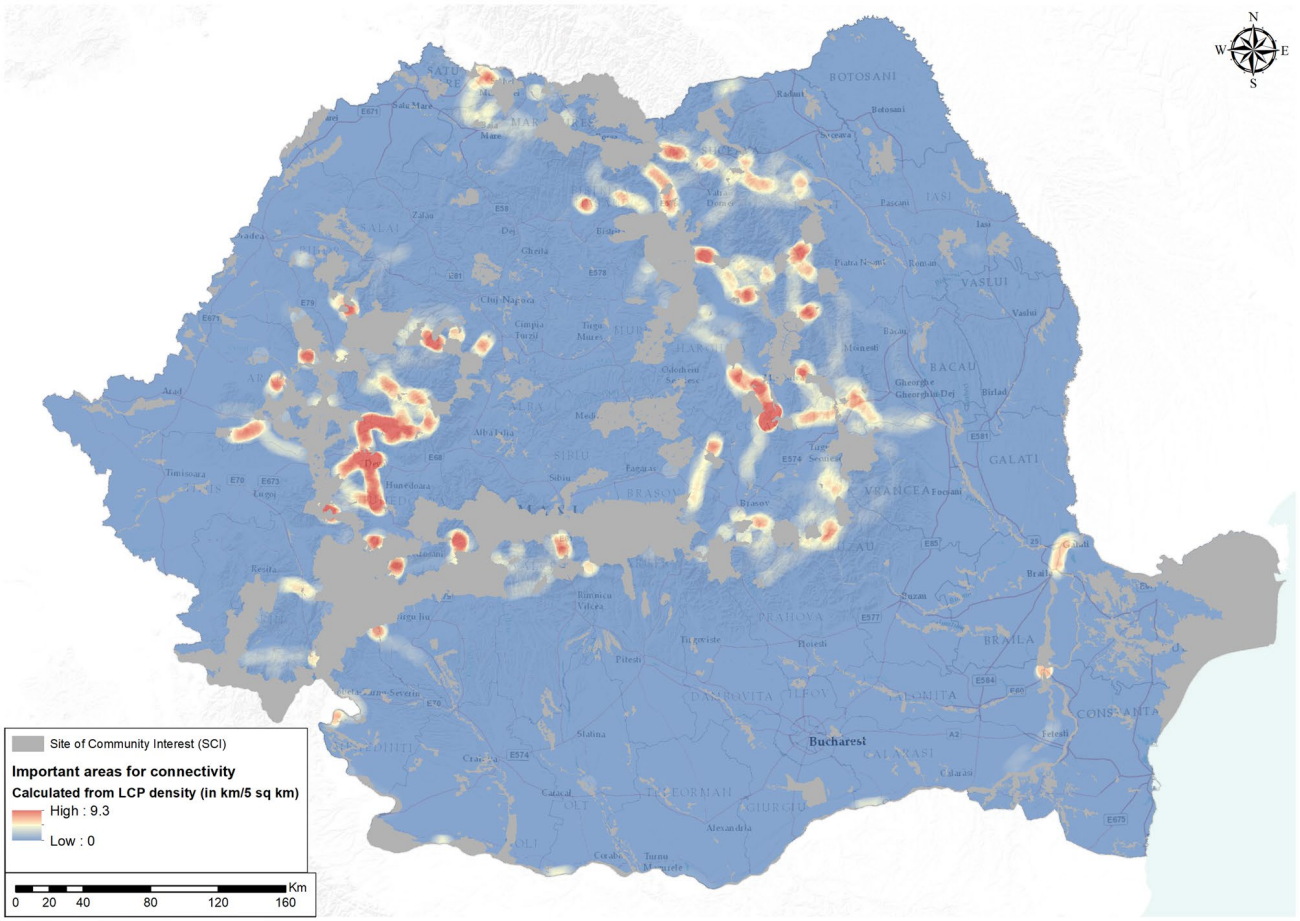
Important Areas for Connectivity based on least cost path (LCP) density (in km/km^2^) (map generated using ArcGIS 10.4^72^).

When pooled together for all amphibians and reptiles, the LCPs showed important areas for connectivity mainly in the Carpathian Mountains and sporadically along the Danube River (Fig. 3). In the Carpathians, most of the important areas for connectivity were identified in the Western Carpathian Range (and surrounding hills) and in the Eastern Carpathian Range, and some patches in Lotrului Mountains, in Retezat-Godeanu Massif and in Șureanu Mountains from the Southern Carpathians. Along the Danube River, the important areas for connectivity were located downstream from the city of Drobeta Turnu Severin, near Balta Mică a Brăilei Natural Parc and between Brăila and Galați.

## Discussion

Our study explored whether habitat corridors could be a viable option for connecting the existing network of Romanian Natura 2000 sites for the amphibian and reptile species that occupy the sites. During the study design we elected to use only the fragments of the landscape that can be considered “natural” (such as forests, shrubs, rivers etc.) although animals, even amphibians and reptiles, are known to move through semi-natural or even heavily modified landscapes^73–77^. We had multiple reasons in choosing this approach. Foremost, natural habitats have exceptional value for both connectivity and biodiversity^78,79^—they maximize animal mobility, contain higher taxonomic diversity, higher genetic diversity, and meta-population retention, thus represent refugia for biodiversity. In contrast, areas impacted by human activities are viewed as unlikely to hold sufficient connectivity value for many key elements of biodiversity^78^. Human-modified landscapes are also susceptible to frequent land use changes, one of the primary sources of decline for both amphibians and reptiles^55,80,81^. It is generally agreed that avoiding degradation of existing habitat is a better strategy than restoration, as the latter implies increased costs and is unlikely to lead to full recovery^78,82^; corridors generally imply substantial financial efforts and, as a result, their implementation has been met with resistance throughout Europe^83^. Human-modified landscapes could be included within the network in a stepping-stone manner^84^, but this would require a case-by-case analysis and consultations with the stakeholders, both of which are beyond the scope of this manuscript.

### Natura 2000 connectivity and species migration corridors

Our results showed that, at least for some SCIs and species, connectivity can be achieved using only natural patches of habitat; for other species, semi-natural areas will have to be taken into account, or landscape restoration will be necessary to promote connectivity. The taxa most affected by poor connectivity seem to be those with very specific habitat requirements, such as species at the edge of their distribution range or endemics (e.g. *Eryx jaculus, Elaphe sauromates, V. u. moldavica, V. u. rakosiensis*). Species occupying sites located at low elevations (e.g. *Pelobates syriacus, Eremias arguta*) also displayed poor connectivity, as those regions of the Romanian landscape have been affected by intense agricultural activities^68^. Some of the linkages mapped are accessible through a one-time dispersal event, while others would require multiple generations during which the species become corridor dwellers, but in fact this increases the overall occupancy and metapopulation connectivity of the landscape matrix^85^. The main issue is that, because of the high cost associated with allocating land for corridors, linkages for single species are an unrealistic undertaking, so possible alternatives are to (a) either find corridors common for as many species as possible, (b) use umbrella species to cover the needs of other species/groups or (c) merge the mapped corridors for amphibians and reptiles within existing/future planned linkages for other taxa.

By examining the multi-species corridors, we observed that the Carpathian Mountains are the most likely landscape unit where these linkages can be created with great success. The Carpathians are a well-known hot spot for biodiversity in Europe^86^, with large continuous natural areas^87^, and projects involving connectivity in the Carpathians have been developed in recent years^88^. Areas along the Danube River also showed great connectivity, where almost continuous corridors between SCIs were obtained (more so for amphibians than reptiles). These results are supporting the ongoing project to create the Lower Danube Green Corridor, which aims to enforce existing protected areas, create new ones, as well as restore natural floodplains^89^. Moreover, the corridors further emphasize the importance of riparian habitats as natural linkages^90^. Sites of community interest located within the Carpathian Mountains or along the lower Danube River were also likely to exhibit the highest degree of connectivity, as evidenced by our results, while many sites within intense anthropogenic settings lacked any kind of connectivity and will likely require habitat restoration measures or land use regulation policies to facilitate future corridors.

Overall, more than half of the species of amphibians and reptiles analyzed were grouped in shared corridors (more than one species) when linkages were pooled together and core connectivity was above average, making the respective swathes of land more valuable for nature conservation, especially taking into account that those are exclusively natural fragments and so would necessitate only minor investments.

### Conservation implications

Because the species listed in Annex II of the Habitats Directive are given higher priority (with more funds being allocated for their conservation) compared to taxa from other annexes, we explored the possibility of using them as “umbrella species” (sensu Breckheimer et al.^91^) but the results were not very encouraging, as the corridors for the Natura 2000 species of herpetofauna covered only a small fraction of the corridors for all species. This can be explained by the fact that, with few exceptions, the species from the Annex II generally have specific habitat requirements and are themselves declining. Our method was to simulate corridors for each species of amphibian and reptile and combine them into general habitat corridors, but this will not be possible in other situations, such as working with tight deadlines, fixed budgets, an array of species from different groups, and much greater species diversity or little knowledge of the organisms for which connectivity is to be provided. Also, the topic of umbrella species remains intensely debated^92–94^ with no right or wrong answer, mostly because species (even from the same group) can differ greatly in resource utilization, habitat requirements, and dispersal capability^92^. One convenient method would be to use a small number of wide-dispersing and habitat generalist species^91,95^ as umbrella species in the context of connectivity, but some results have shown that, if less than three species are used, an indirect approach to connectivity using habitat characteristics is more effective^92^. A more viable method is to use a number of 5^96^ to 9^92^ surrogate species; however, our results show that even in this case the species should be chosen carefully, by applying dimensionality reduction or grouping^97^. We used 12 species from the Annex II of the Habitats Directive as surrogates for the Romanian herpetofauna, but the corridors only fitted the needs of a small number of species and the overlap with the multi-species corridor was reduced; the same was true even if the corridors were pooled based on taxonomic groups.

By pooling the corridors for all species we noticed that “intersections” would form where many corridors merged, these areas being located outside the boundaries of designated SCIs. As such, using the LCPs we isolated these “hot-spots” which we termed “Important Areas for Connectivity”; these are landscape features where the density of LCPs is the greatest and therefore the highest number of corridors pass through those specific locations. These features could be proposed as SCIs in the future, therefore creating connectivity in a “stepping-stone” manner^14^, or they could be given enhanced priority because of their importance in promoting linkages. Future research should compare these areas to those obtained for other species or taxonomic groups, with the goal of finding common ground that would further consolidate the corridor status of these areas and make them more likely to be adopted by national authorities.

### Concluding remarks

Amphibians and reptiles are not the most popular model organisms for conservation^55,60^ and studies on corridors generally take into account mammalian and avian taxa, with so-called “flagship species” (generally large carnivores)^98^ being at the forefront. However, through their intrinsic characteristics, amphibians and reptiles make very good model organisms for connectivity analyses^65,70^. The landscape linkages obtained in this study raise the alarm on the fact that the complex network of sites created through the Natura 2000 program does not function like a system; there are sites and species that cannot be connected through areas of natural habitat, so these sites are of utmost importance and should be prioritized for conservation^78^. Some of the species that had low connectivity or no connectivity (e.g. *Elaphe sauromates*, *V. u. moldavica, V. u. rakosiensis*) are precisely those prioritized for conservation through the Habitats Directive. Moreover, these linkages can be used when mapping complex corridors (covering a range of plants and animals) or when corridors focused on large carnivores (the brown bear, the grey wolf and the Eurasian lynx) are created, as amphibian and reptile species value habitat structure over size^71^, therefore enhancing their quality and value and resulting in more species inhabiting the linkages^14,99^.

Last but not least, the IACs identified in our study show that there are areas of utmost importance to the functionality of amphibian and reptile communities (i.e. providing connectivity) that are outside any type of protection; these areas are composed entirely of natural blocks of land that, in the context of today’s anthropogenic intervention, may soon disappear. These IACs could also be vital for amphibians and reptiles for adaptation under anthropogenic climate change, as they would allow populations to track their preferred microclimates^38,78^.

## Methods

### Study species

The Romanian herpetofauna consists of 46 taxa^100,101^, but our analysis included only 42 species. With the exception of the grass snake (*Natrix natrix*), all species are protected in some form by national and EU legislation, mainly by Law 49/2011 approving UGO 57/2007, which amends the Habitats Directive (Council Directive 92/43/EEC). Because our analysis focused solely on the Natura 2000 network and the species it protects through the Annexes of the Habitats Directive, the grass snake was omitted. We also excluded from our study the water frogs (*Pelophylax. ridibundus, P. lessonae* and *P.* kl. *esculentus*) because species identification is difficult in the field and, having no way of verifying past records, it could lead to erroneous records and, in turn, misplaced corridors^100^.

### Distribution data

We collated the baseline distribution for the herpetofauna of Romania from two peer-reviewed papers^100,101^. The distribution data presented in the two papers were not publicly available, thus we manually georeferenced the distribution maps in ArcGIS 10.4^72^. The resulting distribution grids were further revised with records collected by three of us (T. C. S., A.S., and I.G.) (see Supplementary Material S3—Additional methods information for more data).

### Core areas

We considered the Sites of Community Interest (SCIs) as core areas for mapping landscape linkages for amphibians and reptiles. Each site has a standard form where protected species are mentioned, but which is not always updated; we therefore chose to create an updated list for each site (see Supplementary Material S3—Additional methods information).

### Environmental variables and resistance layers

The resistance rasters used in the analysis were created through the traditional method of assigning scores to different variables based on experts’ opinion regarding the ability of animals to negotiate certain environmental features^102–105^, with the modification that we used information from the species’ current range (in the form of spatial statistics for each environmental variable) to reduce subjectivity. In ArcGIS, we extracted basic statistics (min, max, 1st percentile, 3rd percentile) of seven environmental variables (Table 2) from the species’ distribution grids and established five classes for scoring: (C1) raster minimum to species’ distribution minimum, (C2) minimum to 25%, (C3) 25–75%, (C4) 75% to species’ distribution maximum, and (C5) species’ distribution maximum to raster maximum. The goal was to score different values of an environmental variable depending on how readily available they are for the species, but only when also accounting for what is known about the species’ current range through the distribution grids (see Supplementary Material S3—Additional methods information). The score values ranged from one to five, where one represented areas readily available for the species, and five signaled areas difficult to navigate (Supplementary Material S1—Resistance rasters scoring). A value of 1000 was used to show barriers or features which we considered impossible to pass. Some intervals included values outside the species’ range in Romania but which are not outside the species’ environmental limits; as such these areas were judged based on the known biology of the species and scored accordingly. Moreover, values from the first or the last interval almost always received a high (5) or a low (1) score, or a value of 1000 if they were considered barriers, because they usually reflect conditions towards the limits of what the species is able to tolerate. The landcover variable was treated separately as it consisted of categorical data; the human-modified landcover classes were given a score value of 1000 because the goal was to connect core areas using existing natural patches of habitat, resulting in 13 landcover categories capable of sustaining habitat corridors (Supplementary Material S1—Resistance rasters scoring). We reclassified the variables based on scoring categories in ArcGIS 10.4. Two variables (DEM and slope) were aggregated from their original resolution of 25 m to 100 m to match the lowest resolution (100 m) of the other environmental variables. The final resistance rasters were mosaicked in ArcGIS prioritizing high values (mosaic operator MAXIMUM) and then resampled to a resolution of 200 m to reduce CPU time and avoid cost analysis errors caused by very large datasets.

**Table 2:** Variables used for creating resistance rasters.

Variable	Resolution (m)	Measurement unit	Source
Digital elevation model (DEM)	25	m	European Environmental Agency (www.eea.europa.eu)
Slope	25	°	Derived from DEM
Hydrographic Network density	100	km/km^2^	Based on data obtained from the European Environmental Agency (www.eea.europa.eu)
Road network density	100	km/km^2^	Based on data obtained from www.openstreetmap.org
Distance to water	100	m	Based on data obtained from the European Environmental Agency (www.eea.europa.eu)
Distance to road	100	m	Based on data obtained from www.openstreetmap.org
Corine Land Cover 2012	100	–	Data obtained from Copernicus Land Monitoring Services (https://land.copernicus.eu/)

### Corridor model

We used the Linkage Pathways Tool of the Linkage Mapper Toolbox for ArcGIS^106^ to create corridors for each of the 42 species of amphibians and reptiles included in the analysis. Similarly to other connectivity modeling tools, Linkage Mapper uses as inputs a set of core areas to be connected and a resistance layer. The result is a raster surface of cost-weighted distances normalized by least-cost paths (LCPs) between core areas. When working with large datasets, Linkage Mapper requires less processing time than alternative mapping software^90^. The toolbox allows the user to limit the maximum length of a corridor based on a cost-weighted or Euclidean distance or other user-defined criteria. First, we created a model without any limitations, but corridors were identified spanning very large distances (> 100 km) with unrealistic chances of connectivity. Therefore, on the second run we took into account a time-frame of 50 years as the maximum time span during which connectivity could be achieved, as data regarding the dispersal capacity of amphibians and reptiles in Romania were available from Popescu et al.^107^. Corridors were discarded if the LCPs intersected an intermediate core area; the number of neighboring core areas connected was not limited. Post-modeling, we clipped off corridors to a cutoff width equal to twice the size of the yearly dispersal range of species to include space for a main corridor and a buffer zone (see Supplementary Material S3—Additional methods information).

### Multispecies corridors

Post-mapping analysis explored the idea of multi-species corridors as linkages for individual species are difficult to justify and biological conservation is most effective when the strategies applied meet the needs of many species^108^. We reclassified the truncated corridors for each species to a value of one and we mosaicked linkages based on the following strategies: (1) corridors for all herpetofauna taxa; (2) corridors for all species of amphibians; (3) corridors for all species of reptiles; (4) corridors for the Annex II Natura 2000 species. Besides exploring the viability of multi-species corridors, we also analyzed the possibility of umbrella species, i.e. whether corridors for a limited number of species could cover the space necessary for a whole group.

### SCI connectivity

We also aimed to evaluate the contribution of the sites (SCIs) to the overall ecological network and we did this by counting the number of times each core area was successful in achieving a connection to another core area. We obtained the information from LCPs and we created connectivity counts for all taxa, and for amphibians and reptiles separately.

### Important areas for connectivity

After creating the multispecies corridors, it became clear that some areas are very important for the dispersal of several taxa and when overlapping with the SCI network, we realized that those features were generally located outside protected areas. Therefore, using the least cost paths (LCPs) and the line density function in ArcGIS, we mapped areas of high value for the connectivity of herpetofauna.

## Supplementary information

Supplementary information 1.

Supplementary information 2.

Supplementary information 3.

## Data Availability

The datasets generated during and/or analyzed during the current study are not publicly available due to very large size, but are available from the corresponding author on reasonable request. The rest of data generated or analyzed during this study are included in this published article and its supplementary information files.
