# Supplementary figures and images for: Ecological corridors for the amphibians and reptiles in the Natura 2000 sites of Romania
Source: Sci Rep. 2020 Nov 10;10:19464. doi: 10.1038/s41598-020-76596-z (PMC7655805; doi:10.1038/s41598-020-76596-z)

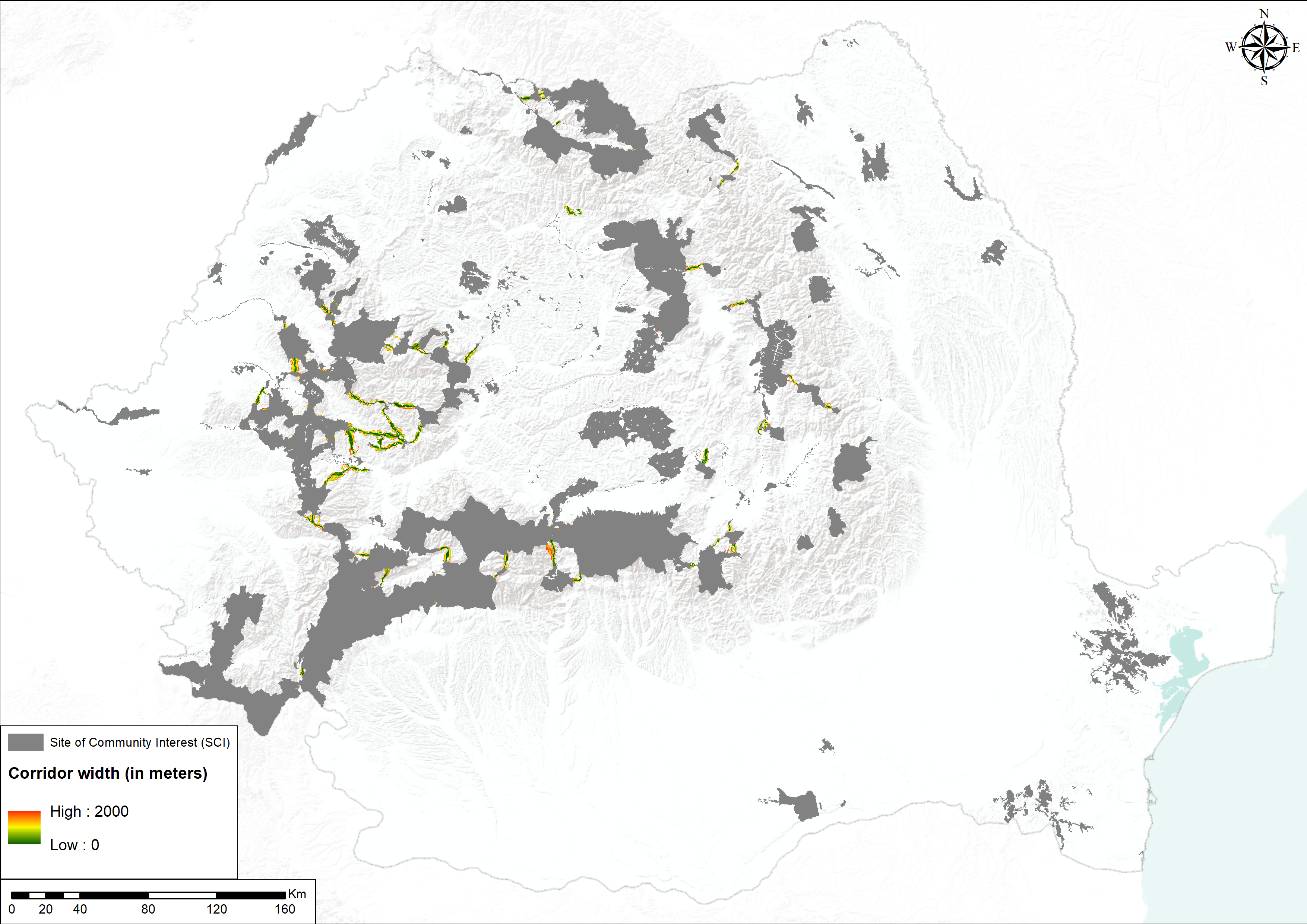

Supplement: Supplementary file 2 — Supplementary information 2. [file 41598_2020_76596_MOESM2_ESM.zip › Supplementary Material S2 Maps/Figure 1 Corridors for Anguis fragilis.png]

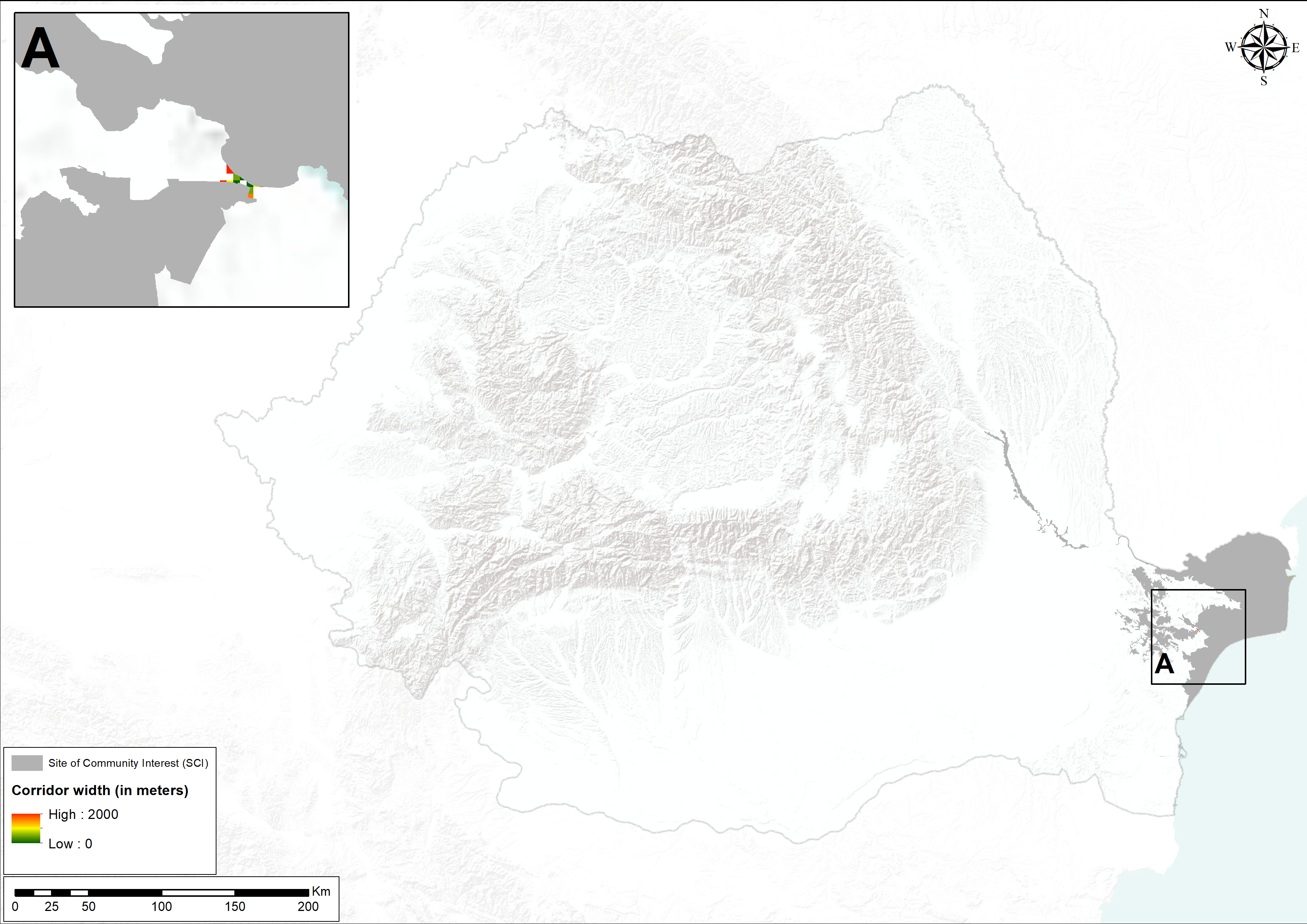

Supplement: Supplementary file 2 — Supplementary information 2. [file 41598_2020_76596_MOESM2_ESM.zip › Supplementary Material S2 Maps/Figure 10 Corridors for Eremias arguta.png]

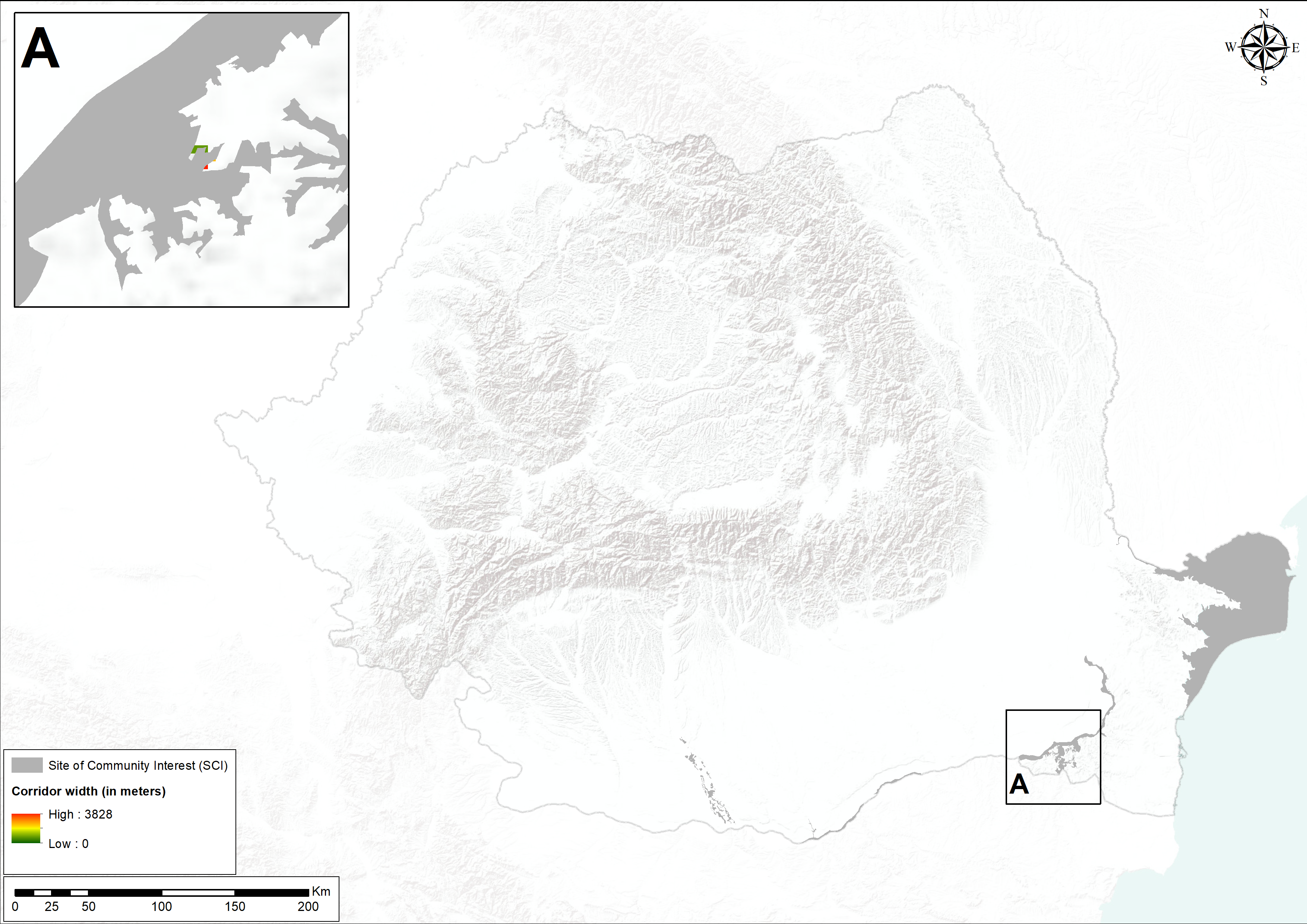

Supplement: Supplementary file 2 — Supplementary information 2. [file 41598_2020_76596_MOESM2_ESM.zip › Supplementary Material S2 Maps/Figure 11 Corridors for Eryx jaculus.png]

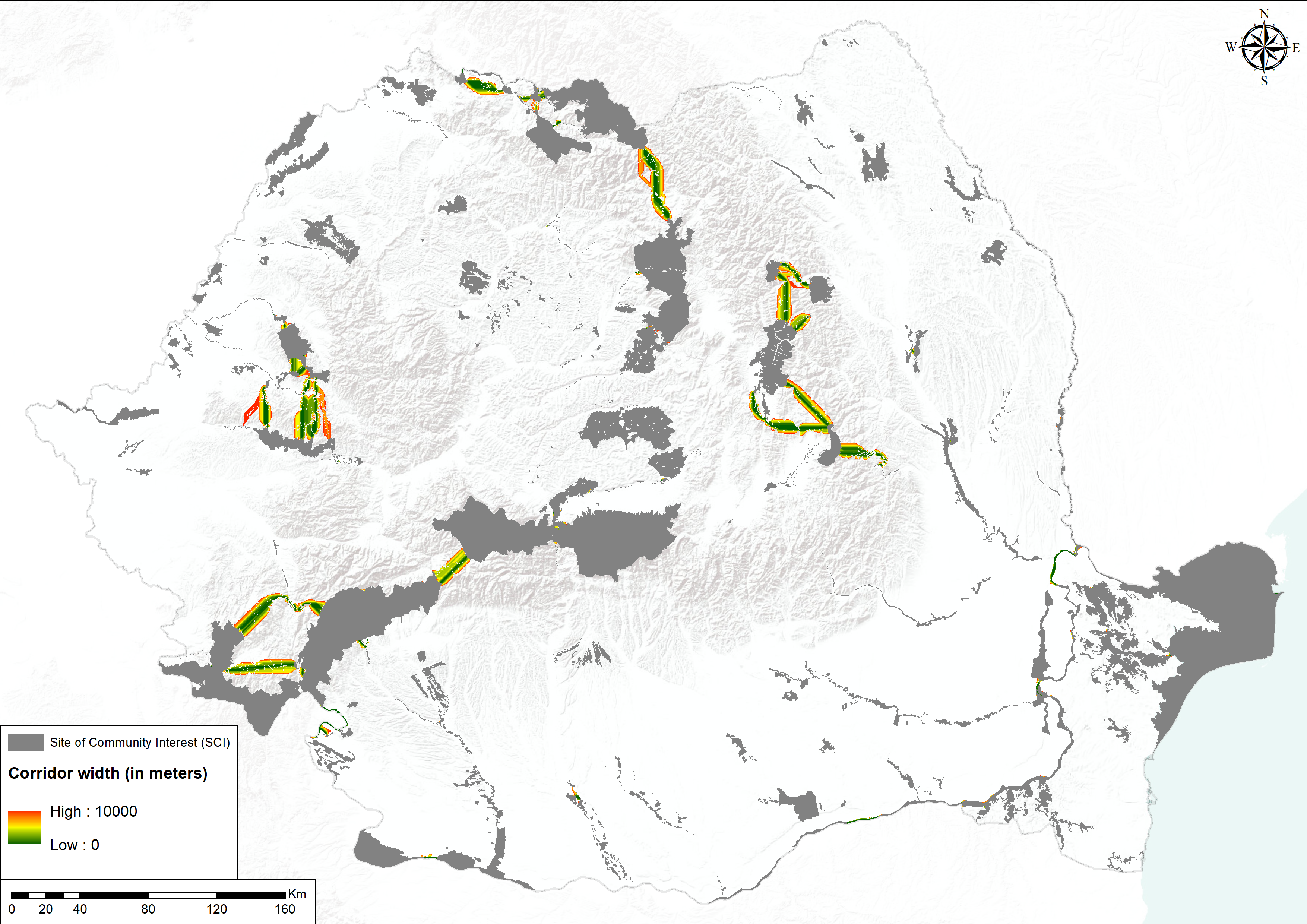

Supplement: Supplementary file 2 — Supplementary information 2. [file 41598_2020_76596_MOESM2_ESM.zip › Supplementary Material S2 Maps/Figure 12 Corridors for Emys orbicularis.png]

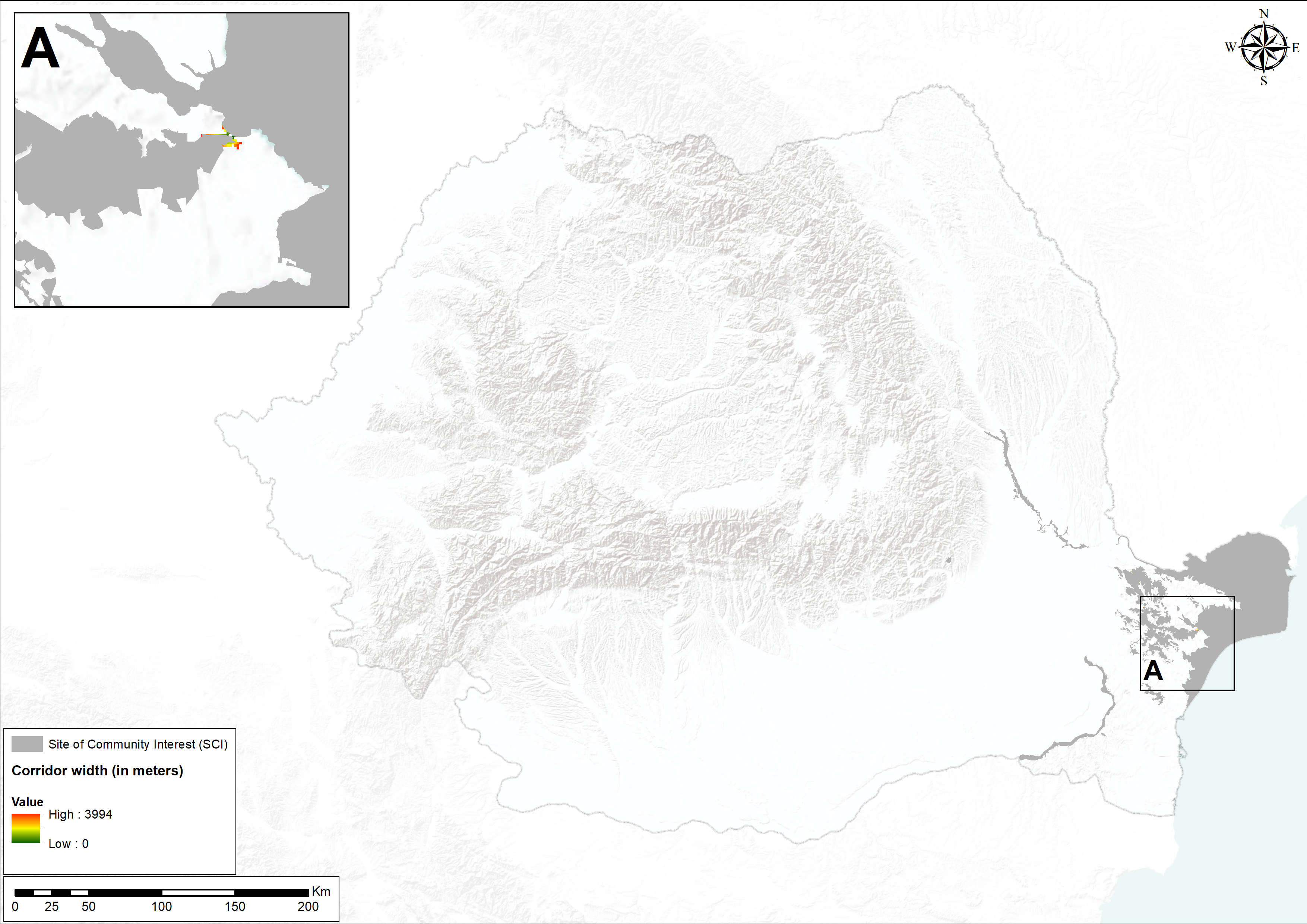

Supplement: Supplementary file 2 — Supplementary information 2. [file 41598_2020_76596_MOESM2_ESM.zip › Supplementary Material S2 Maps/Figure 13 Corridors for Elaphe sauromates.png]

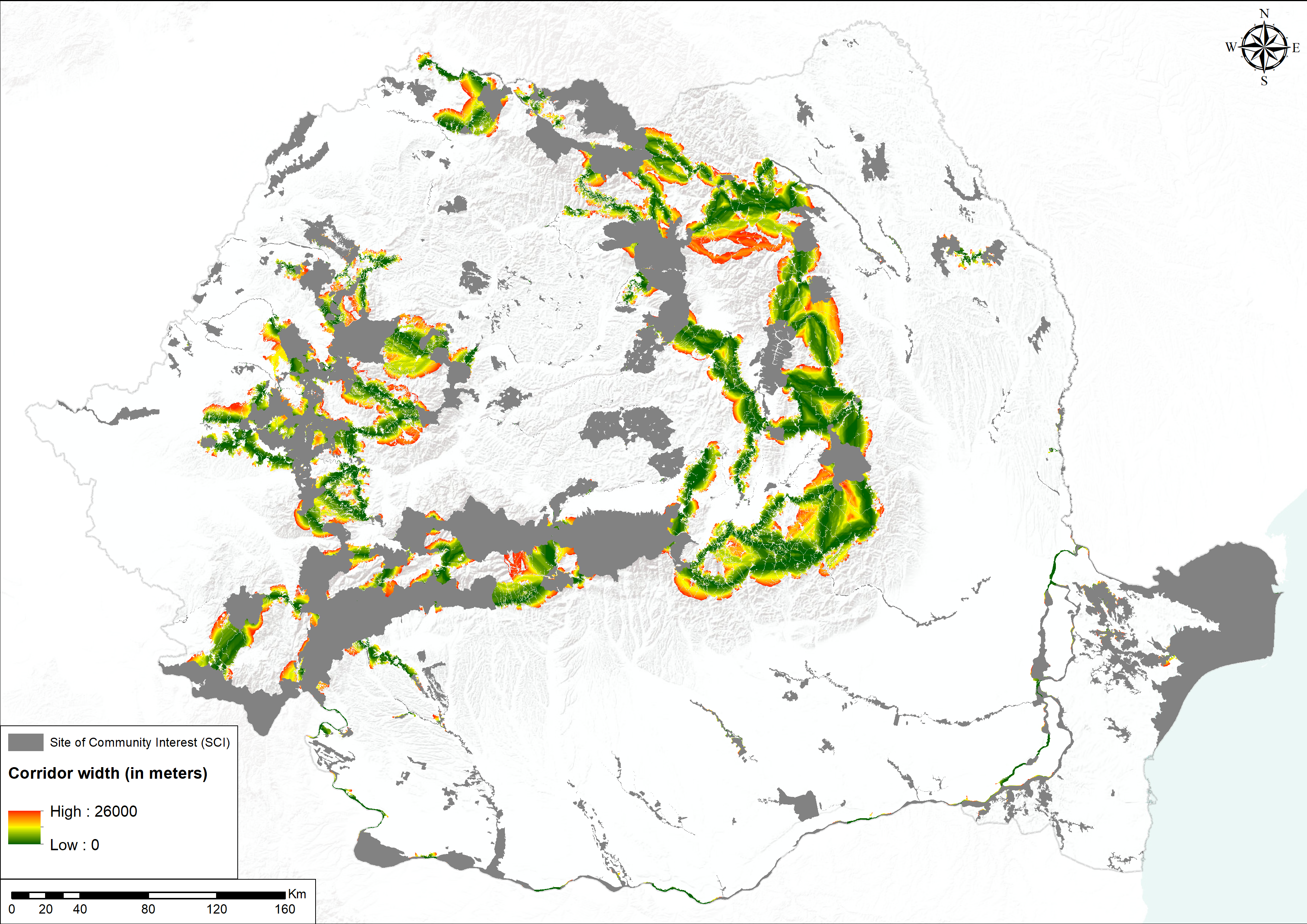

Supplement: Supplementary file 2 — Supplementary information 2. [file 41598_2020_76596_MOESM2_ESM.zip › Supplementary Material S2 Maps/Figure 14 Corridors for Hyla arborea.png]

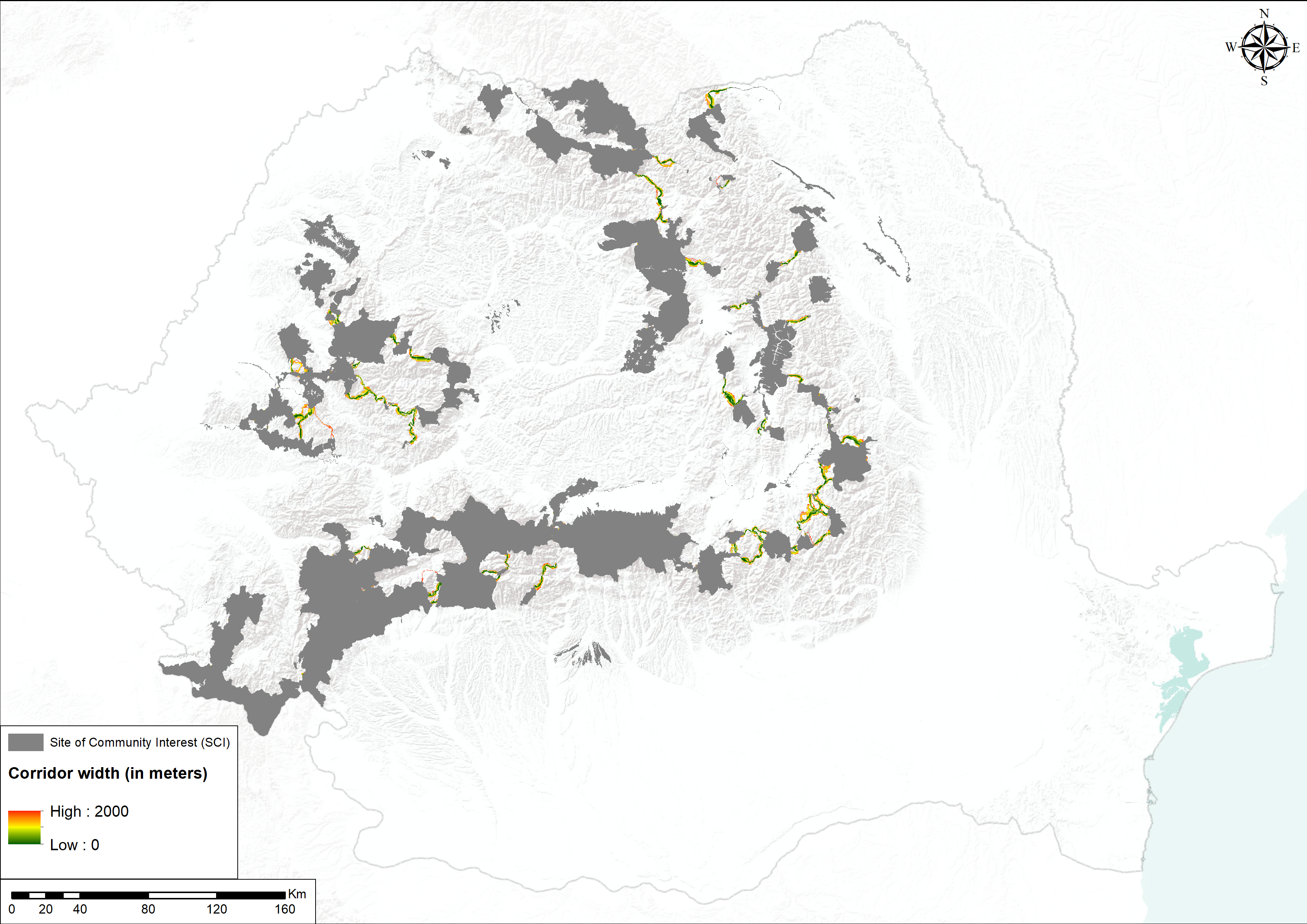

Supplement: Supplementary file 2 — Supplementary information 2. [file 41598_2020_76596_MOESM2_ESM.zip › Supplementary Material S2 Maps/Figure 15 Corridors for Ichthyosaura alpestris.png]

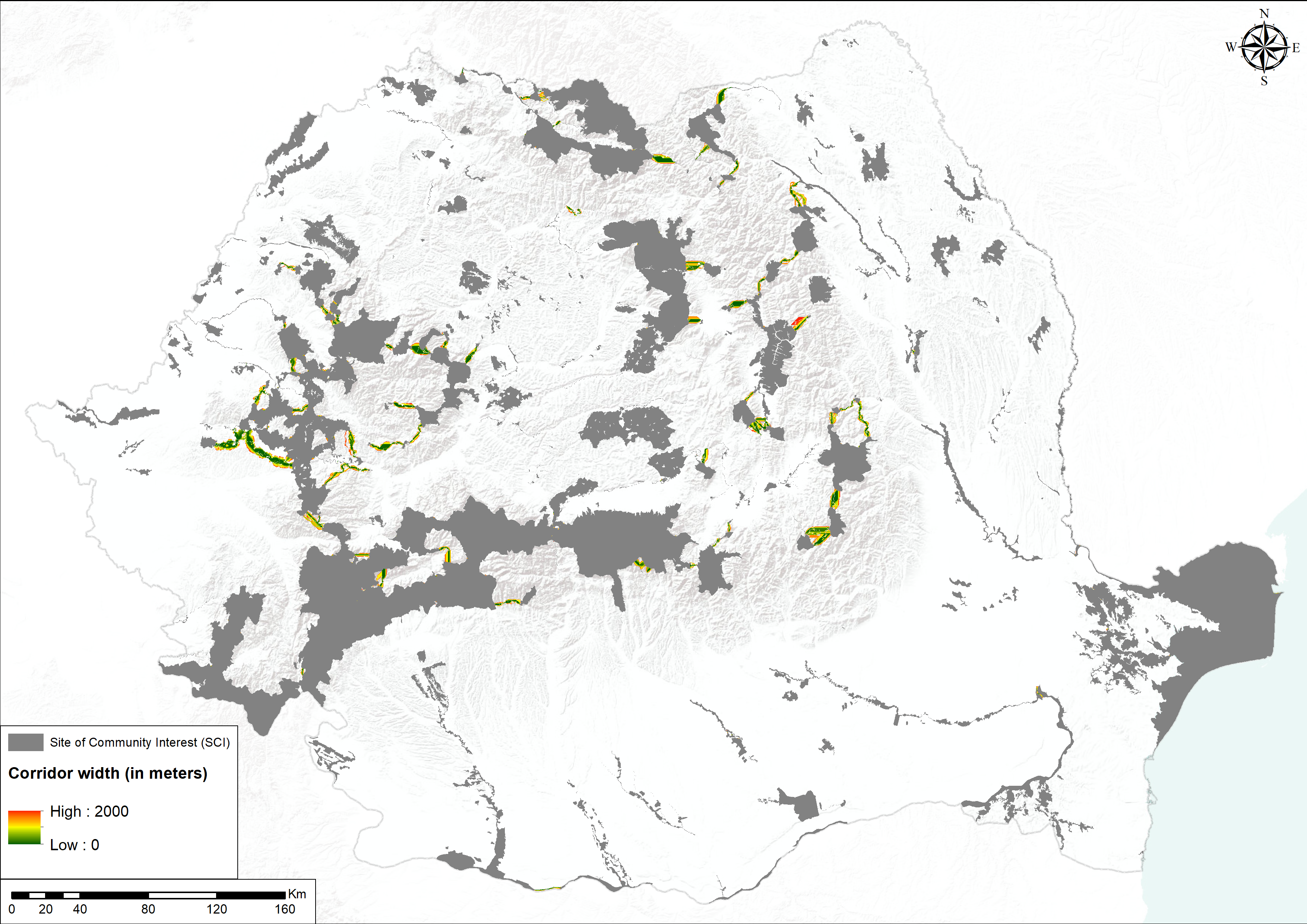

Supplement: Supplementary file 2 — Supplementary information 2. [file 41598_2020_76596_MOESM2_ESM.zip › Supplementary Material S2 Maps/Figure 16 Corridors for Lacerta agilis.png]

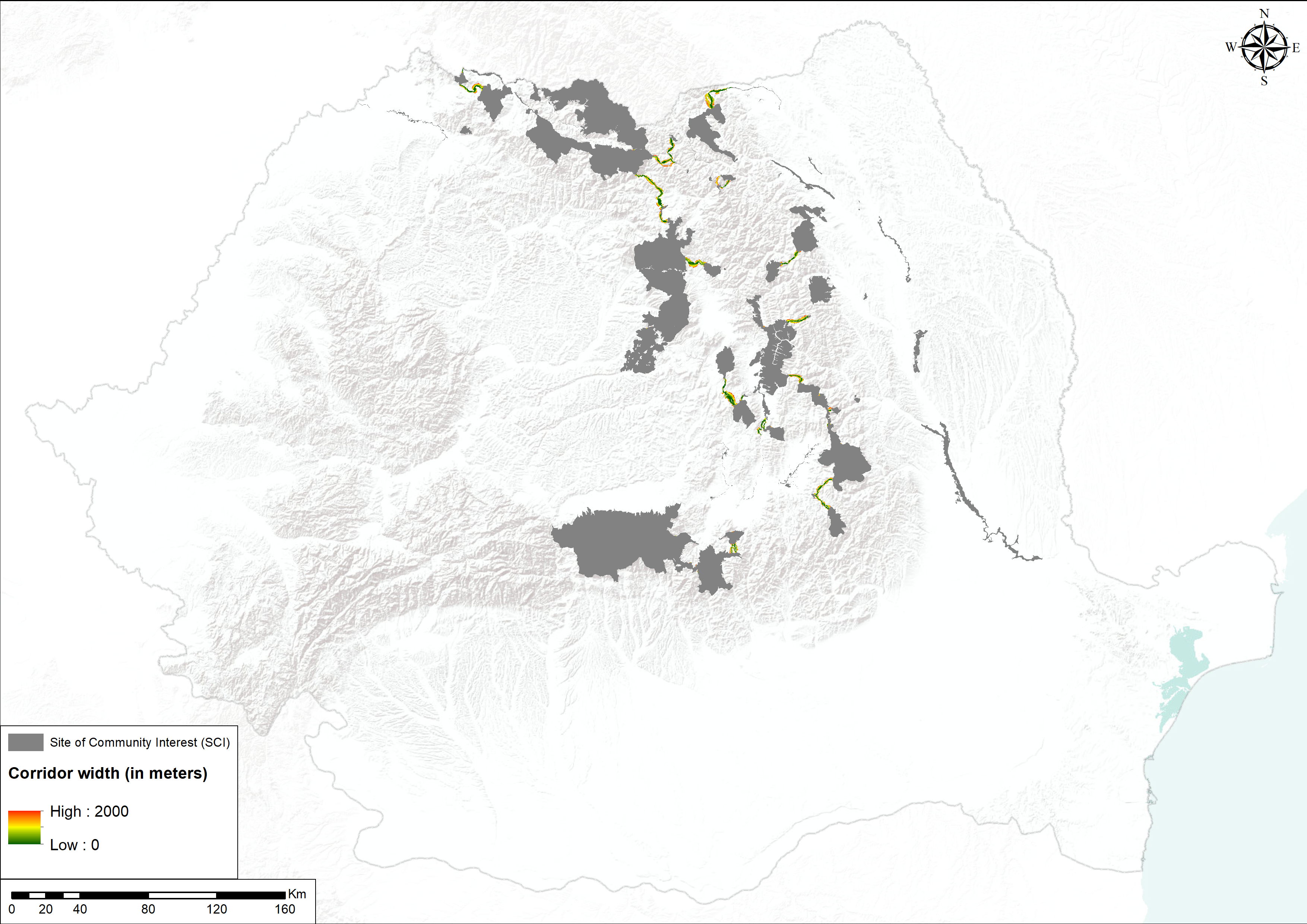

Supplement: Supplementary file 2 — Supplementary information 2. [file 41598_2020_76596_MOESM2_ESM.zip › Supplementary Material S2 Maps/Figure 17 Corridors for Lissotriton montandoni.png]

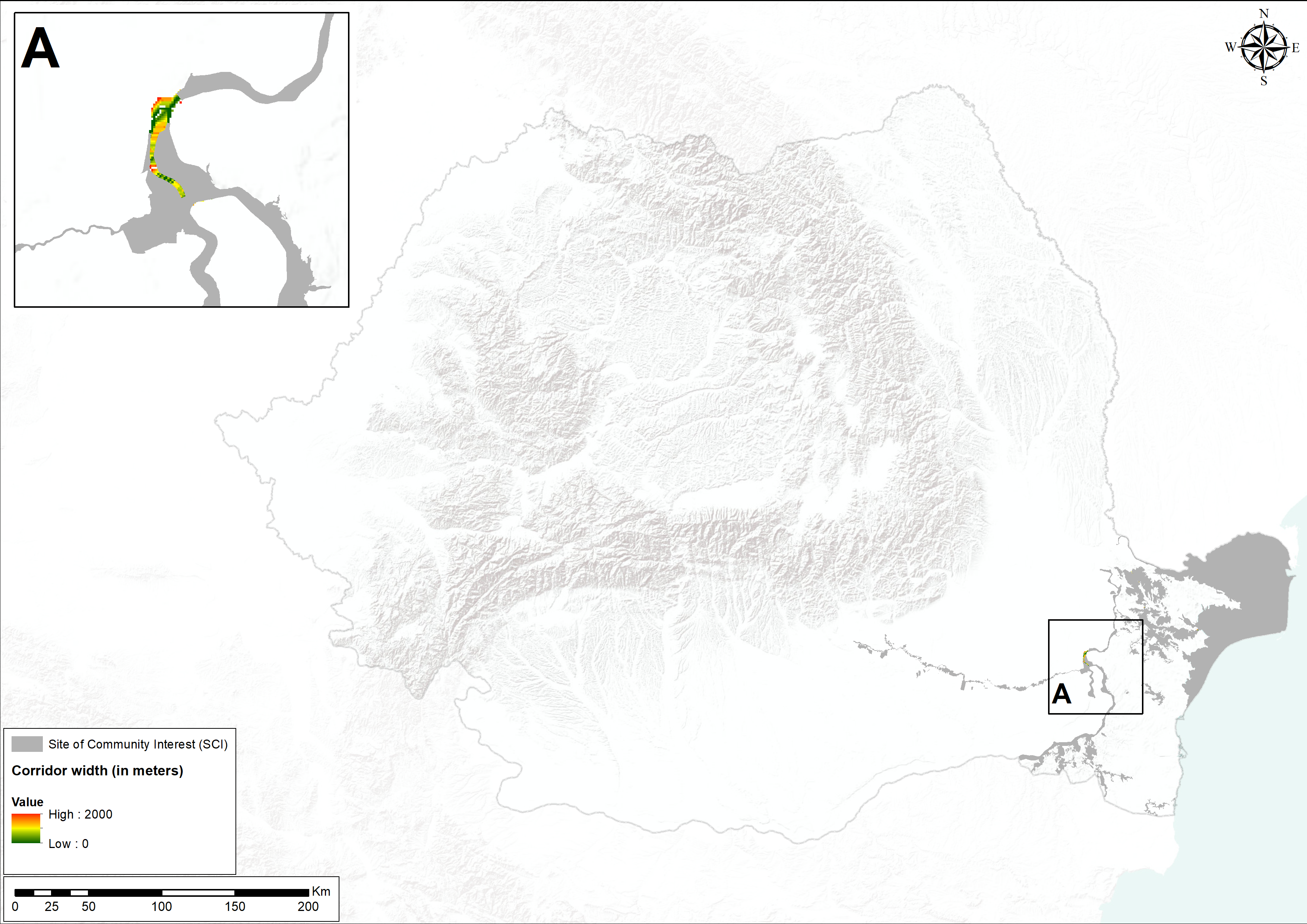

Supplement: Supplementary file 2 — Supplementary information 2. [file 41598_2020_76596_MOESM2_ESM.zip › Supplementary Material S2 Maps/Figure 18 Corridors for Lacerta trilineata.png]

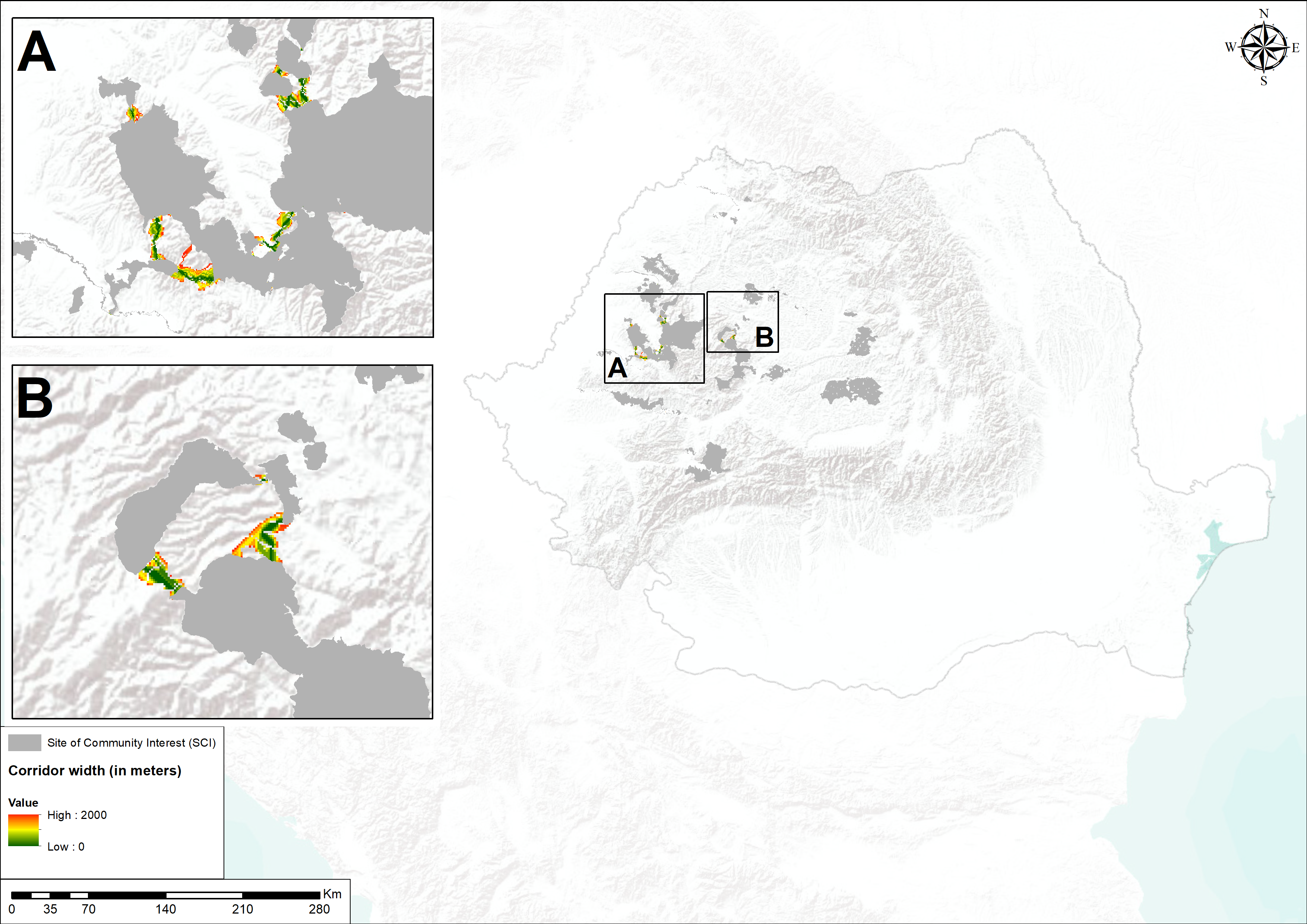

Supplement: Supplementary file 2 — Supplementary information 2. [file 41598_2020_76596_MOESM2_ESM.zip › Supplementary Material S2 Maps/Figure 19 Corridors for Lissotriton vulgaris ampelensis.png]

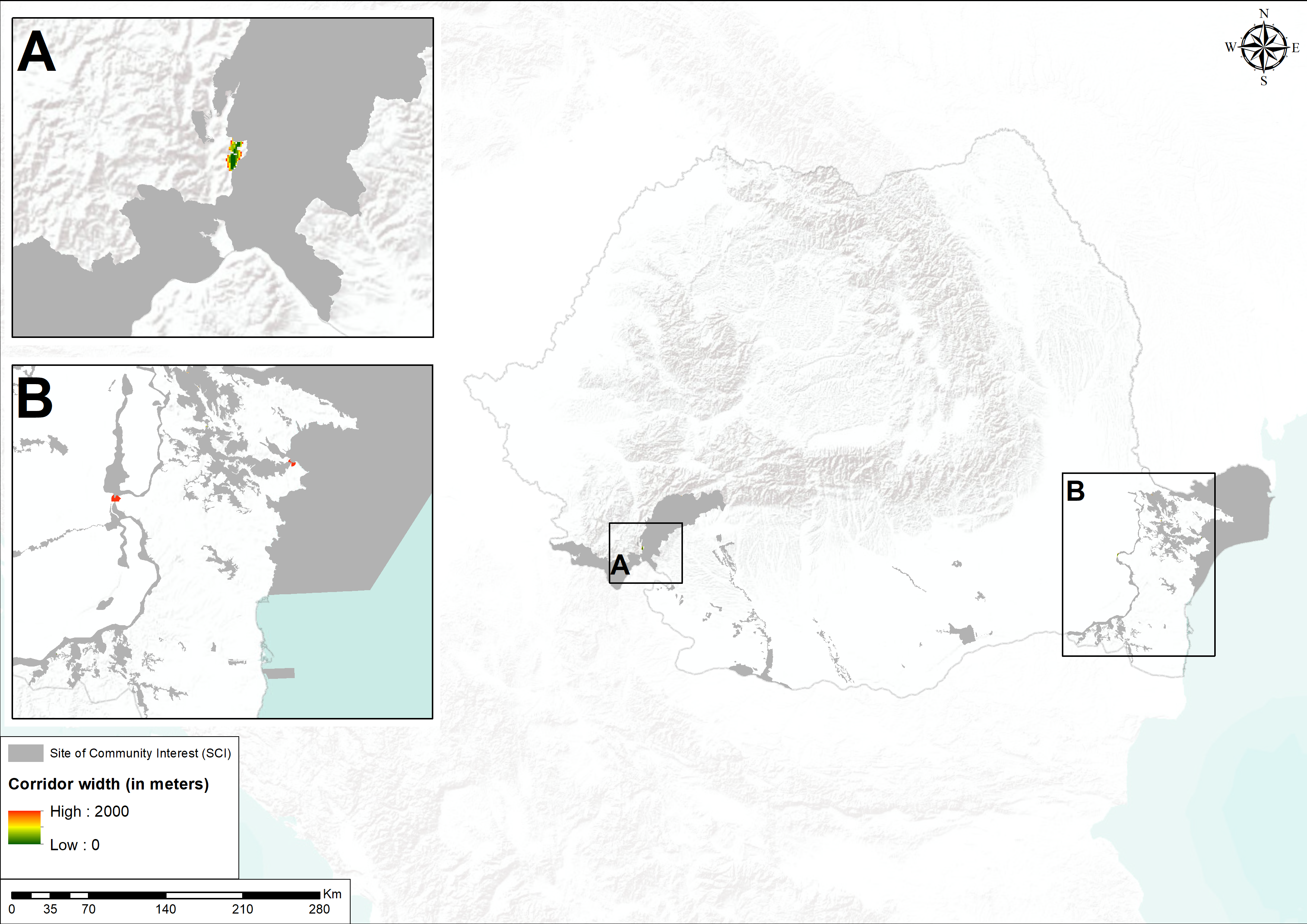

Supplement: Supplementary file 2 — Supplementary information 2. [file 41598_2020_76596_MOESM2_ESM.zip › Supplementary Material S2 Maps/Figure 2 Corridors for Ablepharus kitaibelii.png]

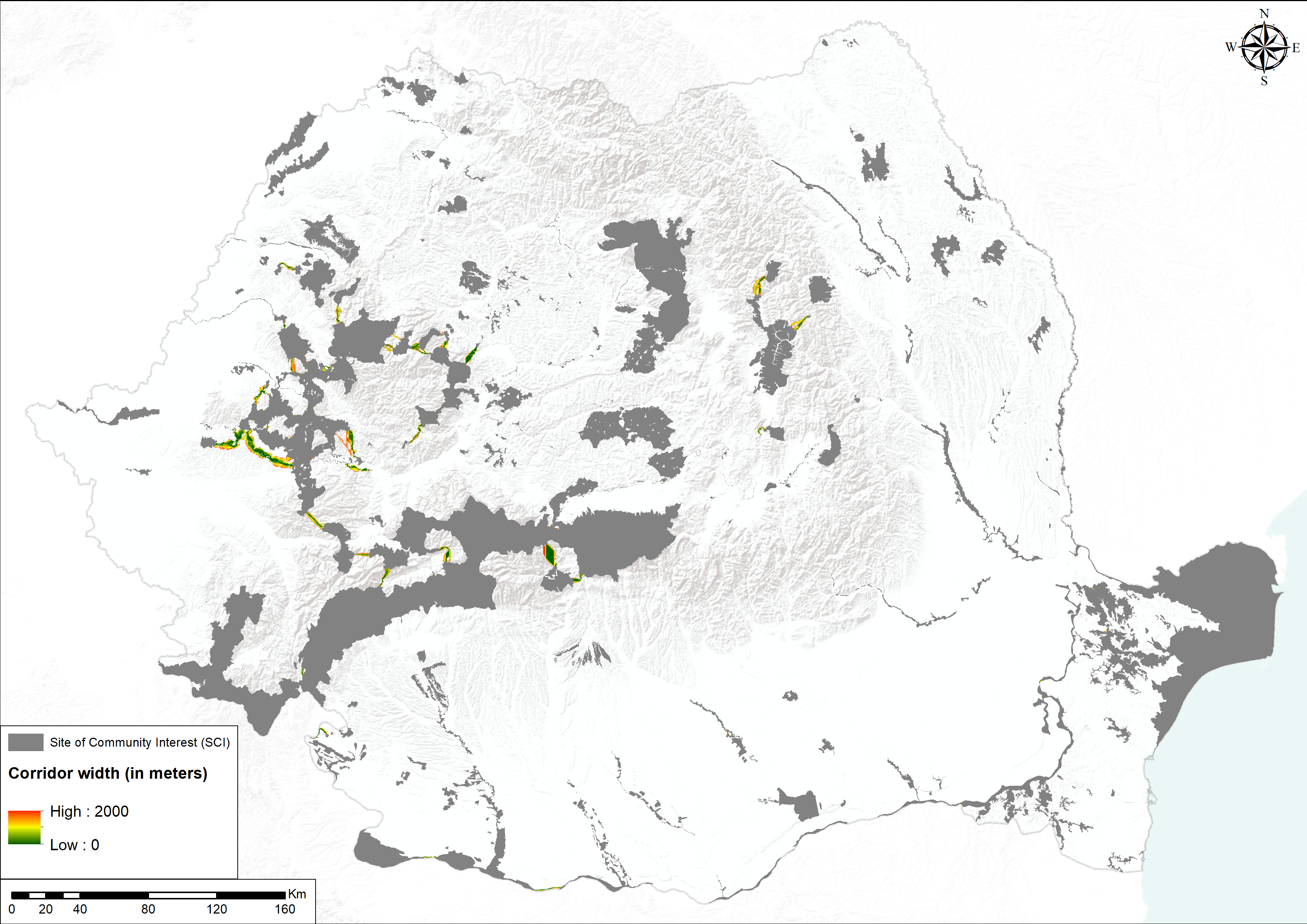

Supplement: Supplementary file 2 — Supplementary information 2. [file 41598_2020_76596_MOESM2_ESM.zip › Supplementary Material S2 Maps/Figure 20 Corridors for Lacerta viridis.png]

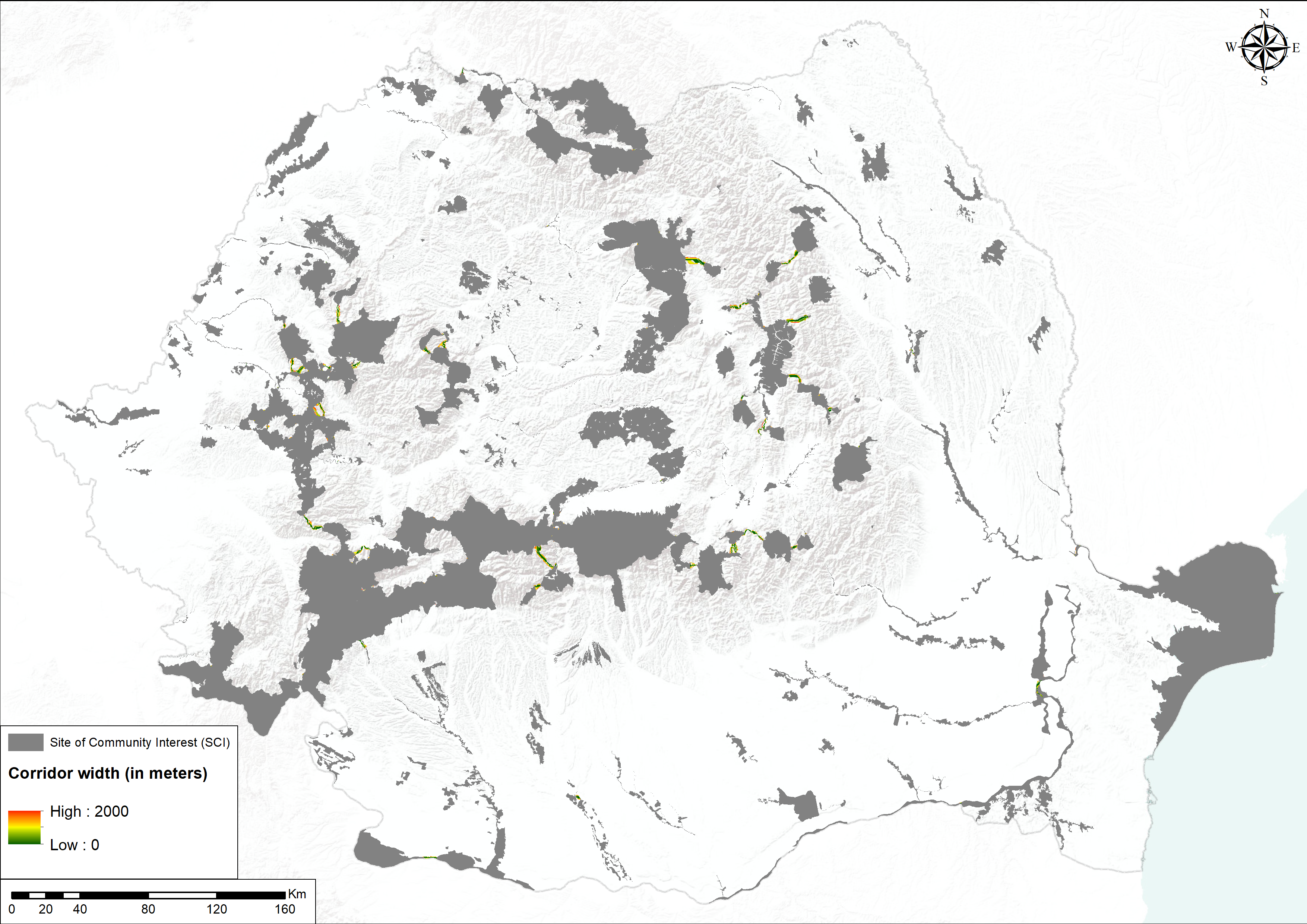

Supplement: Supplementary file 2 — Supplementary information 2. [file 41598_2020_76596_MOESM2_ESM.zip › Supplementary Material S2 Maps/Figure 21 Corridors for Lissotriton vulgaris vulgaris.png]

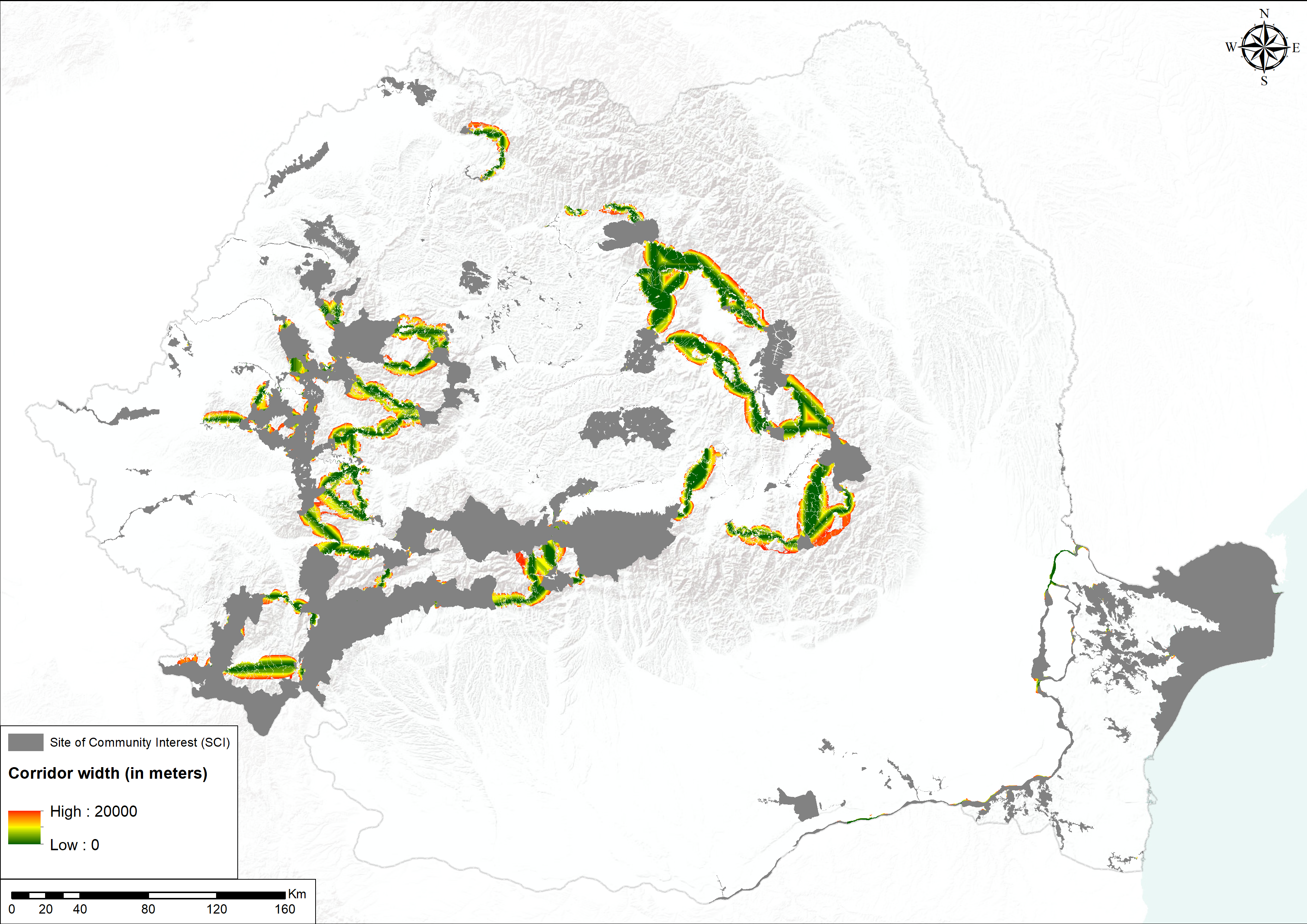

Supplement: Supplementary file 2 — Supplementary information 2. [file 41598_2020_76596_MOESM2_ESM.zip › Supplementary Material S2 Maps/Figure 22 Corridors for Natrix tessellata.png]

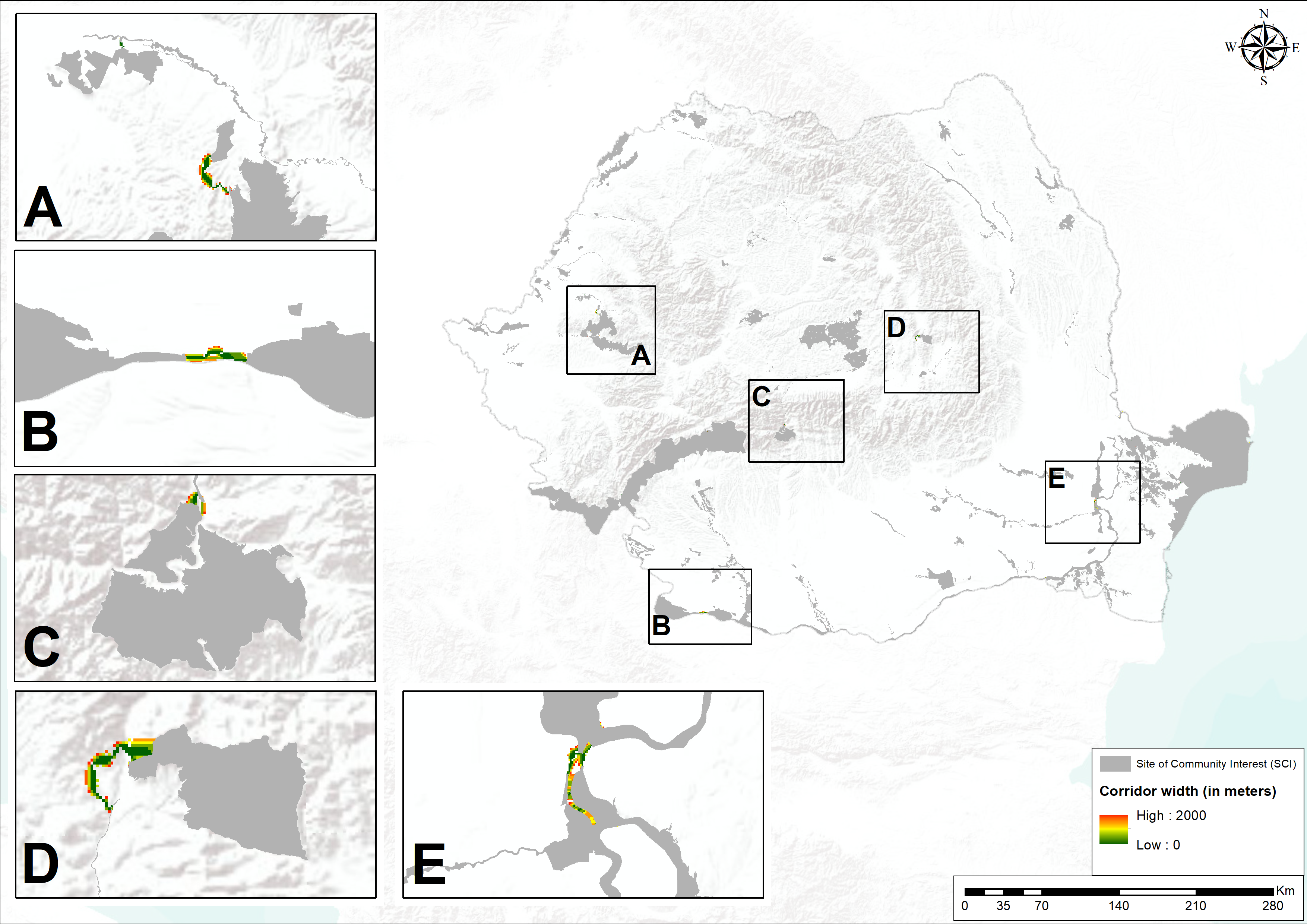

Supplement: Supplementary file 2 — Supplementary information 2. [file 41598_2020_76596_MOESM2_ESM.zip › Supplementary Material S2 Maps/Figure 23 Corridors for Pelobates fuscus.png]

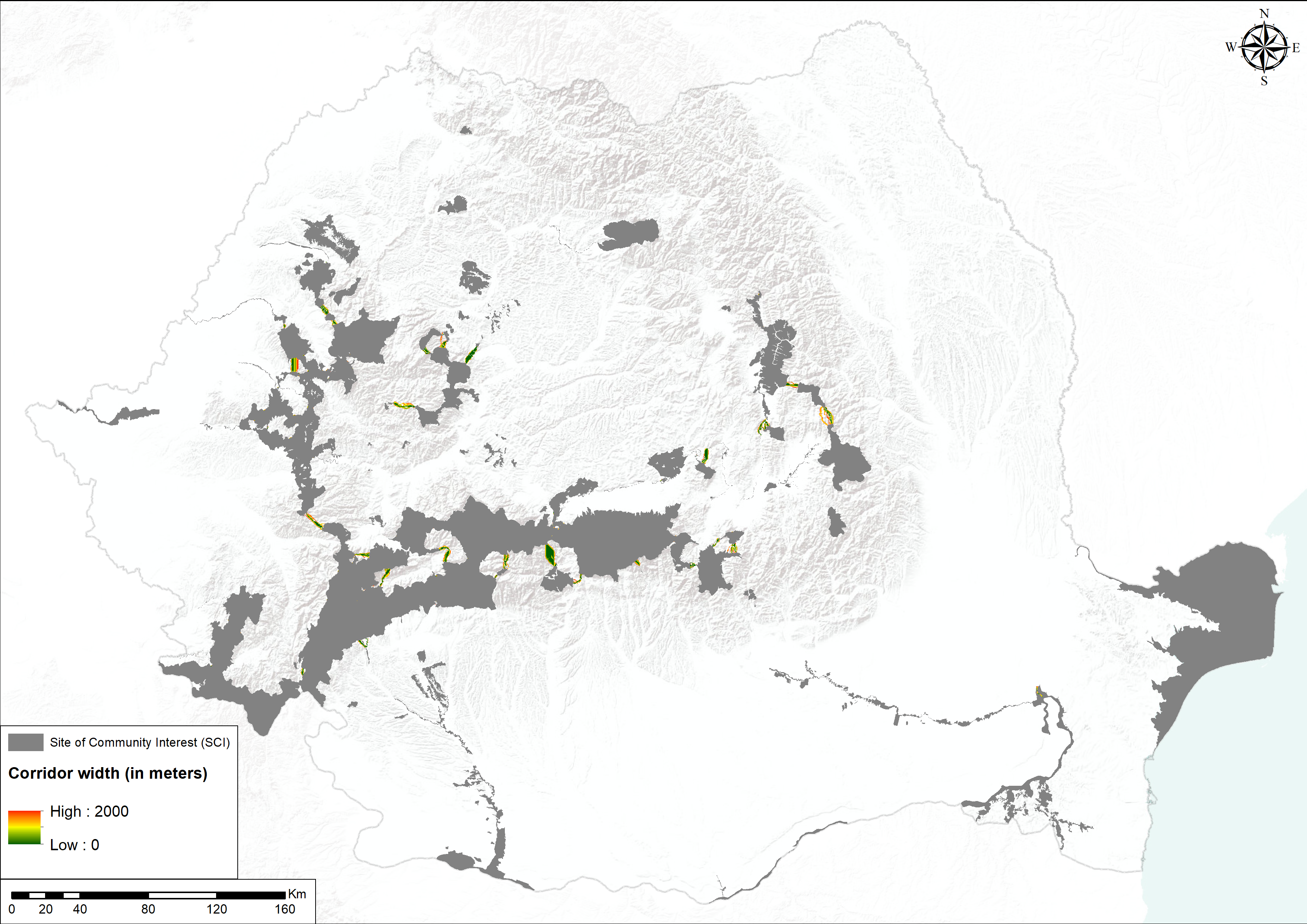

Supplement: Supplementary file 2 — Supplementary information 2. [file 41598_2020_76596_MOESM2_ESM.zip › Supplementary Material S2 Maps/Figure 24 Corridors for Podarcis muralis.png]

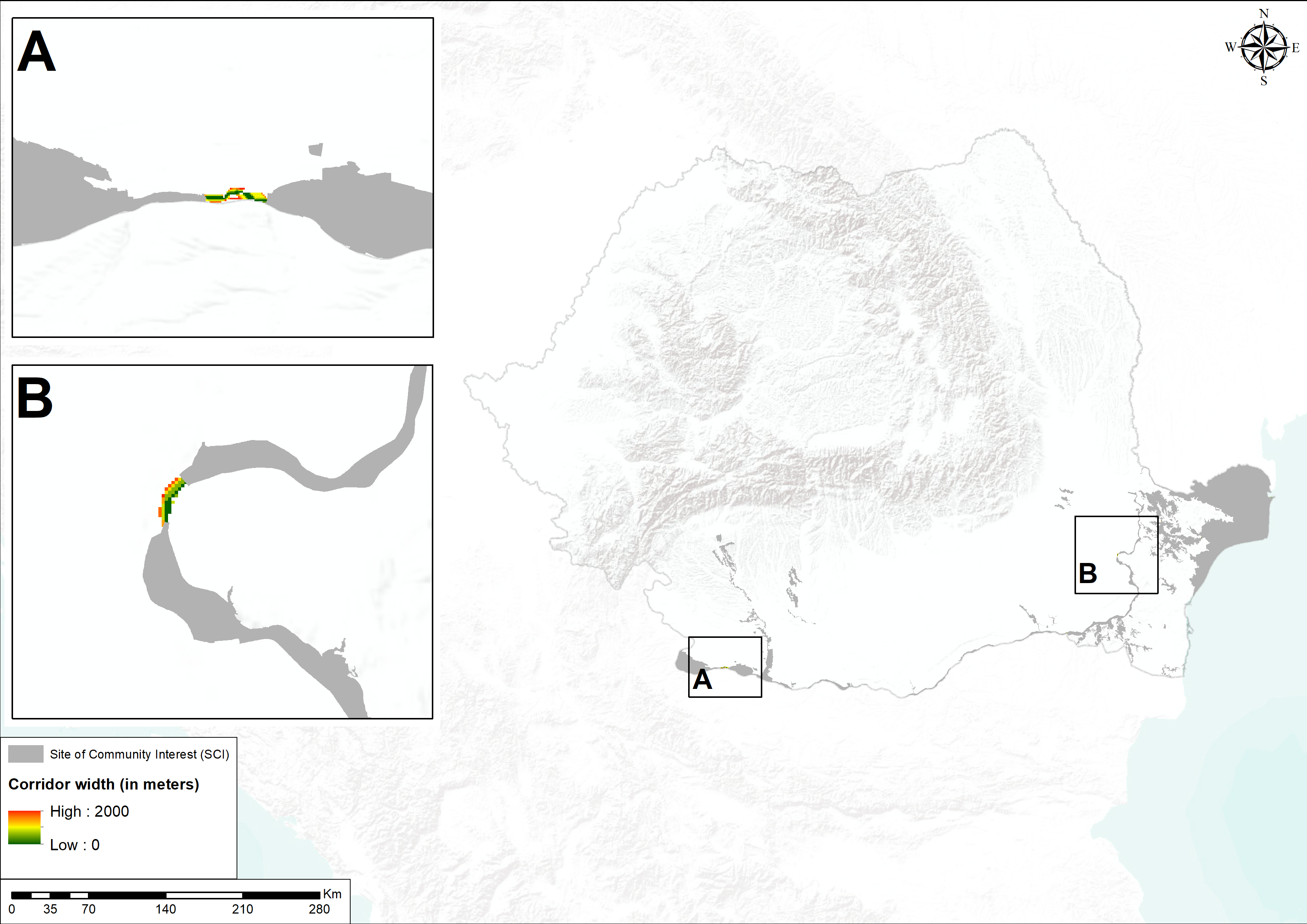

Supplement: Supplementary file 2 — Supplementary information 2. [file 41598_2020_76596_MOESM2_ESM.zip › Supplementary Material S2 Maps/Figure 25 Corridors for Pelobates syriacus.png]

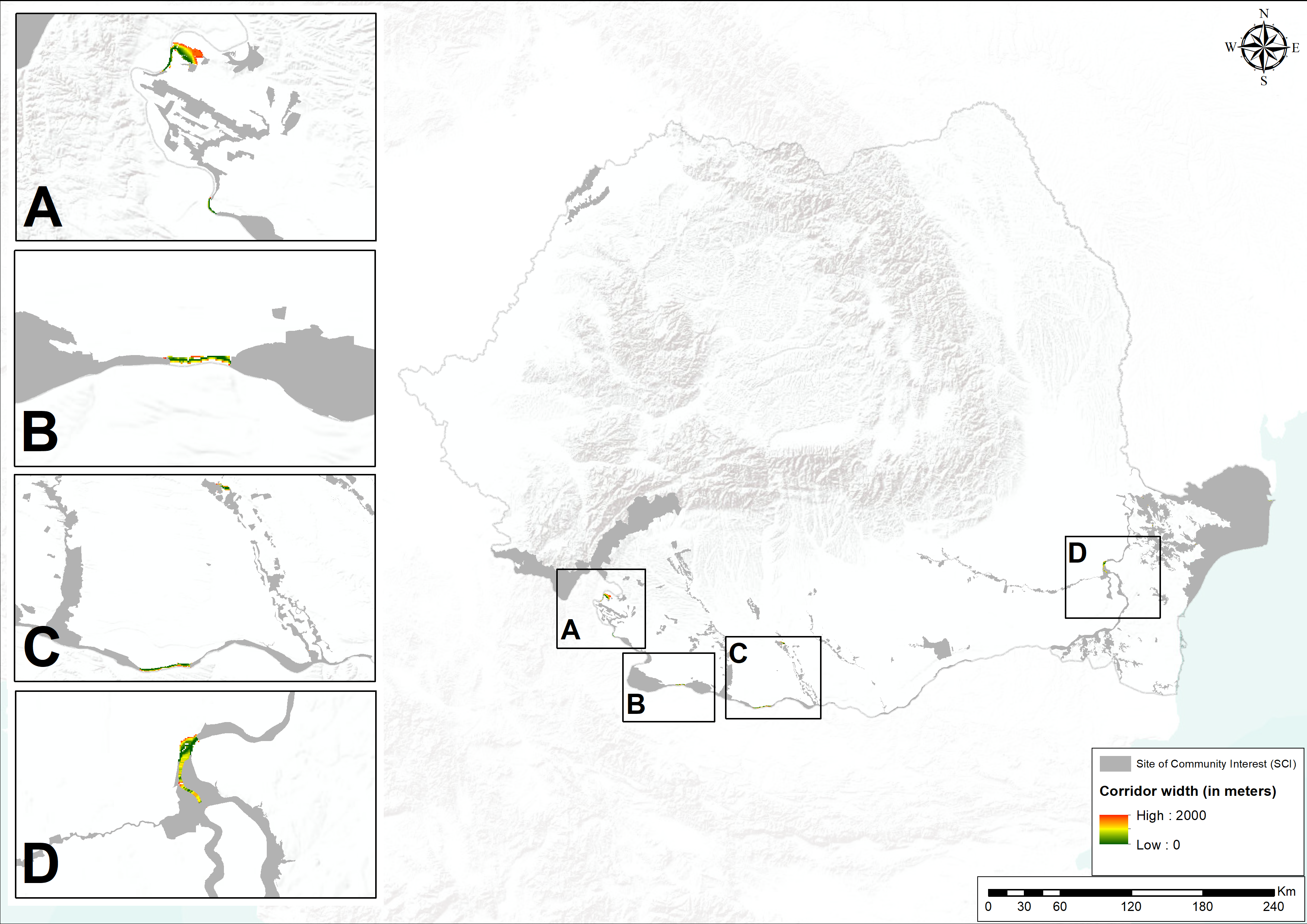

Supplement: Supplementary file 2 — Supplementary information 2. [file 41598_2020_76596_MOESM2_ESM.zip › Supplementary Material S2 Maps/Figure 26 Corridors for Podarcis tauricus.png]

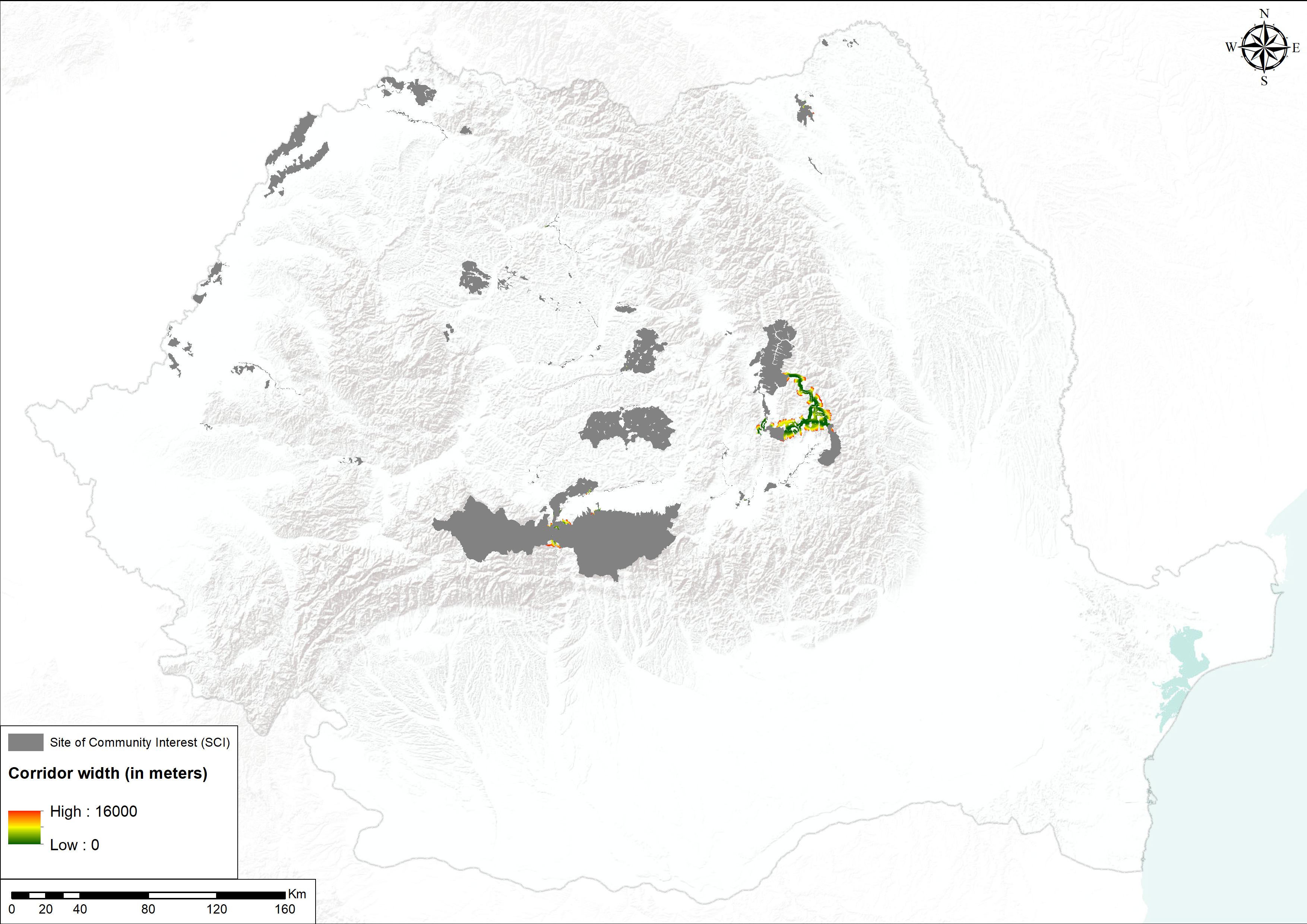

Supplement: Supplementary file 2 — Supplementary information 2. [file 41598_2020_76596_MOESM2_ESM.zip › Supplementary Material S2 Maps/Figure 27 Corridors for Rana arvalis.png]

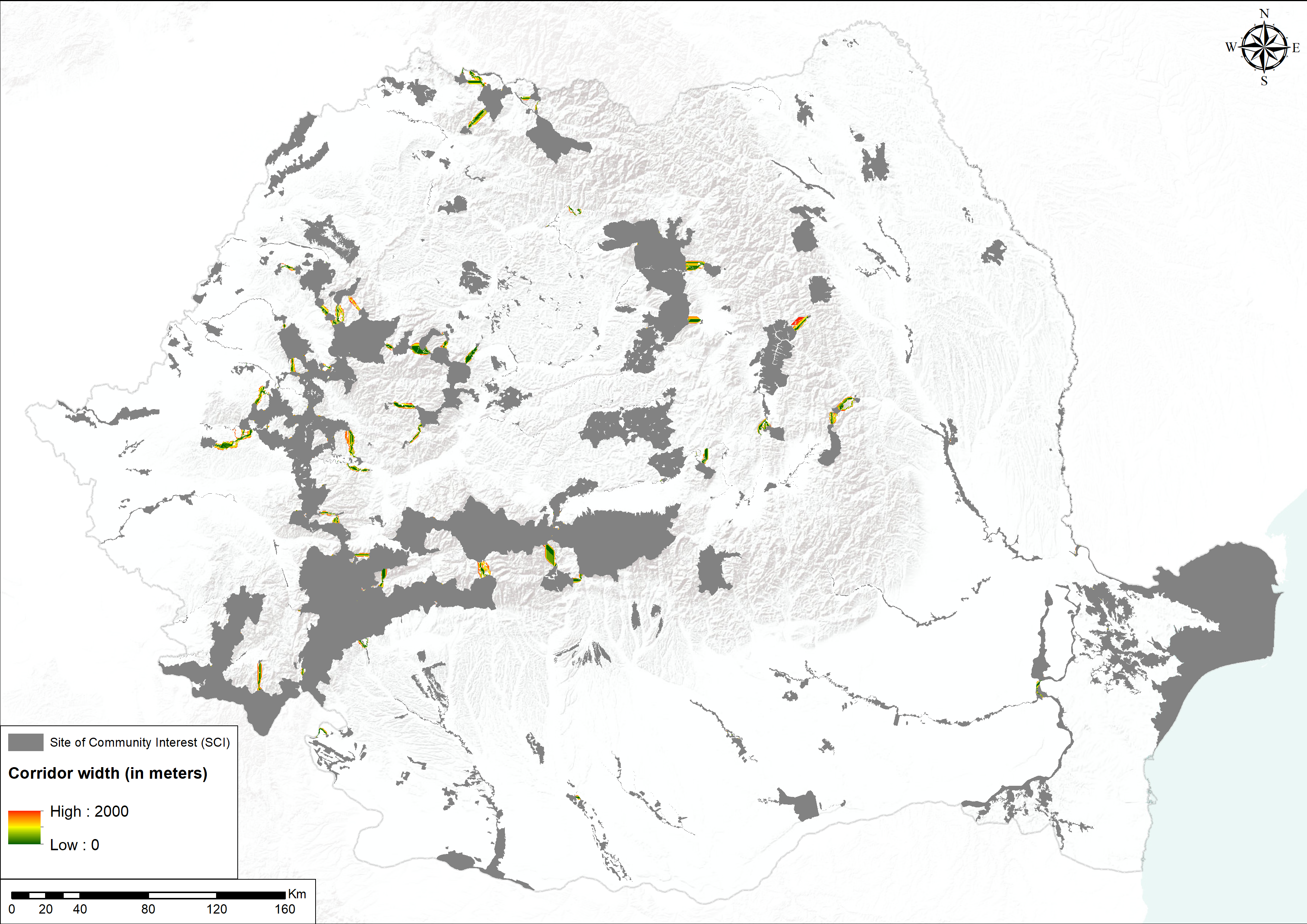

Supplement: Supplementary file 2 — Supplementary information 2. [file 41598_2020_76596_MOESM2_ESM.zip › Supplementary Material S2 Maps/Figure 28 Corridors for Rana dalmatina.png]

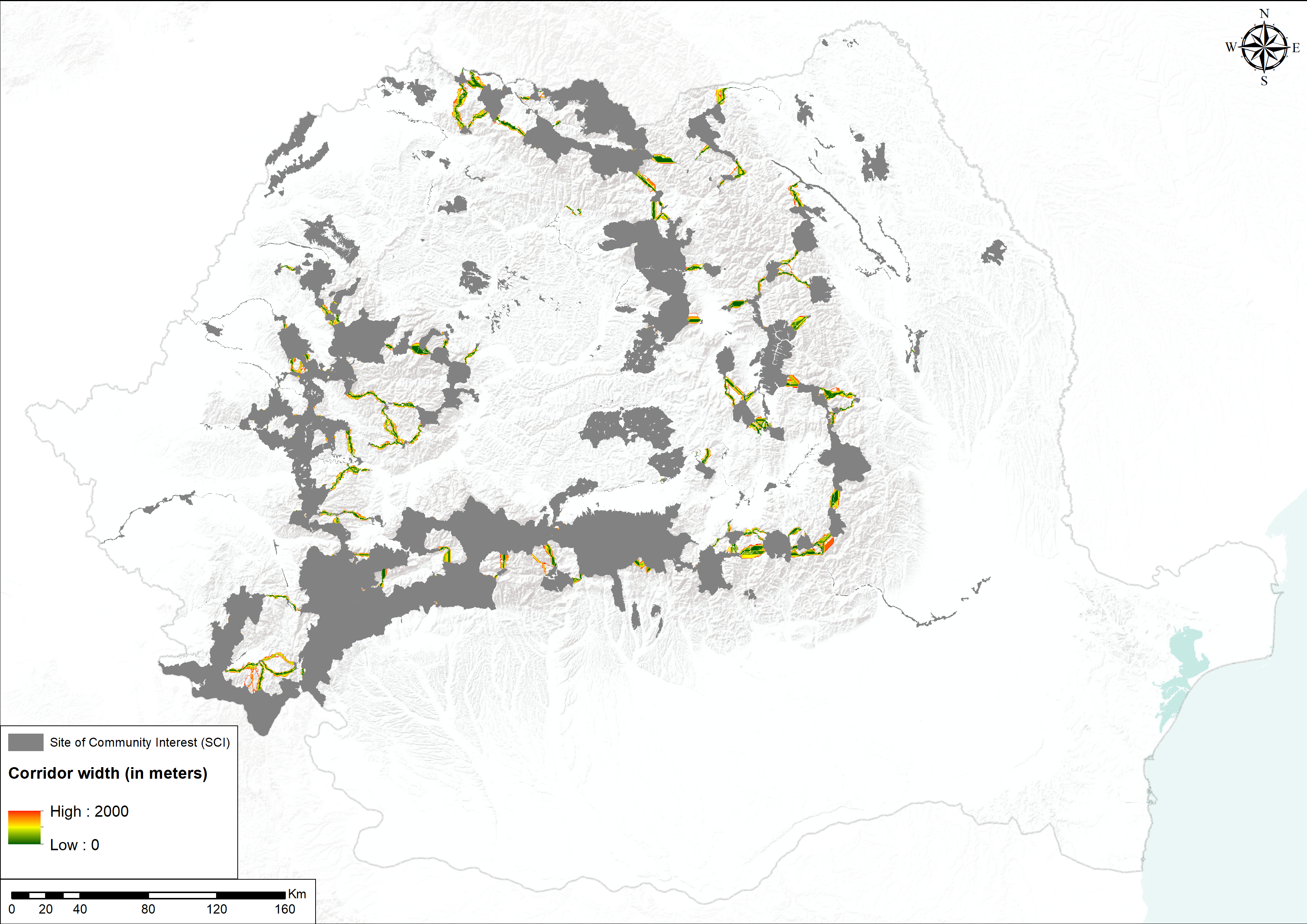

Supplement: Supplementary file 2 — Supplementary information 2. [file 41598_2020_76596_MOESM2_ESM.zip › Supplementary Material S2 Maps/Figure 29 Corridors for Rana temporaria.png]

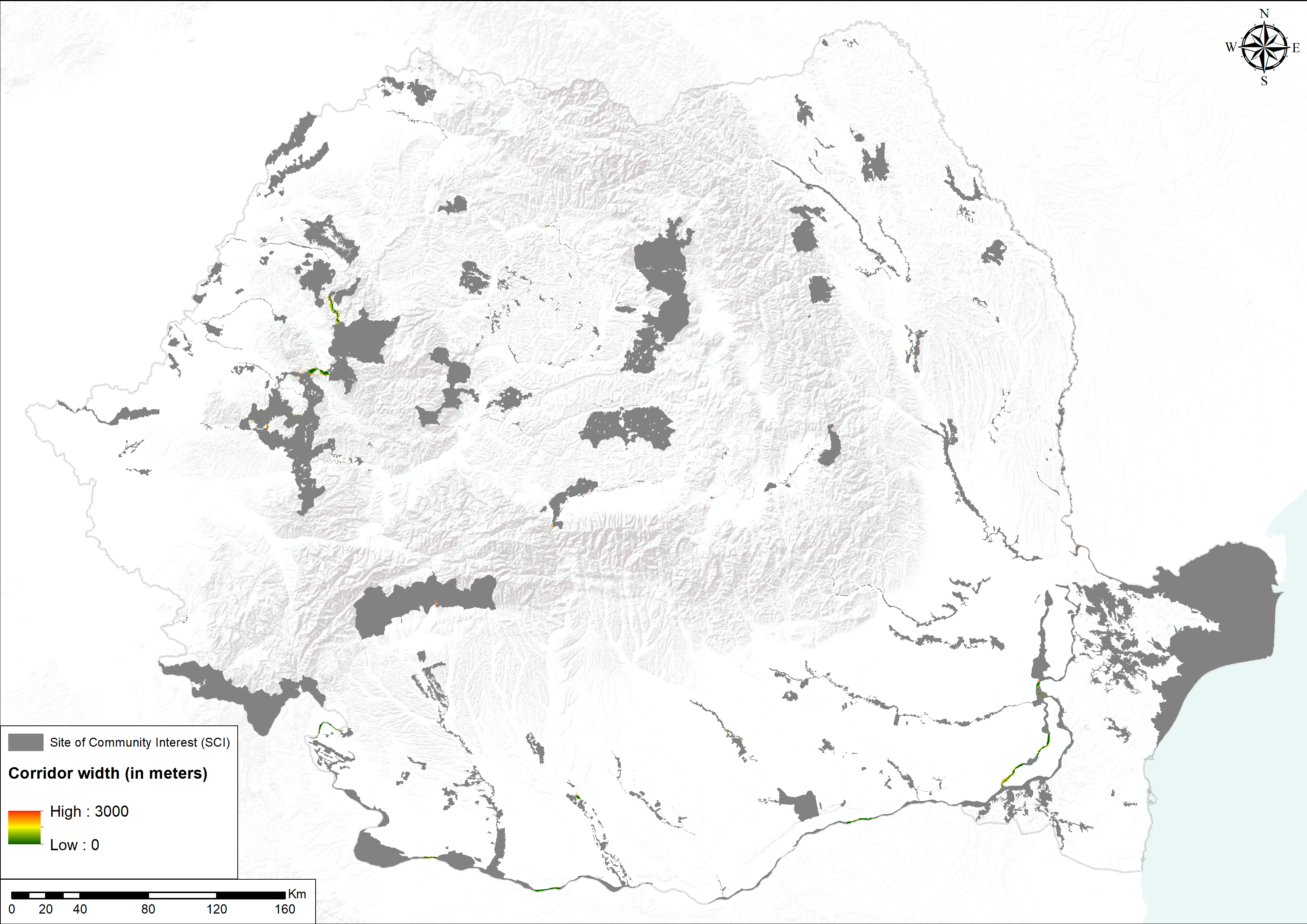

Supplement: Supplementary file 2 — Supplementary information 2. [file 41598_2020_76596_MOESM2_ESM.zip › Supplementary Material S2 Maps/Figure 3 Corridors for Bombina bombina.png]

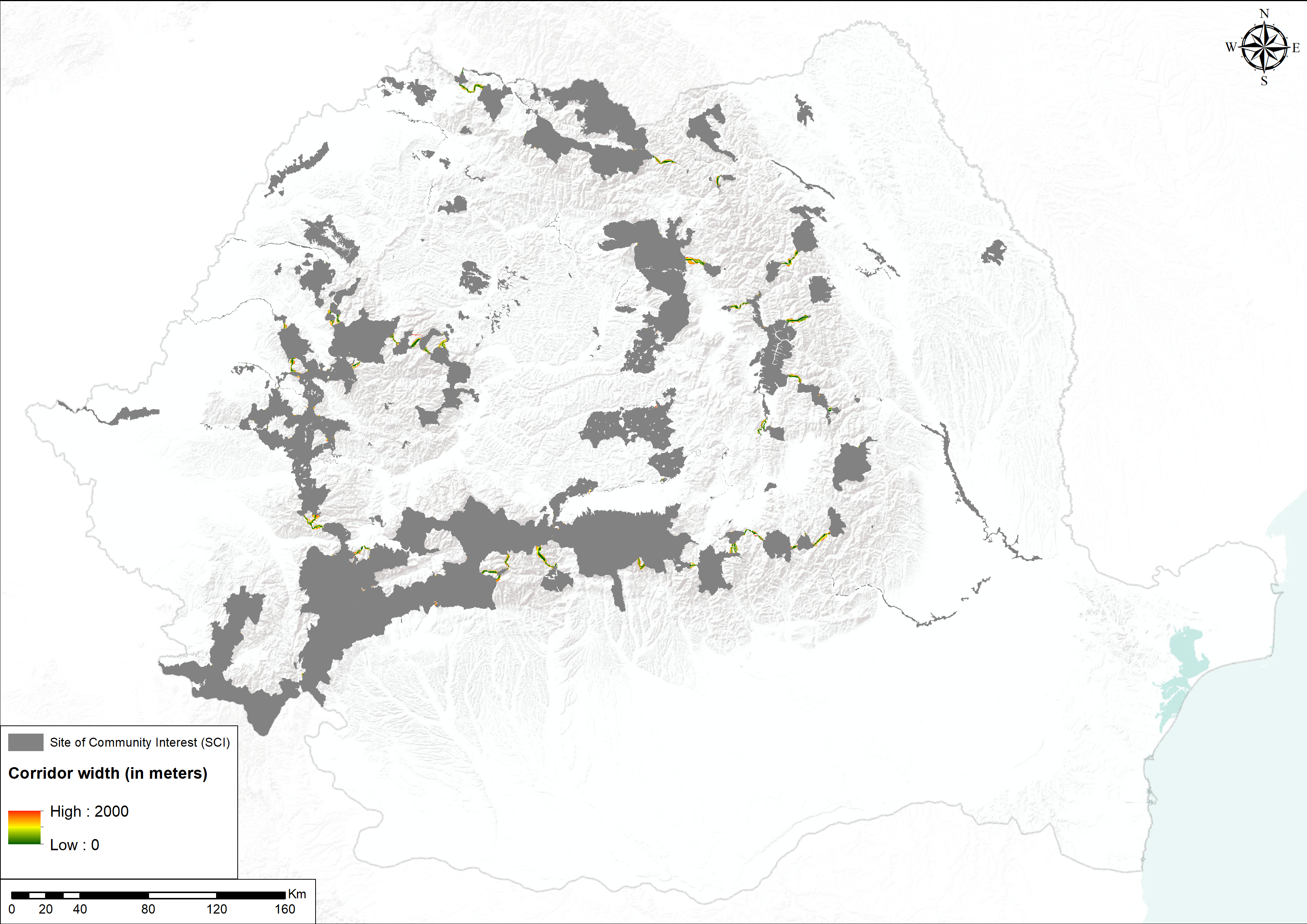

Supplement: Supplementary file 2 — Supplementary information 2. [file 41598_2020_76596_MOESM2_ESM.zip › Supplementary Material S2 Maps/Figure 30 Corridors for Salamandra salamandra.png]

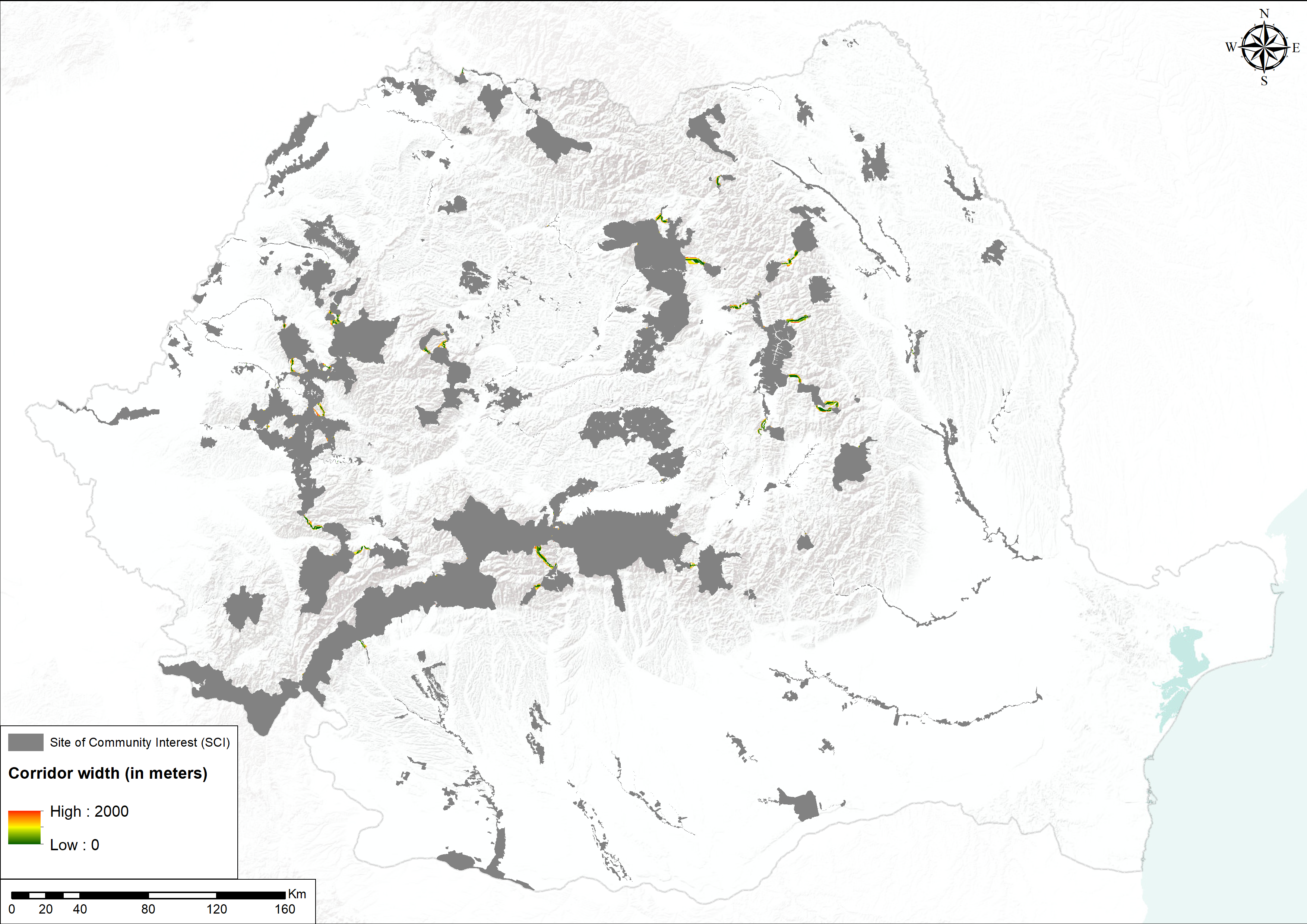

Supplement: Supplementary file 2 — Supplementary information 2. [file 41598_2020_76596_MOESM2_ESM.zip › Supplementary Material S2 Maps/Figure 31 Corridors for Triturus cristatus.png]

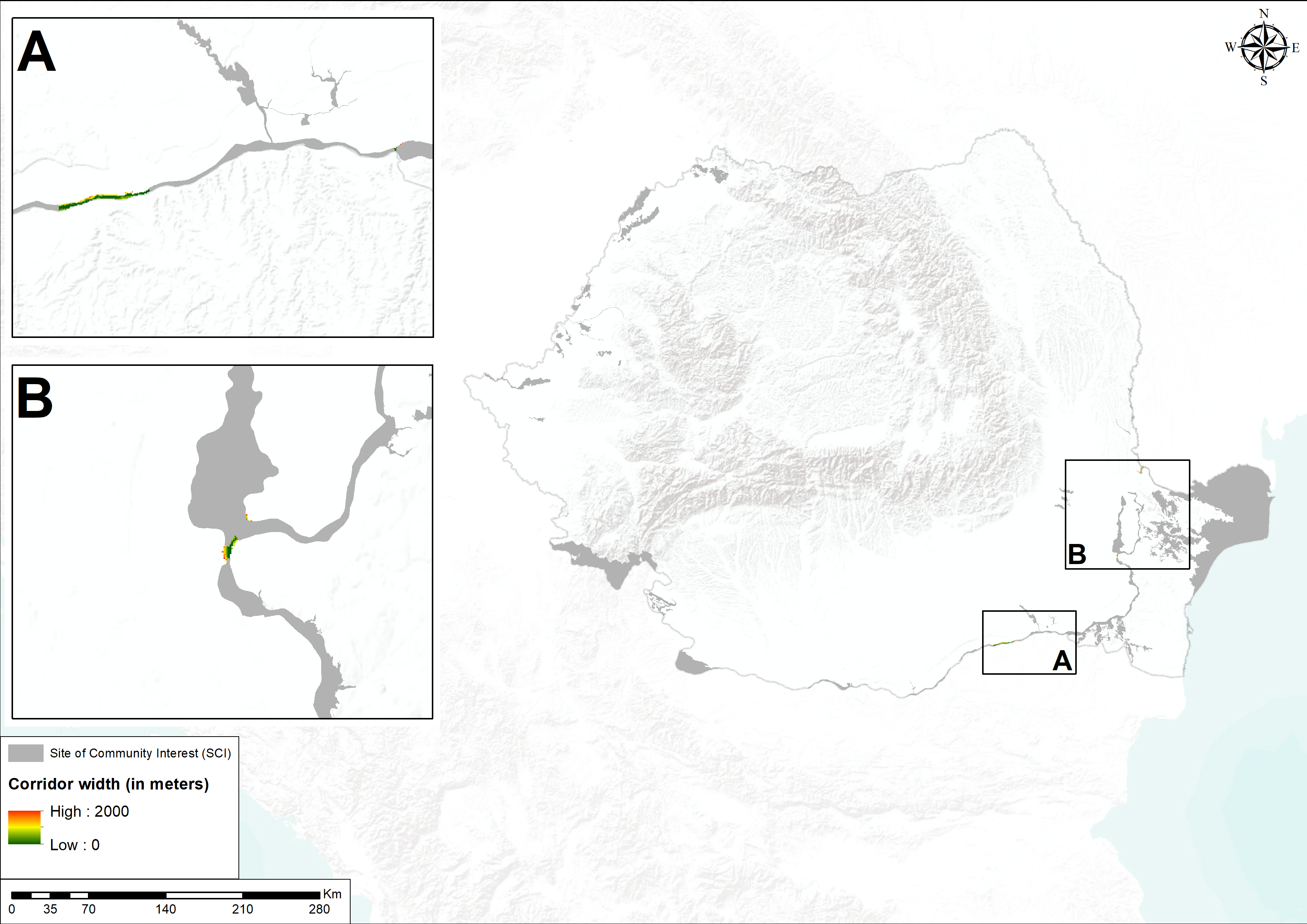

Supplement: Supplementary file 2 — Supplementary information 2. [file 41598_2020_76596_MOESM2_ESM.zip › Supplementary Material S2 Maps/Figure 32 Corridors for Triturus dobrogicus.png]

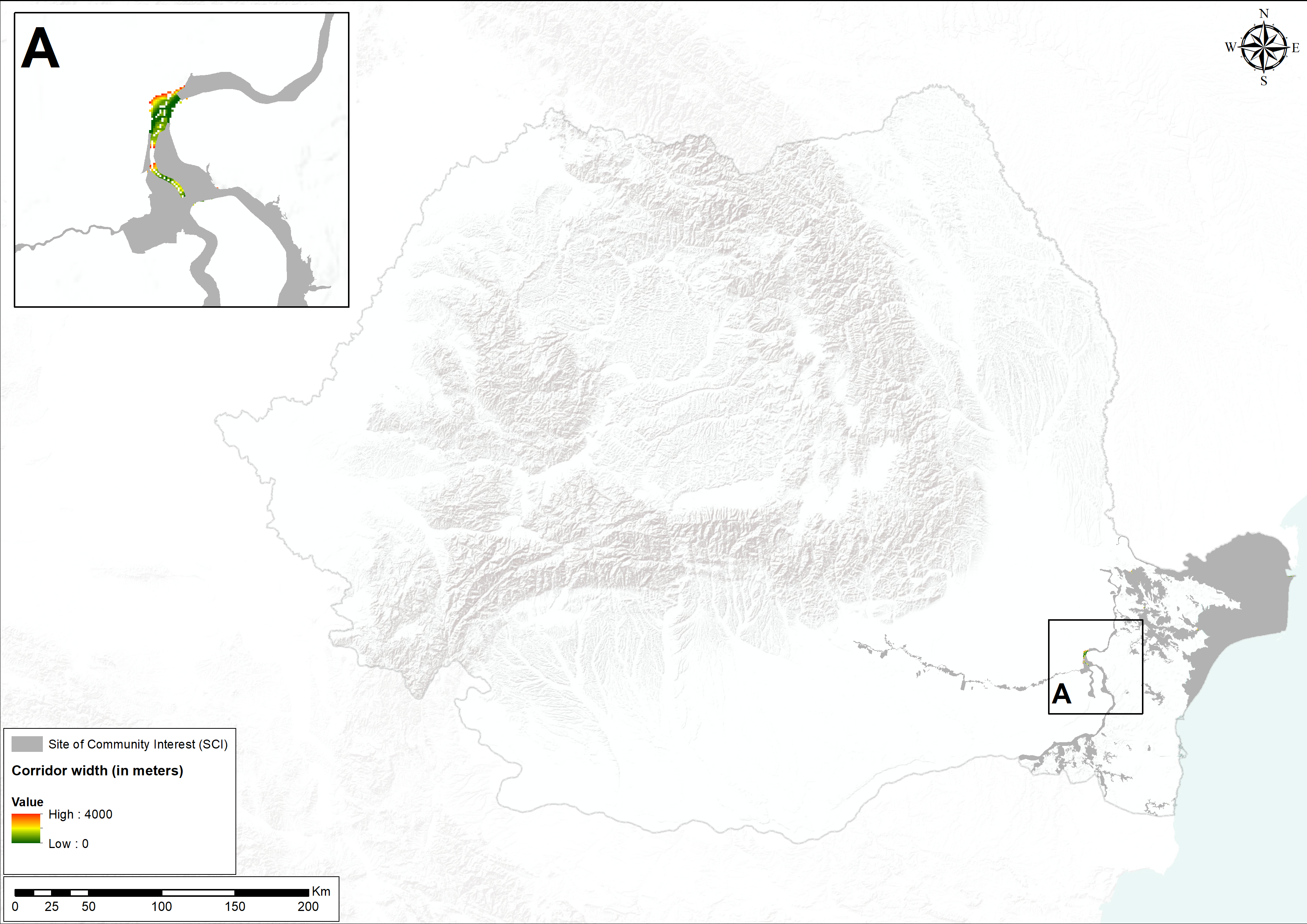

Supplement: Supplementary file 2 — Supplementary information 2. [file 41598_2020_76596_MOESM2_ESM.zip › Supplementary Material S2 Maps/Figure 33 Corridors for Testudo graeca.png]

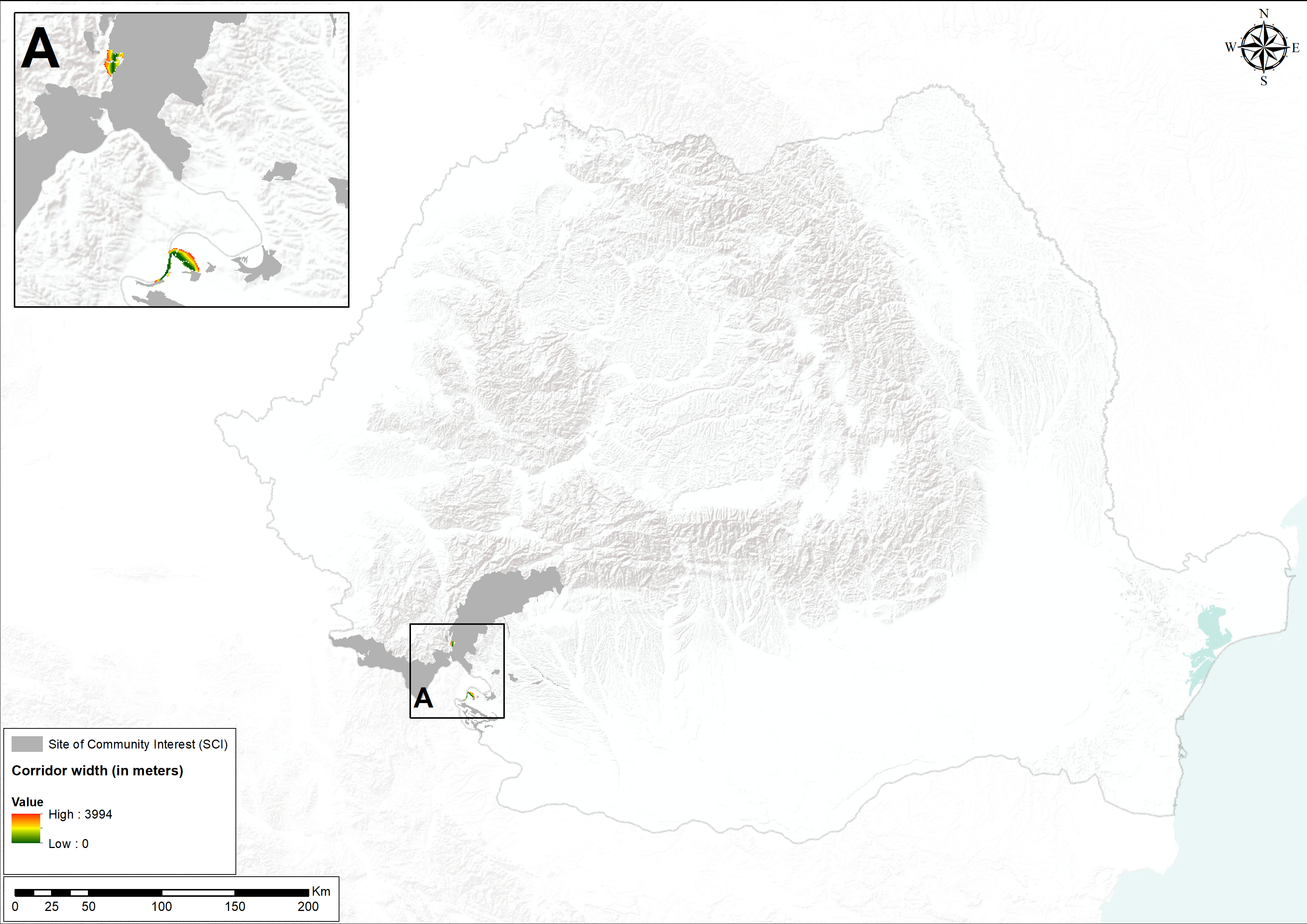

Supplement: Supplementary file 2 — Supplementary information 2. [file 41598_2020_76596_MOESM2_ESM.zip › Supplementary Material S2 Maps/Figure 34 Corridors for Testudo hermanni.png]

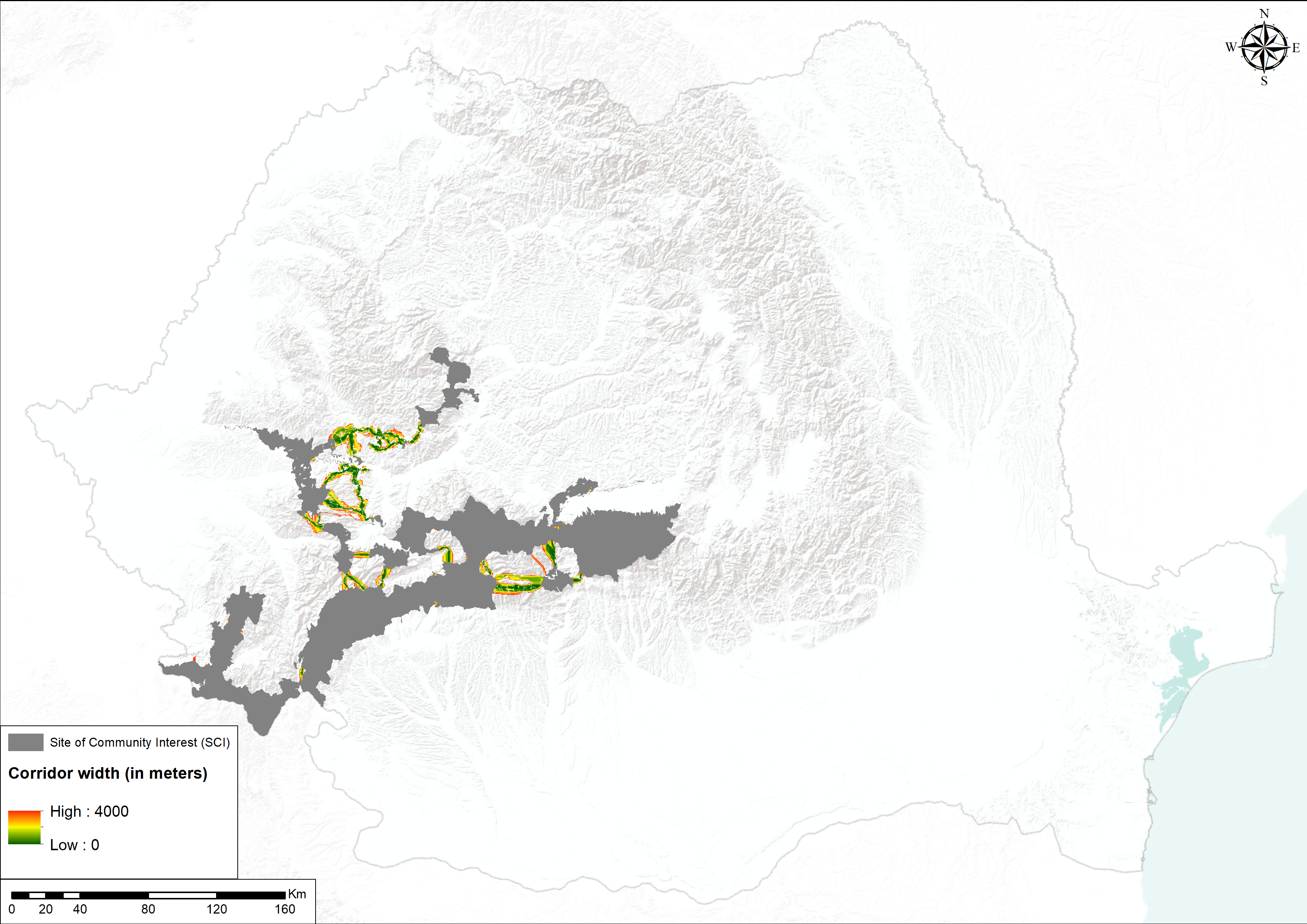

Supplement: Supplementary file 2 — Supplementary information 2. [file 41598_2020_76596_MOESM2_ESM.zip › Supplementary Material S2 Maps/Figure 35 Corridors for Vipera ammodyes ammodytes.png]

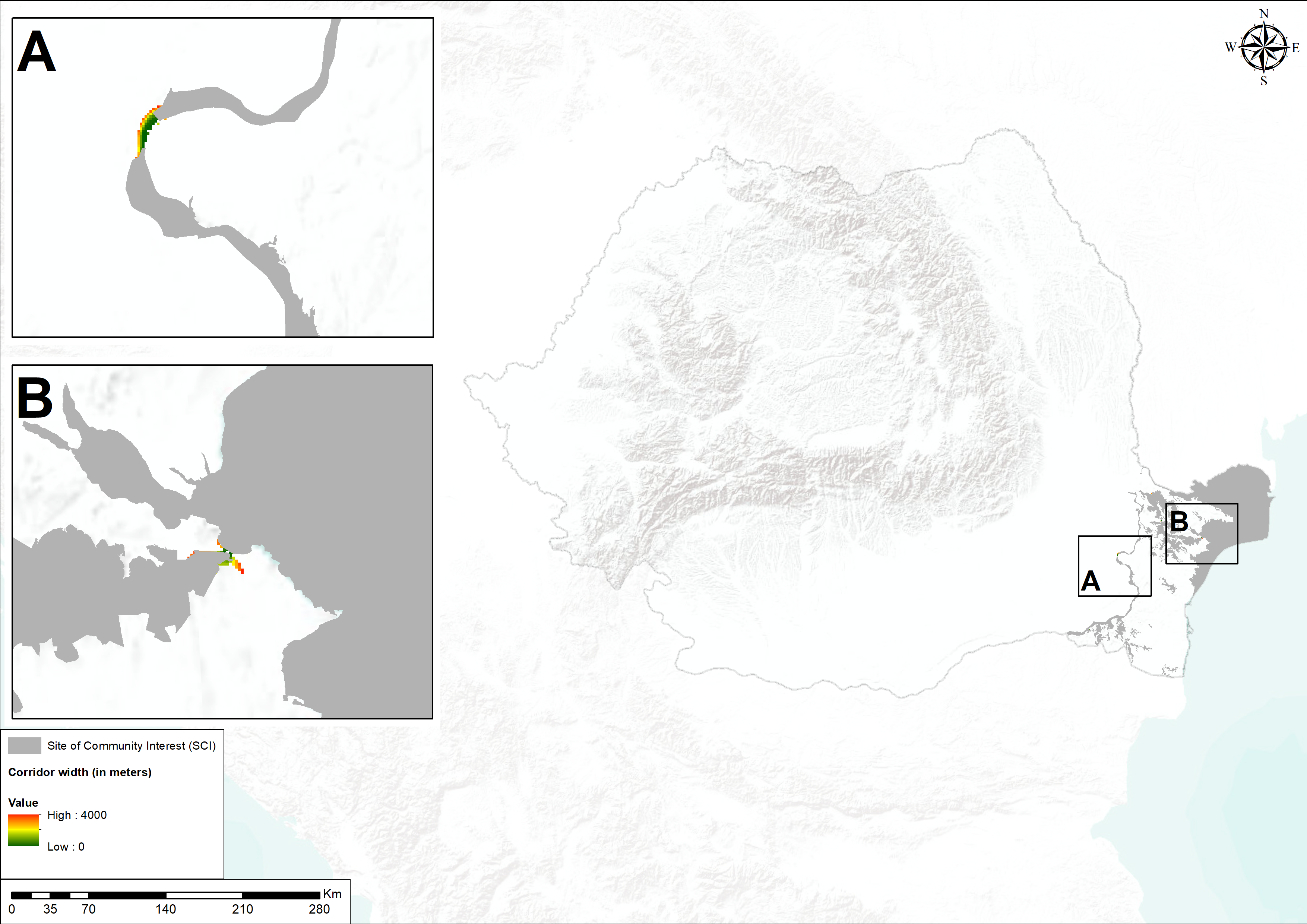

Supplement: Supplementary file 2 — Supplementary information 2. [file 41598_2020_76596_MOESM2_ESM.zip › Supplementary Material S2 Maps/Figure 36 Corridors for Vipera ammodytes montandoni.png]

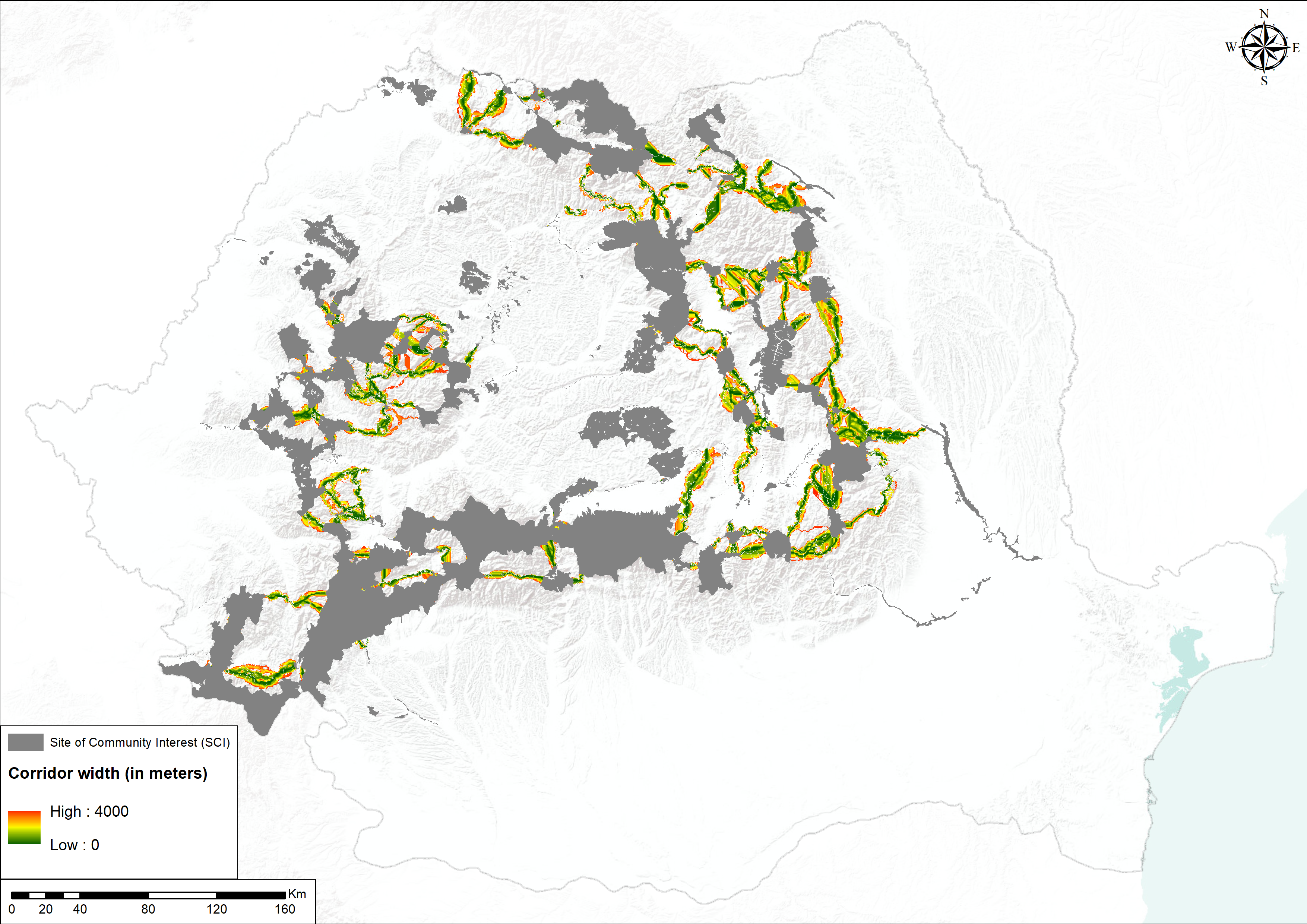

Supplement: Supplementary file 2 — Supplementary information 2. [file 41598_2020_76596_MOESM2_ESM.zip › Supplementary Material S2 Maps/Figure 37 Corridors for Vipera berus.png]

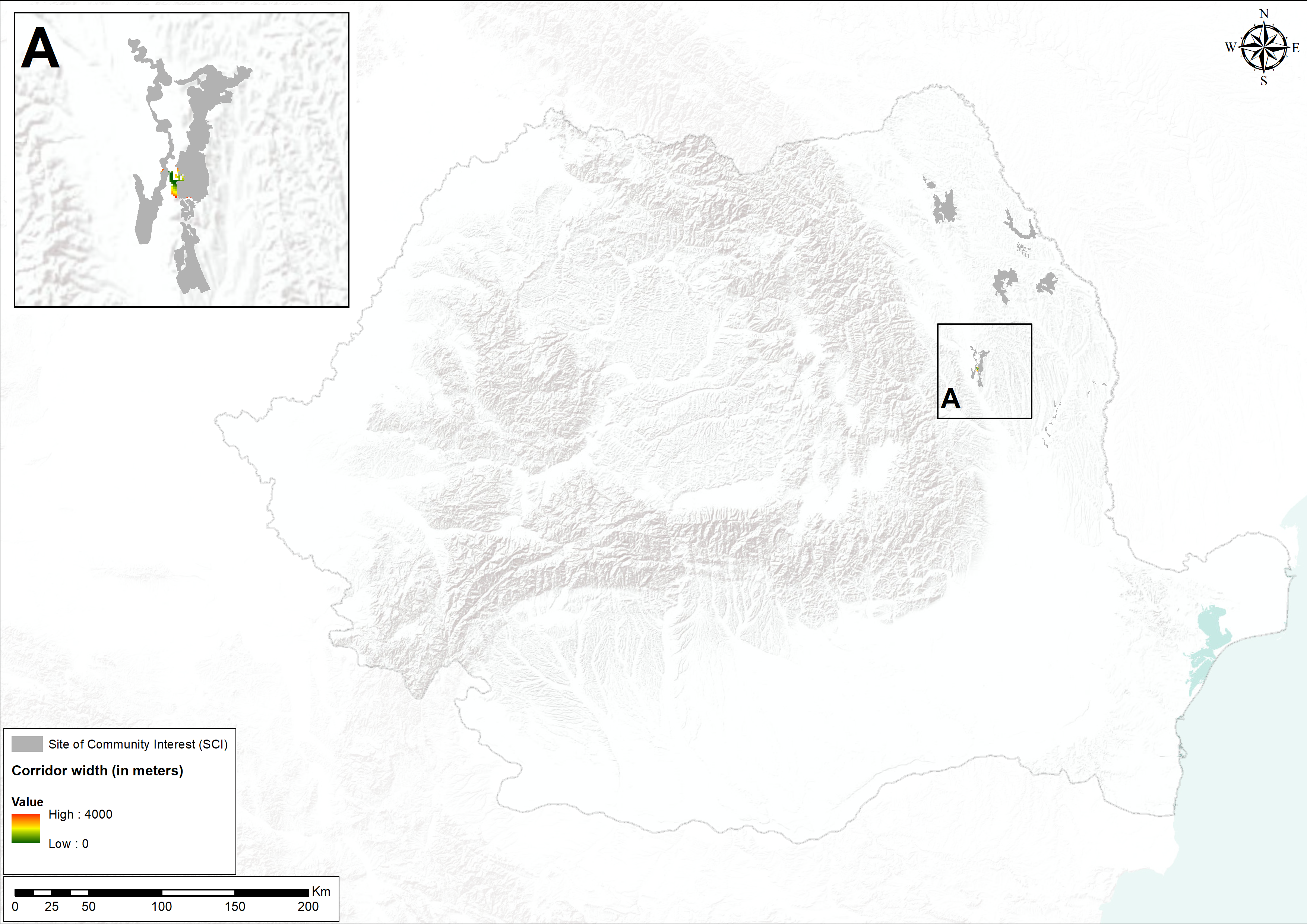

Supplement: Supplementary file 2 — Supplementary information 2. [file 41598_2020_76596_MOESM2_ESM.zip › Supplementary Material S2 Maps/Figure 38 Corridors for Vipera nikolskii.png]

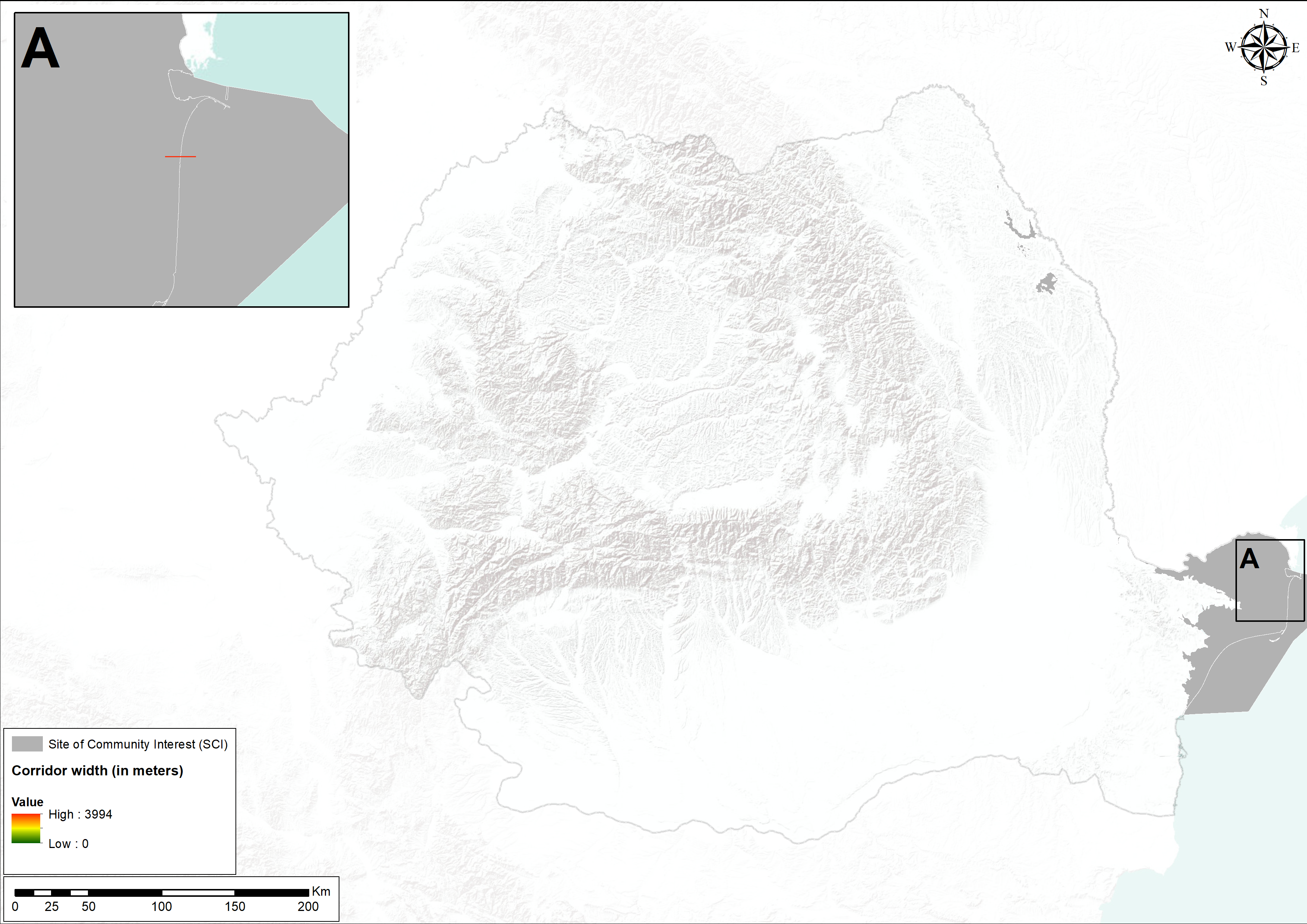

Supplement: Supplementary file 2 — Supplementary information 2. [file 41598_2020_76596_MOESM2_ESM.zip › Supplementary Material S2 Maps/Figure 39 Corridors for Vipera ursinii moldavica.png]

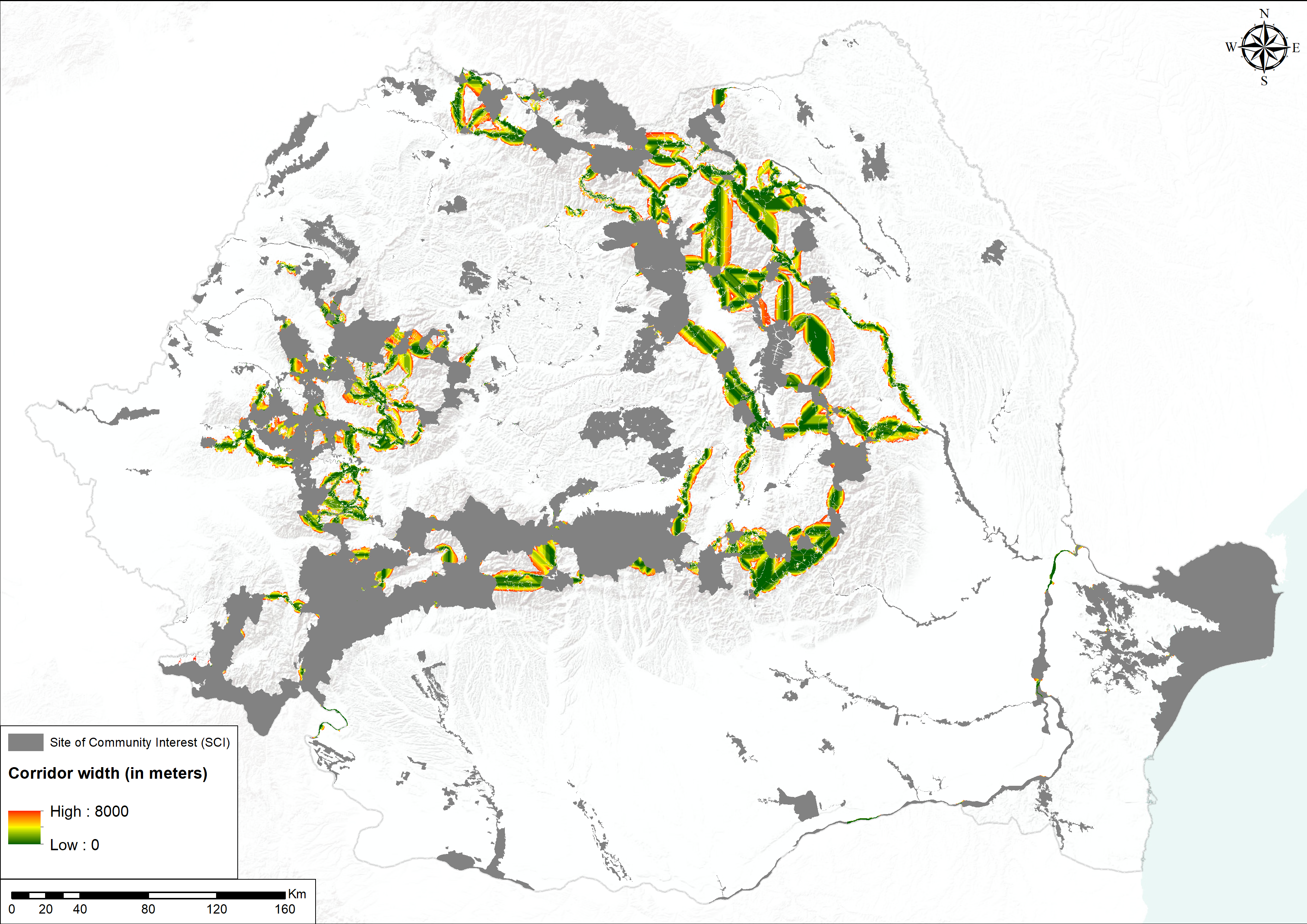

Supplement: Supplementary file 2 — Supplementary information 2. [file 41598_2020_76596_MOESM2_ESM.zip › Supplementary Material S2 Maps/Figure 4 Corridors for Bufo bufo.png]

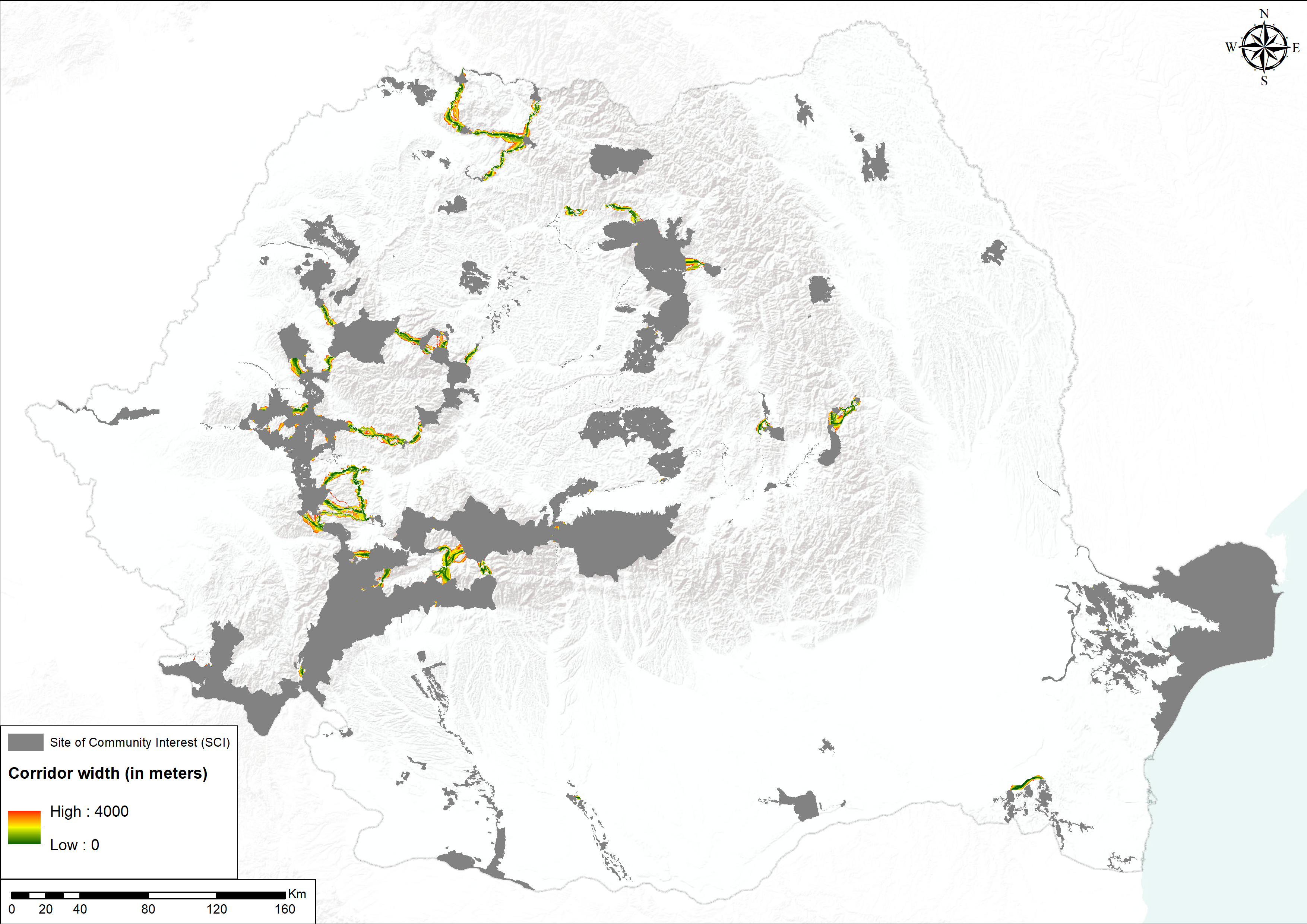

Supplement: Supplementary file 2 — Supplementary information 2. [file 41598_2020_76596_MOESM2_ESM.zip › Supplementary Material S2 Maps/Figure 40 Corridors for Zamenis longissimus.png]

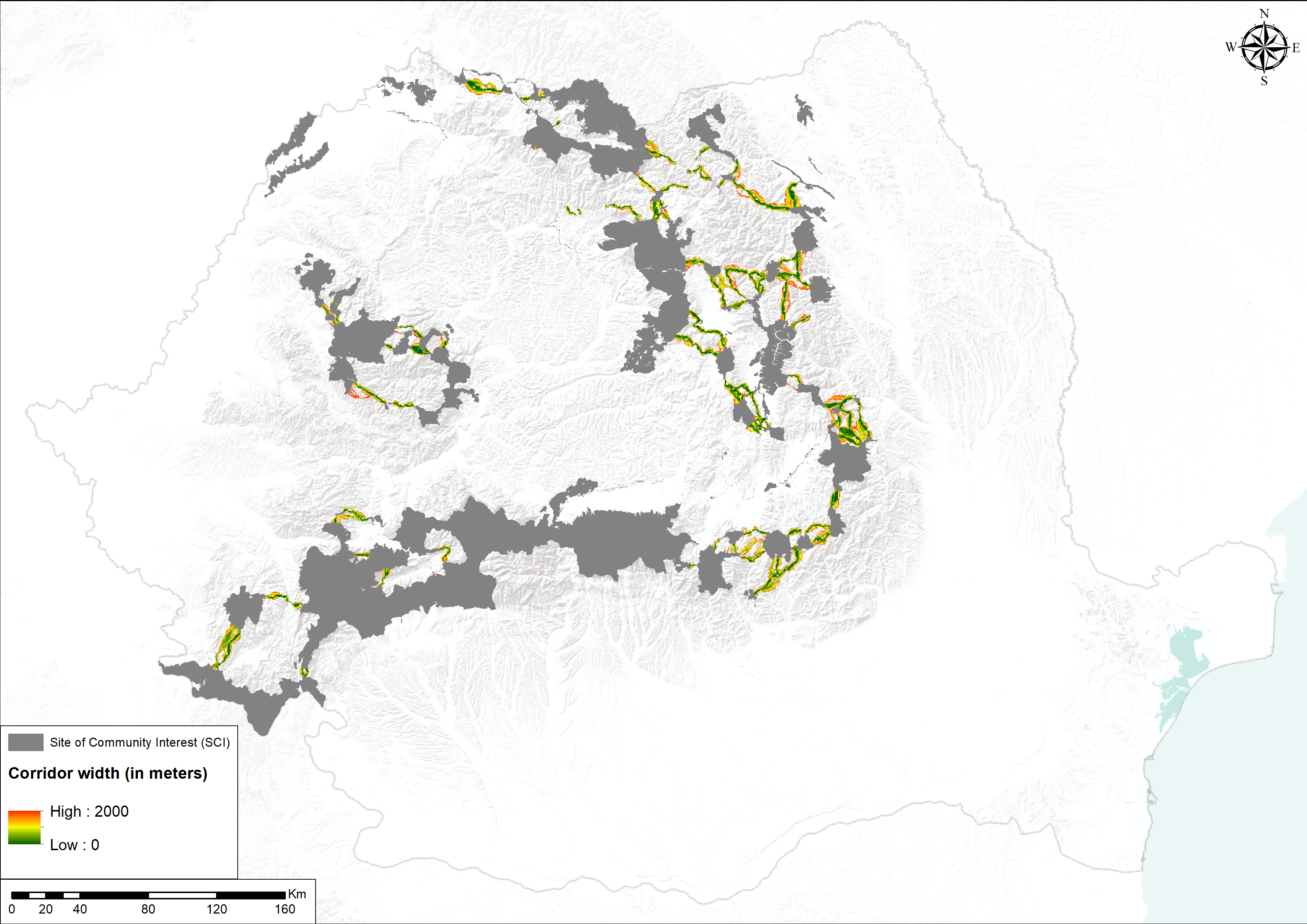

Supplement: Supplementary file 2 — Supplementary information 2. [file 41598_2020_76596_MOESM2_ESM.zip › Supplementary Material S2 Maps/Figure 41 Corridors for Zootoca vivipara.png]

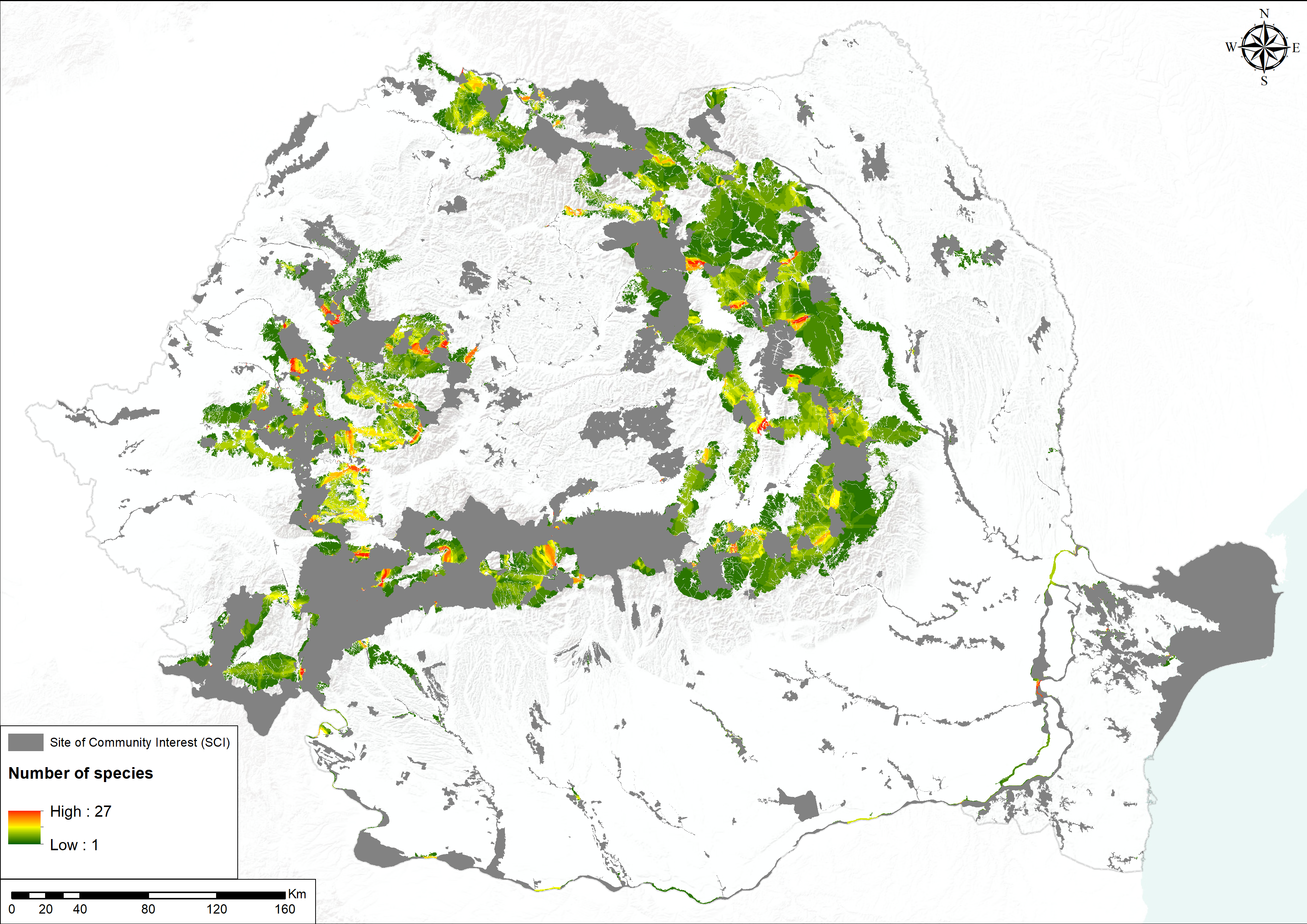

Supplement: Supplementary file 2 — Supplementary information 2. [file 41598_2020_76596_MOESM2_ESM.zip › Supplementary Material S2 Maps/Figure 42 Corridors for all amphibians and reptiles.png]

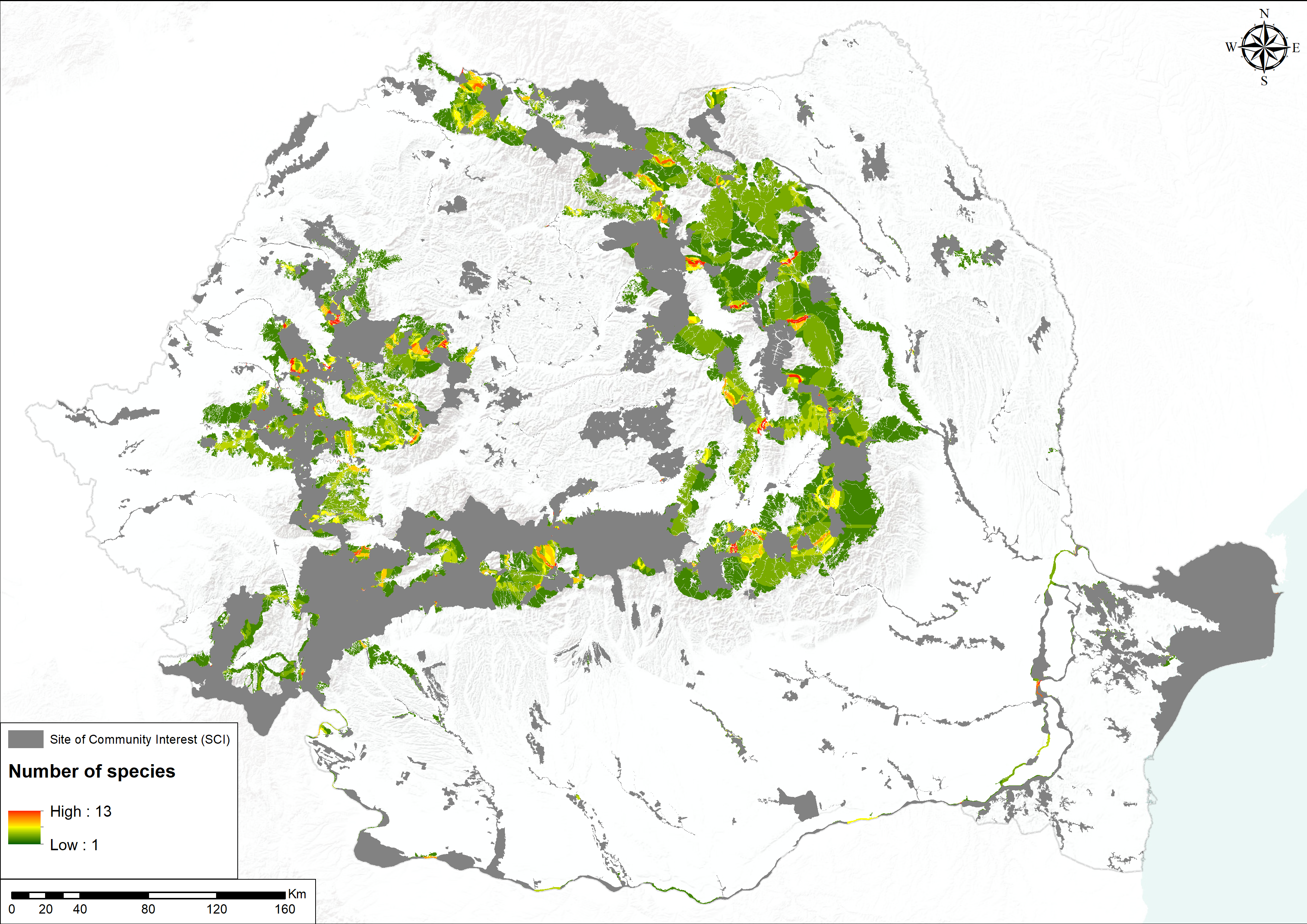

Supplement: Supplementary file 2 — Supplementary information 2. [file 41598_2020_76596_MOESM2_ESM.zip › Supplementary Material S2 Maps/Figure 43 Corridors for amphibians.png]

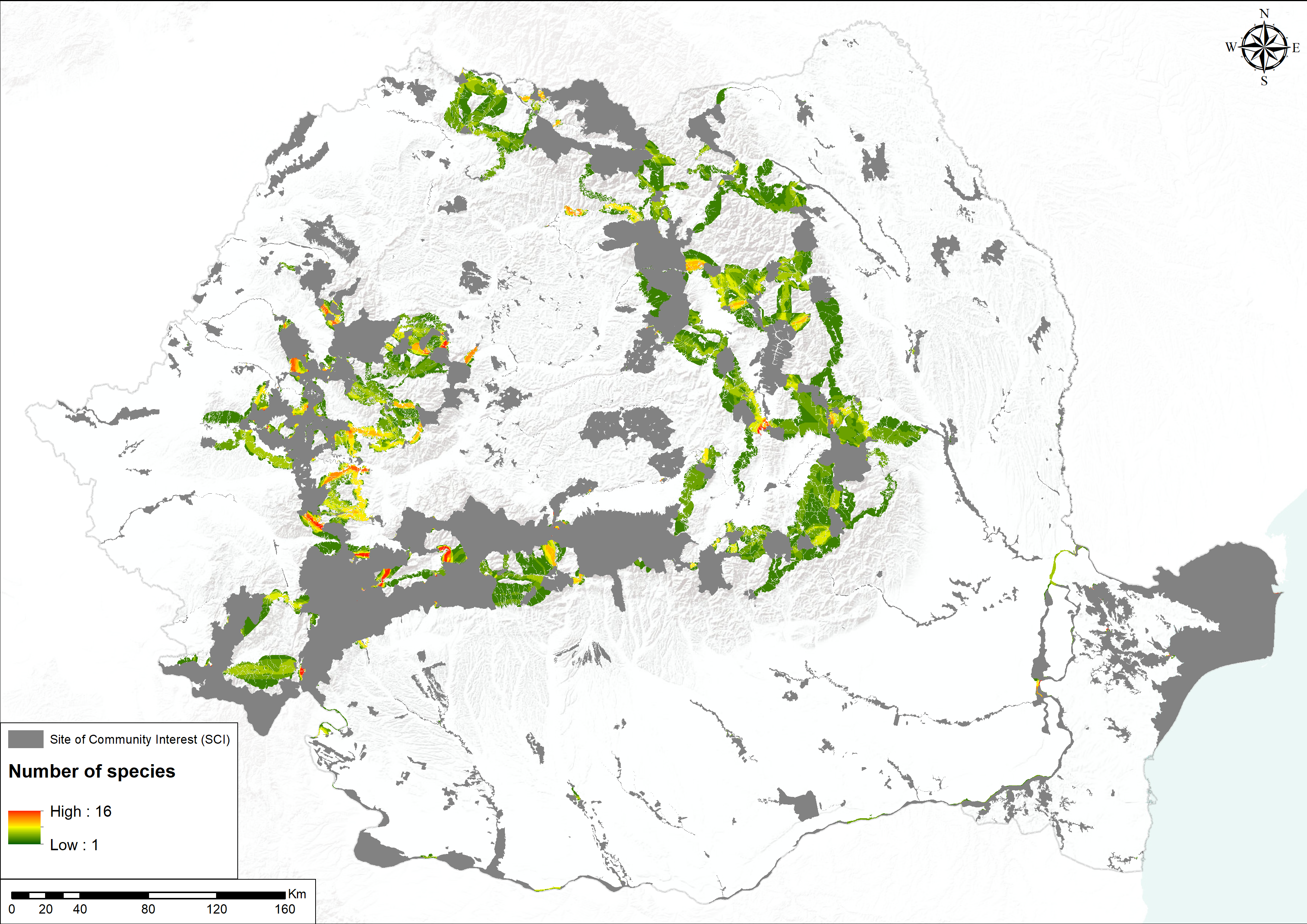

Supplement: Supplementary file 2 — Supplementary information 2. [file 41598_2020_76596_MOESM2_ESM.zip › Supplementary Material S2 Maps/Figure 44 Corridors for reptiles.png]

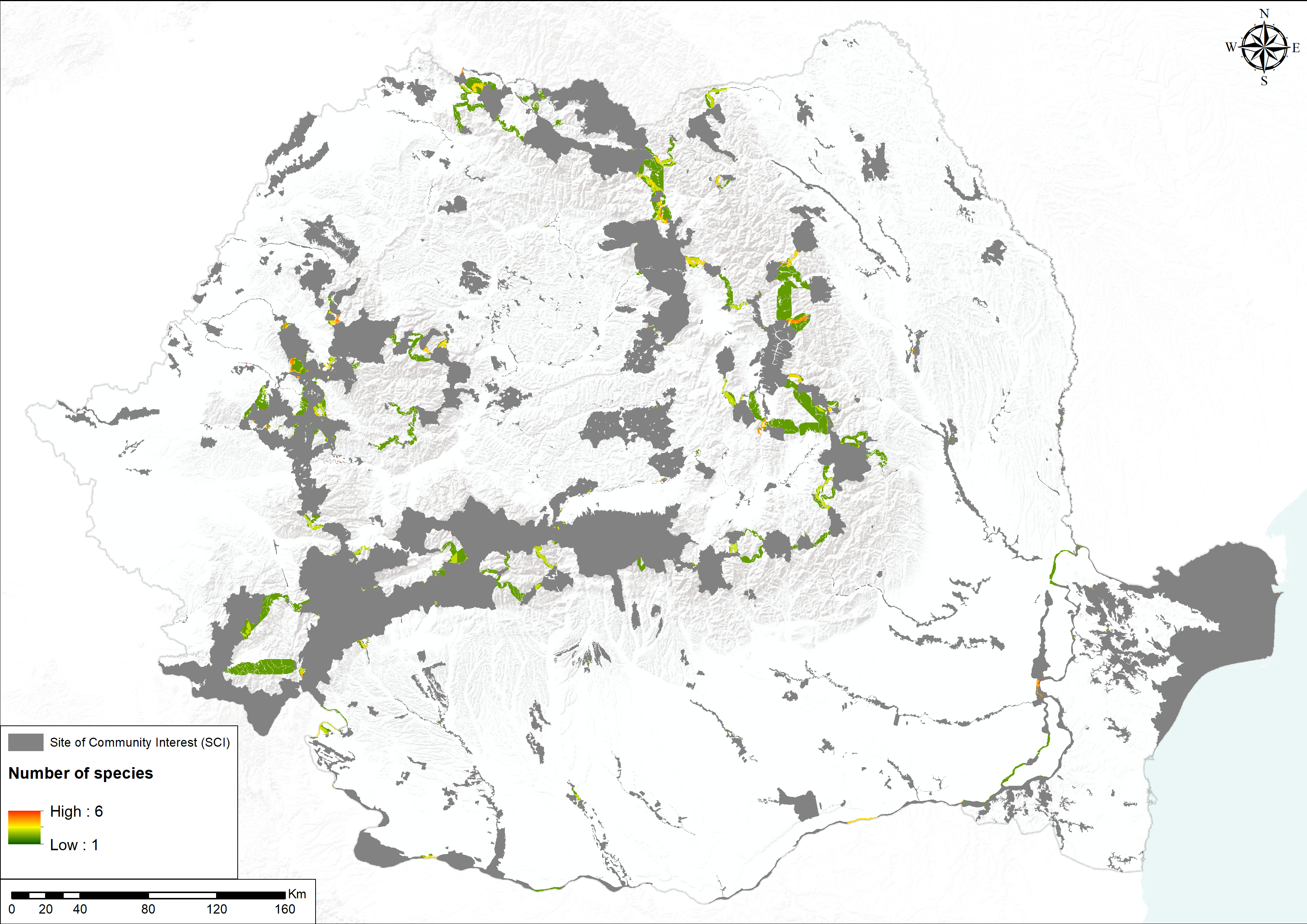

Supplement: Supplementary file 2 — Supplementary information 2. [file 41598_2020_76596_MOESM2_ESM.zip › Supplementary Material S2 Maps/Figure 45 Corridors for Annex II Natura 2000 species.png]

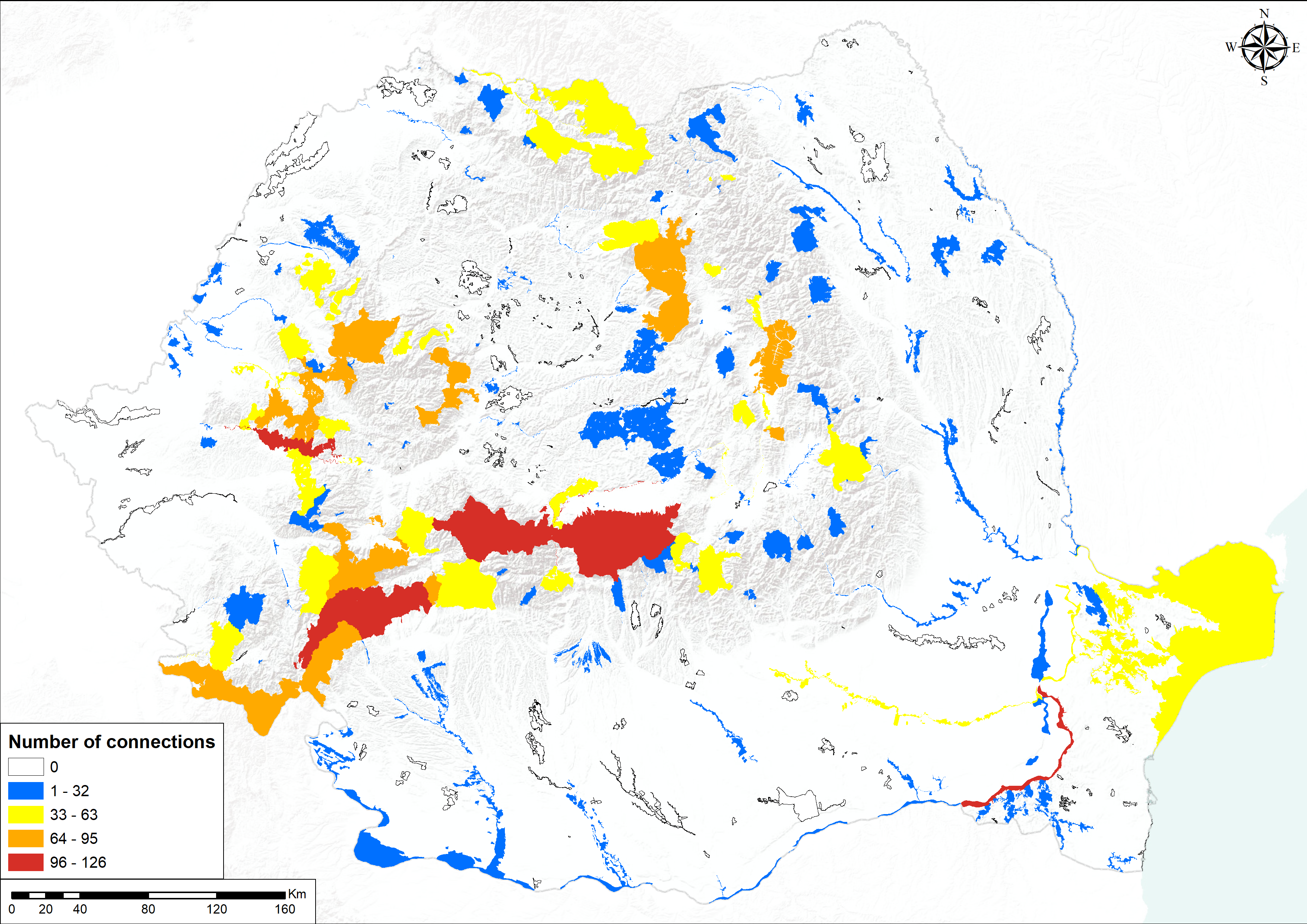

Supplement: Supplementary file 2 — Supplementary information 2. [file 41598_2020_76596_MOESM2_ESM.zip › Supplementary Material S2 Maps/Figure 46 Connectivity of Natura 2000 sites based on LCPs for all herps.png]

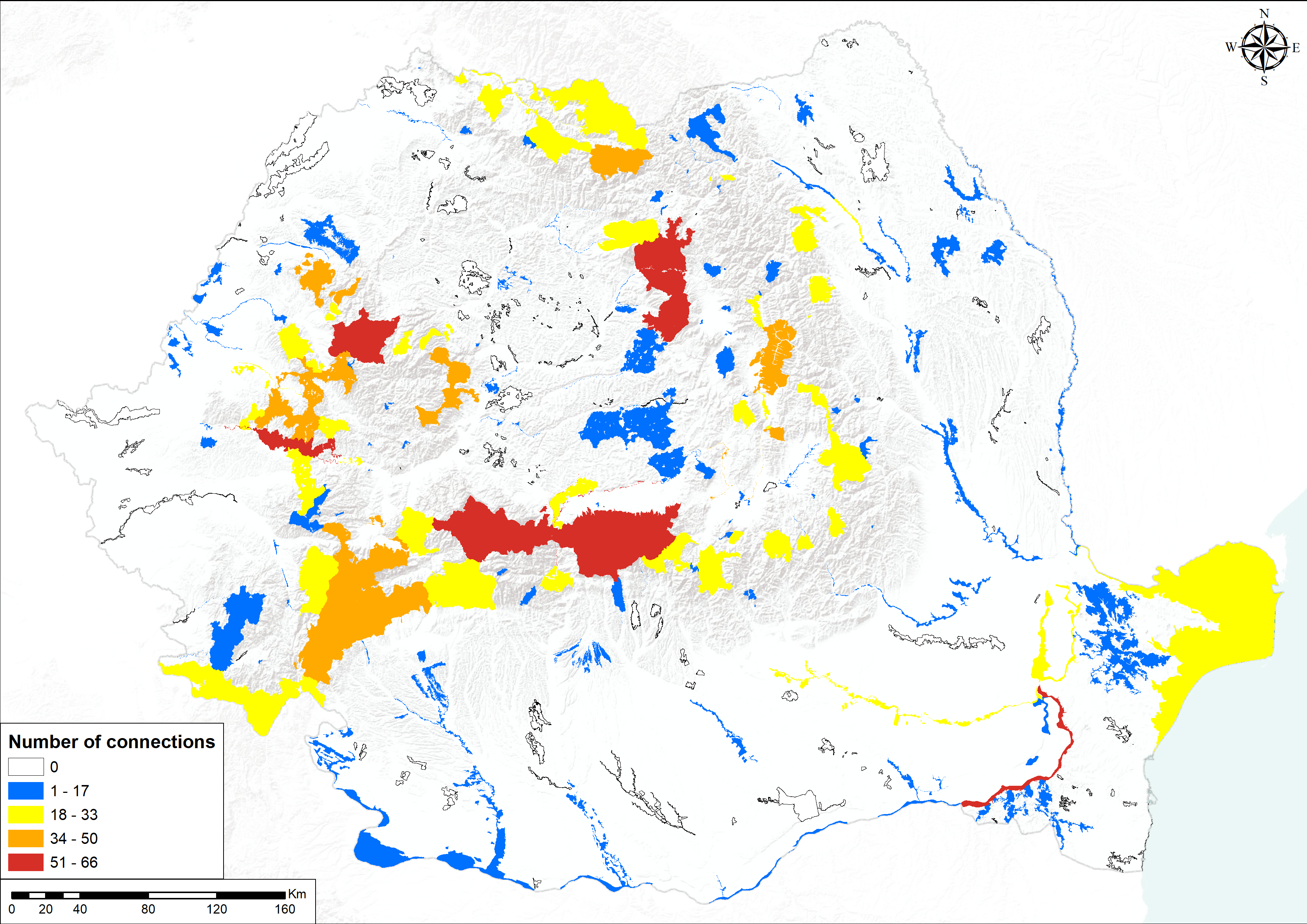

Supplement: Supplementary file 2 — Supplementary information 2. [file 41598_2020_76596_MOESM2_ESM.zip › Supplementary Material S2 Maps/Figure 47 Connectivity of Natura 2000 sites based on LCPs for amphibians.png]

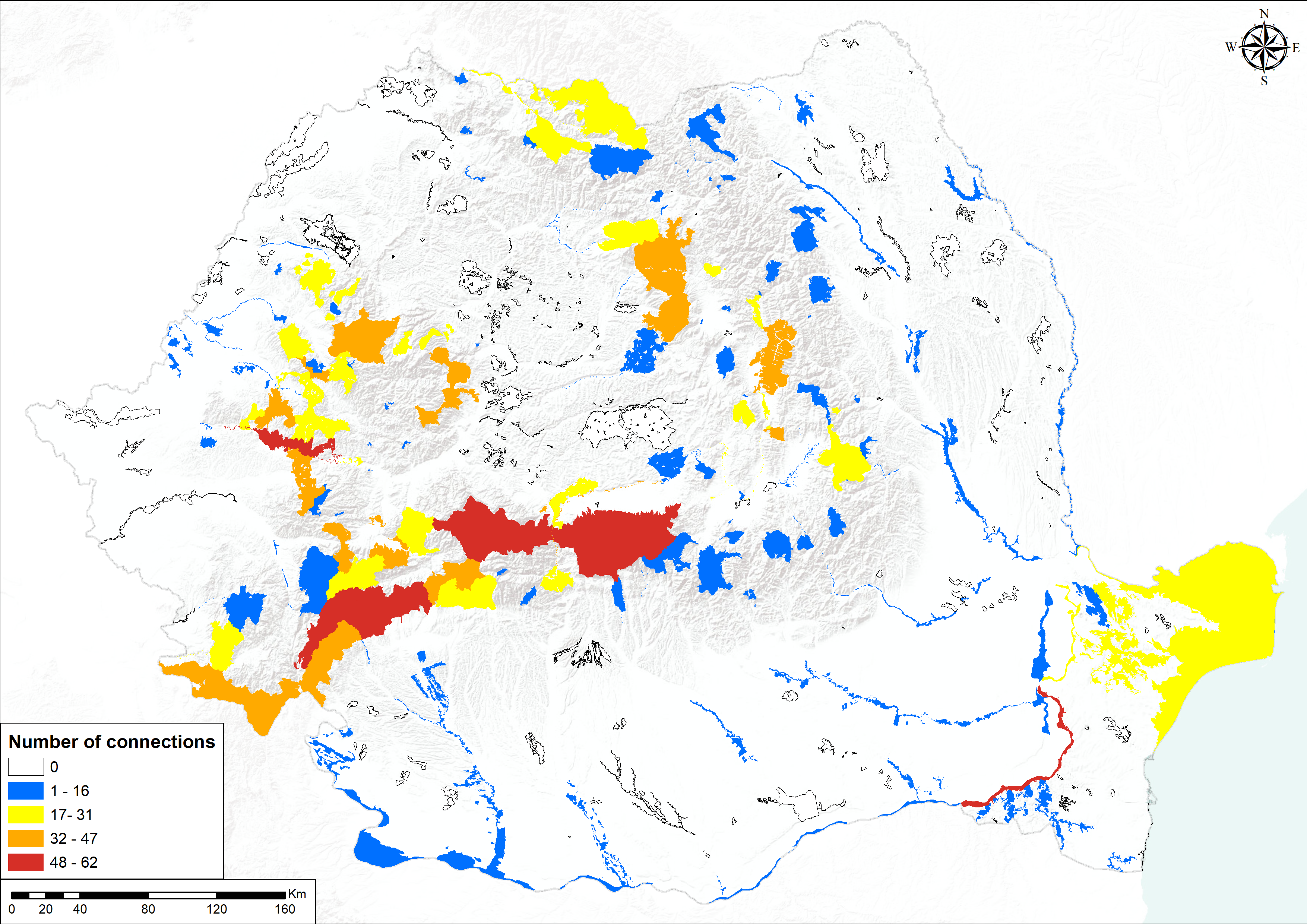

Supplement: Supplementary file 2 — Supplementary information 2. [file 41598_2020_76596_MOESM2_ESM.zip › Supplementary Material S2 Maps/Figure 48 Connectivity of Natura 2000 sites based on LCPs for reptiles.png]

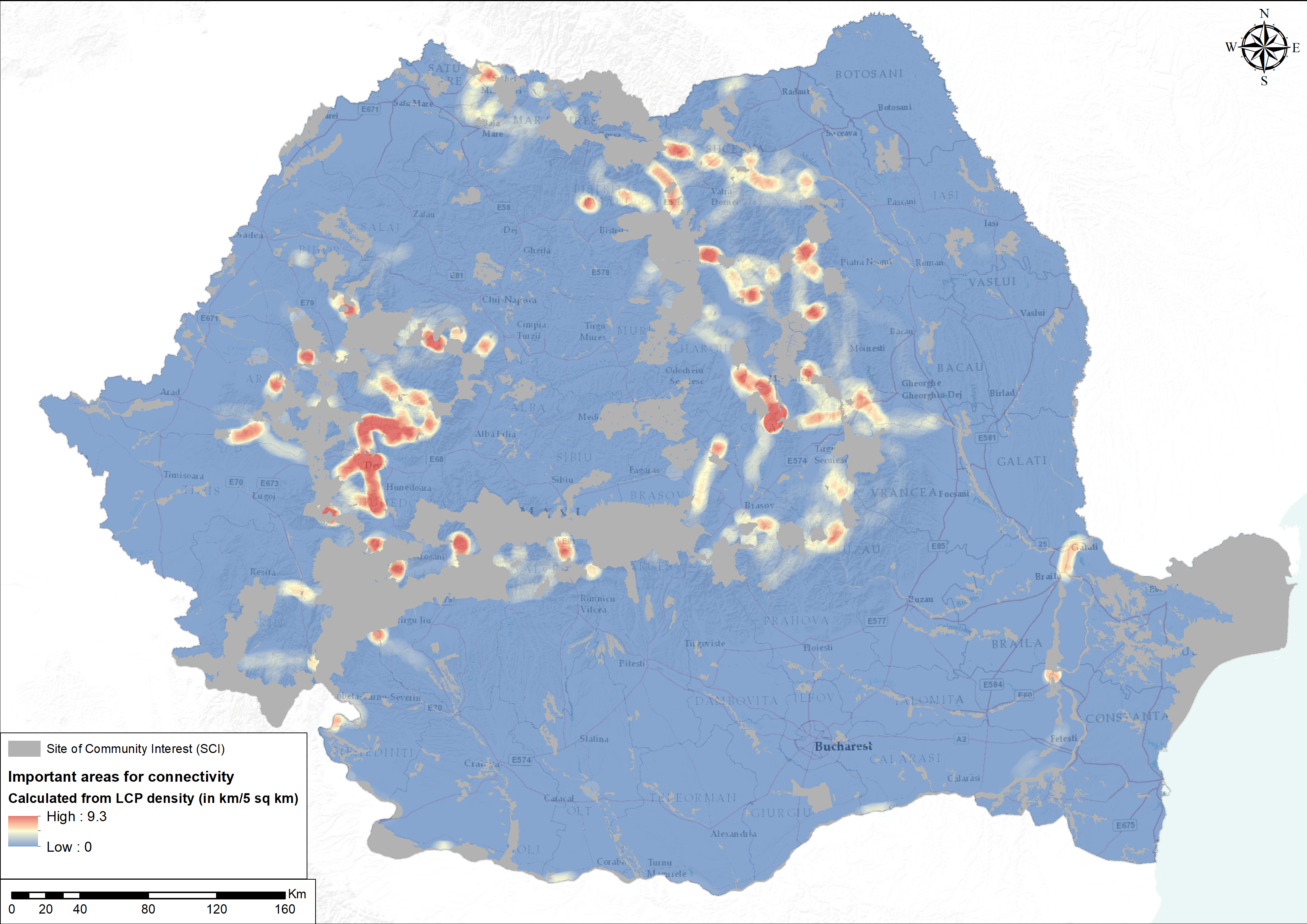

Supplement: Supplementary file 2 — Supplementary information 2. [file 41598_2020_76596_MOESM2_ESM.zip › Supplementary Material S2 Maps/Figure 49 Important Areas for Connectivity.png]

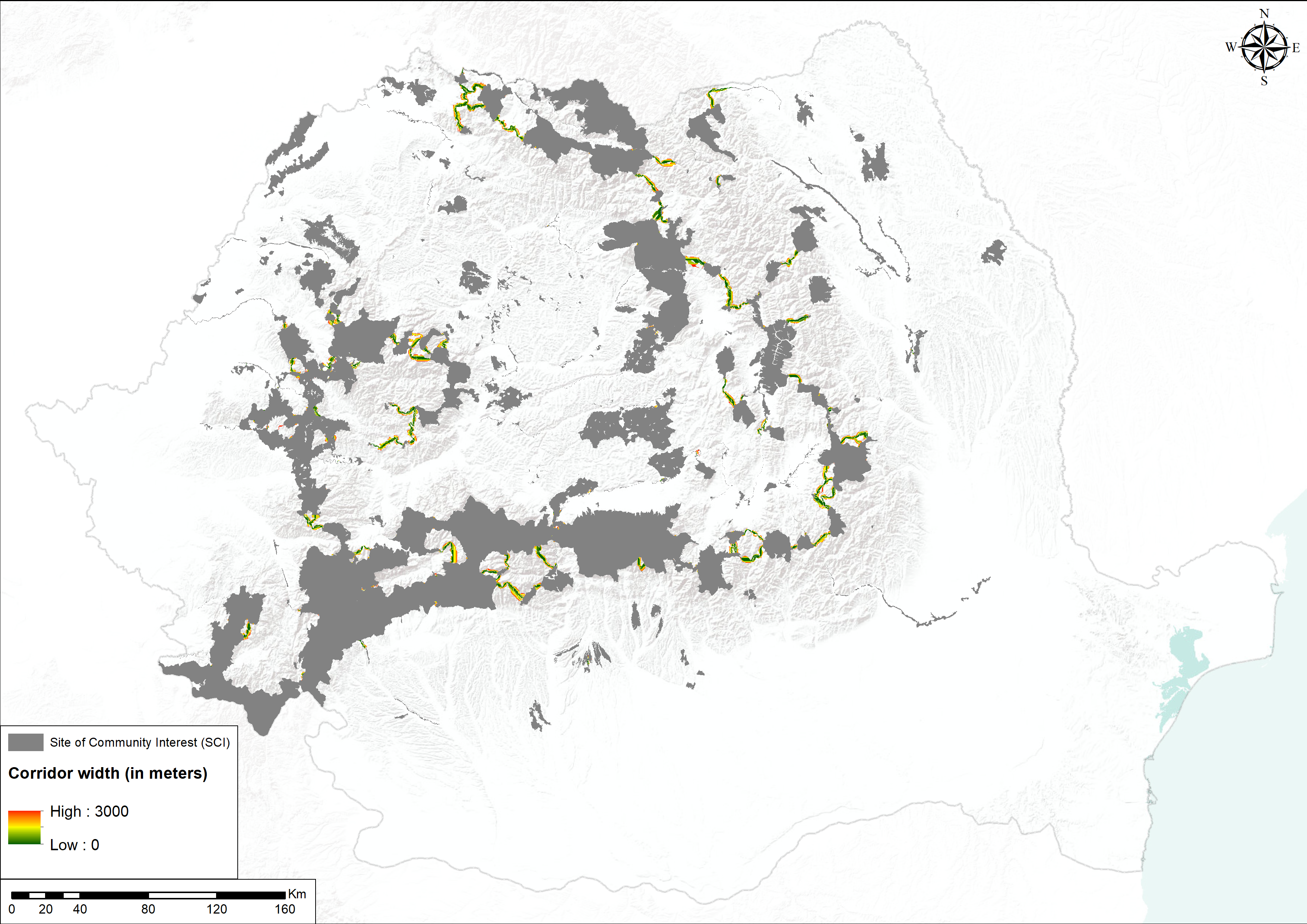

Supplement: Supplementary file 2 — Supplementary information 2. [file 41598_2020_76596_MOESM2_ESM.zip › Supplementary Material S2 Maps/Figure 5 Corridors for Bombina variegata.png]

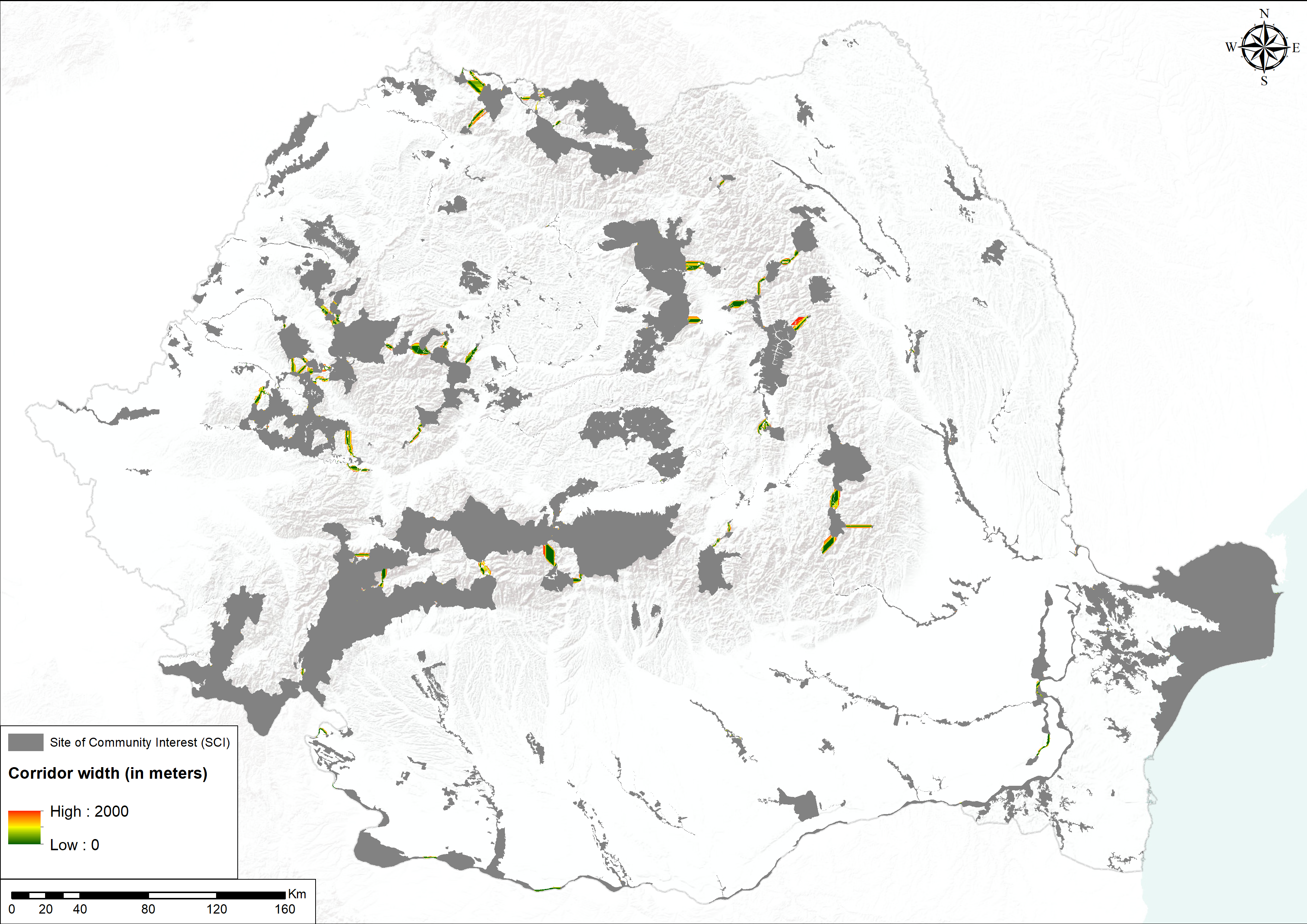

Supplement: Supplementary file 2 — Supplementary information 2. [file 41598_2020_76596_MOESM2_ESM.zip › Supplementary Material S2 Maps/Figure 6 Corridors for Bufo viridis.png]

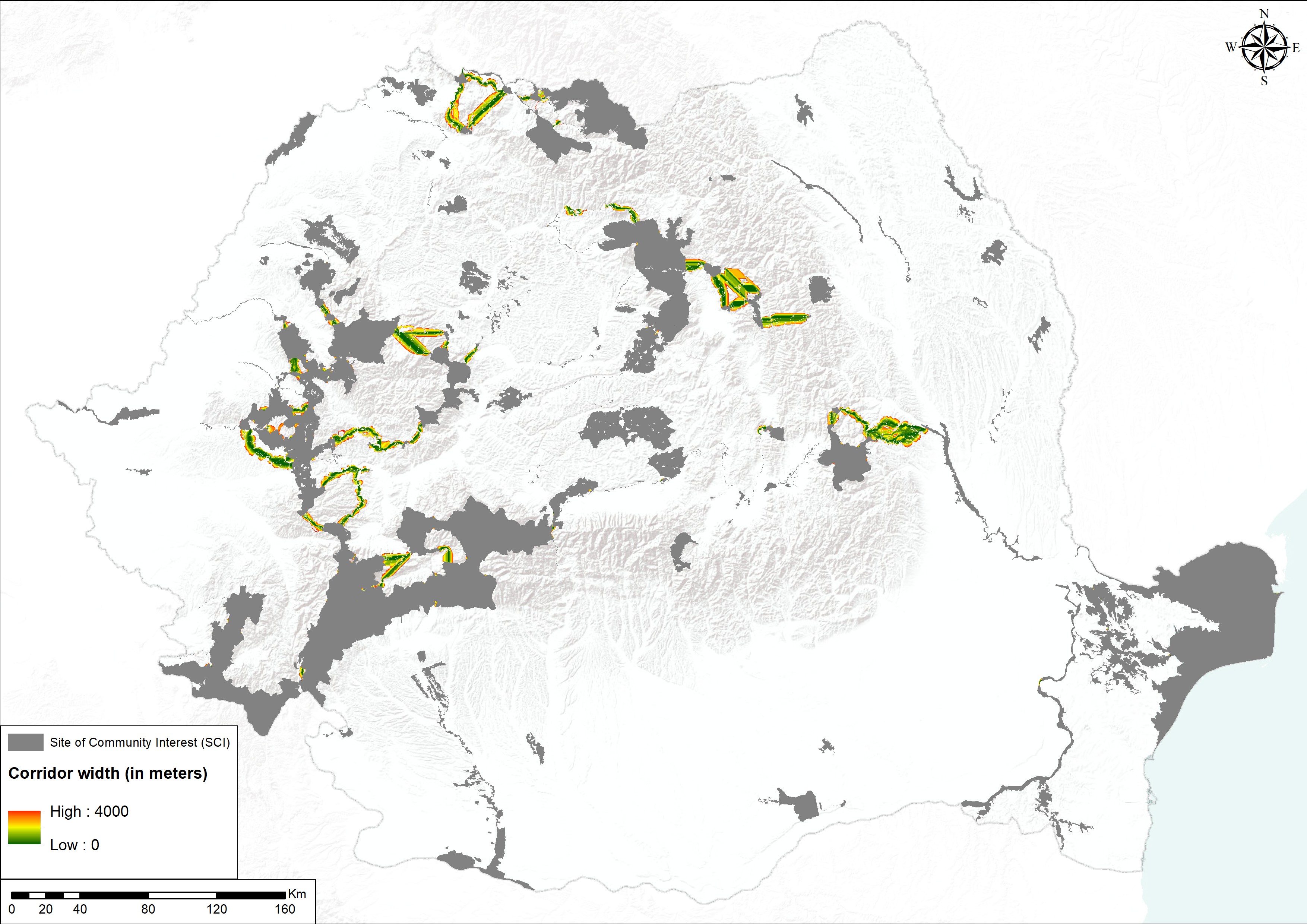

Supplement: Supplementary file 2 — Supplementary information 2. [file 41598_2020_76596_MOESM2_ESM.zip › Supplementary Material S2 Maps/Figure 7 Corridors for Coronella austriaca.png]

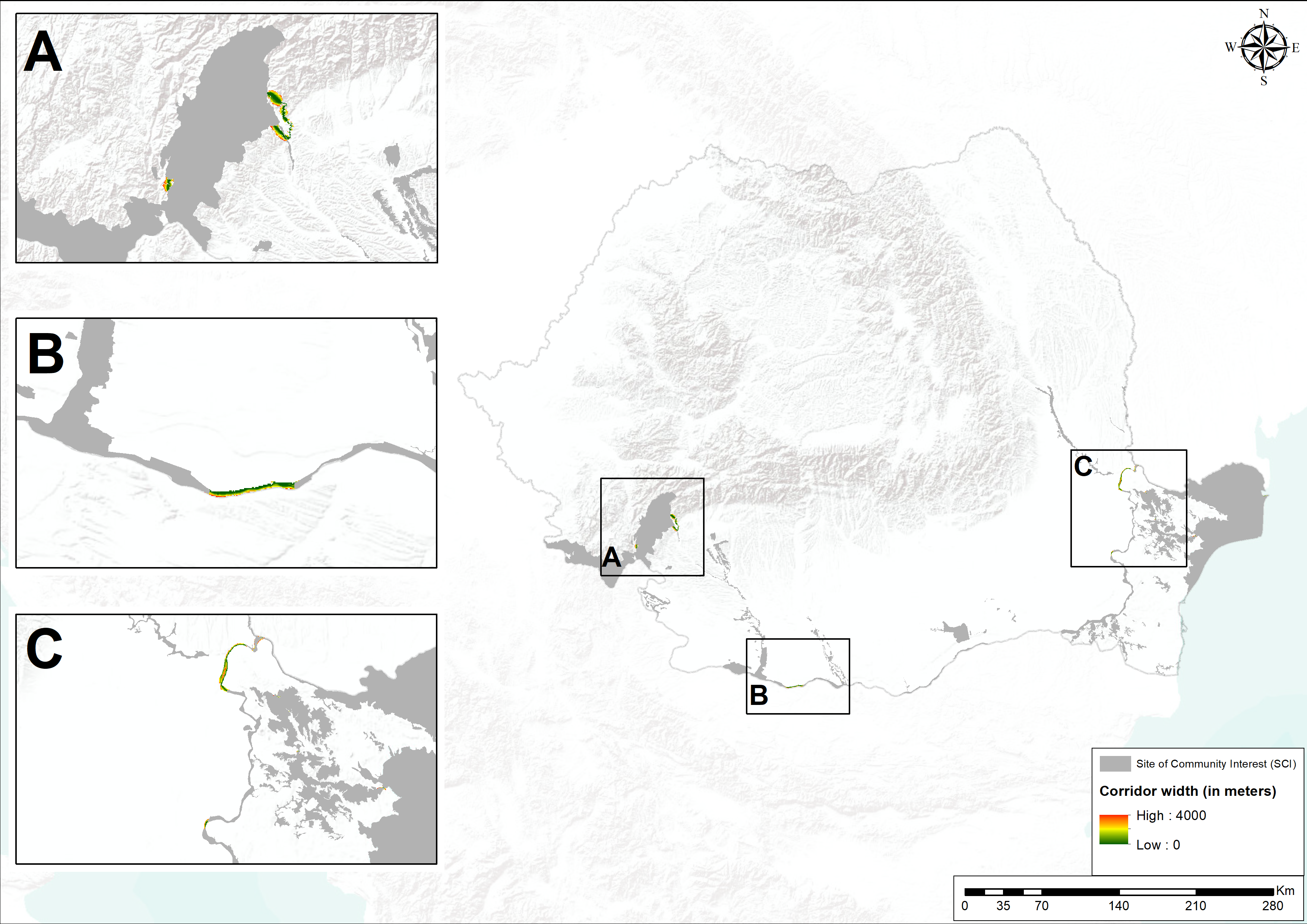

Supplement: Supplementary file 2 — Supplementary information 2. [file 41598_2020_76596_MOESM2_ESM.zip › Supplementary Material S2 Maps/Figure 8 Corridors for Dolichophis caspius.png]

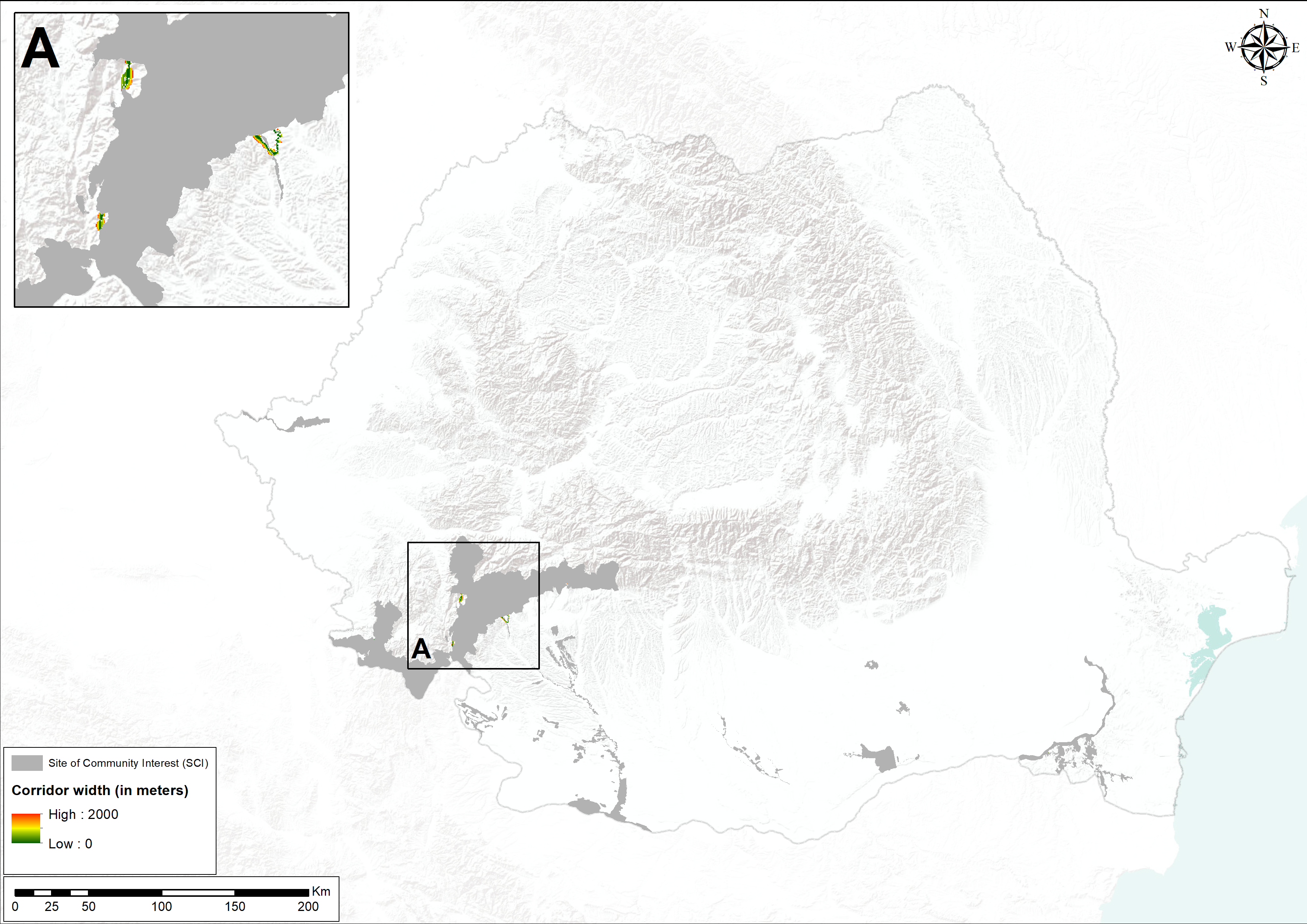

Supplement: Supplementary file 2 — Supplementary information 2. [file 41598_2020_76596_MOESM2_ESM.zip › Supplementary Material S2 Maps/Figure 9 Corridors for Darevskia praticola.png]
