# Supplementary material for: Ecological corridors for the amphibians and reptiles in the Natura 2000 sites of Romania
Source: Sci Rep. 2020 Nov 10;10:19464. doi: 10.1038/s41598-020-76596-z (PMC7655805; doi:10.1038/s41598-020-76596-z)
Supplement: Supplementary file 3 — Supplementary information 3. [file 41598_2020_76596_MOESM3_ESM.docx]

**Additional information regarding the methodology used for creating corridors for the paper titled “Ecological corridors for the amphibians and reptiles in the Natura 2000 sites of Romania”**

Tiberiu C. Sahlean^1*^, Monica Papeș^2^, Alexandru Strugariu^3^, Iulian Gherghel^4,5*^

^1^Department of Patrimony Research, “Grigore Antipa” National Museum of Natural History, 011341 Bucharest, Romania; tiberiu.sahlean@gmail.com. ORCID: 0000-0001-9014-9084;

^2^Department of Ecology and Evolutionary Biology, University of Tennessee, Knoxville, TN, 37996, USA;

^3^Faculty of Biology — Research Department, “Alexandru Ioan Cuza” University of Iasi, 700505 Iasi, Romania;

^4^Department of Biology, Case Western Reserve University, Cleveland, OH 44106, USA. ORCID: 0000-0001-7963-6498;

^5^Faculty of Geography and Geology, “Alexandru Ioan Cuza” University of Iasi, 700505 Iasi, Romania.

*Distribution data*

As mentioned in the main part of this paper, the distribution data used to determine which species inhabit a certain core area were collated from two published reviews regarding the distribution of amphibians and reptiles in Romania^1,2^. The reviews featured distribution maps for each species of herpetofauna, but the original data were not available, hence we used ArcGIS^3^ to manually georeference the distribution maps. The maps presented the centroids of 5 km UTM grids in a national projection (Stereo70) (Figure 1) and all this information was specified in the reviews.

We used a polygon shapefile delineating Romania to create a georeferenced raster based on the distribution map, then we created a new point shapefile in which we stored the occurrence data from the distribution maps. Finally, we used the newly created point shapefile to select the corresponding 5 km UTM cells (Figure 2).

The next step was to add our personal occurrence data, so we selected the 5 km UTM cells that were not part of the original distribution maps and added them to the distribution shapefiles (Figure 2).


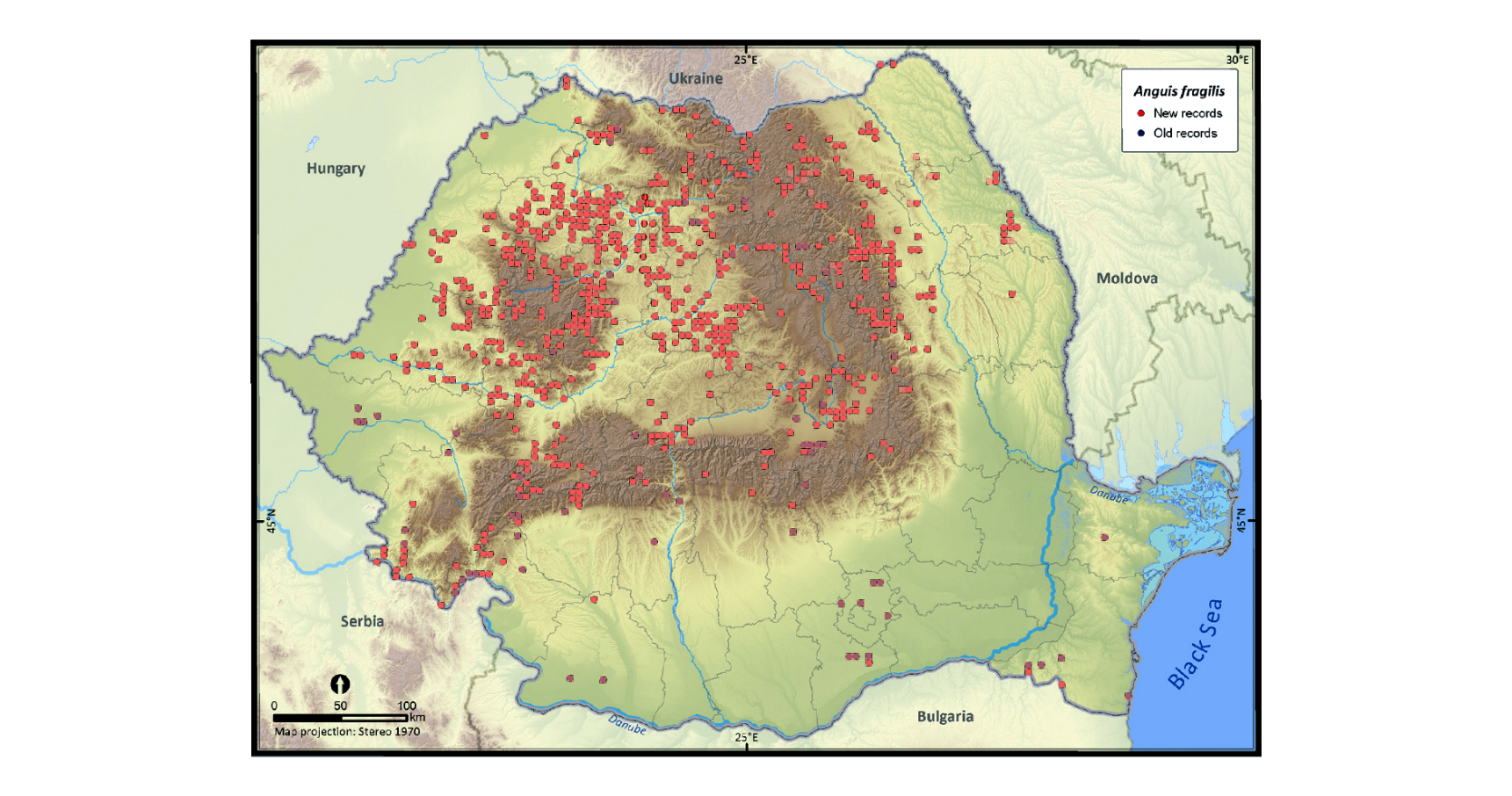


Figure 1 Example of a distribution map from the two peer-reviewed articles^1,2^


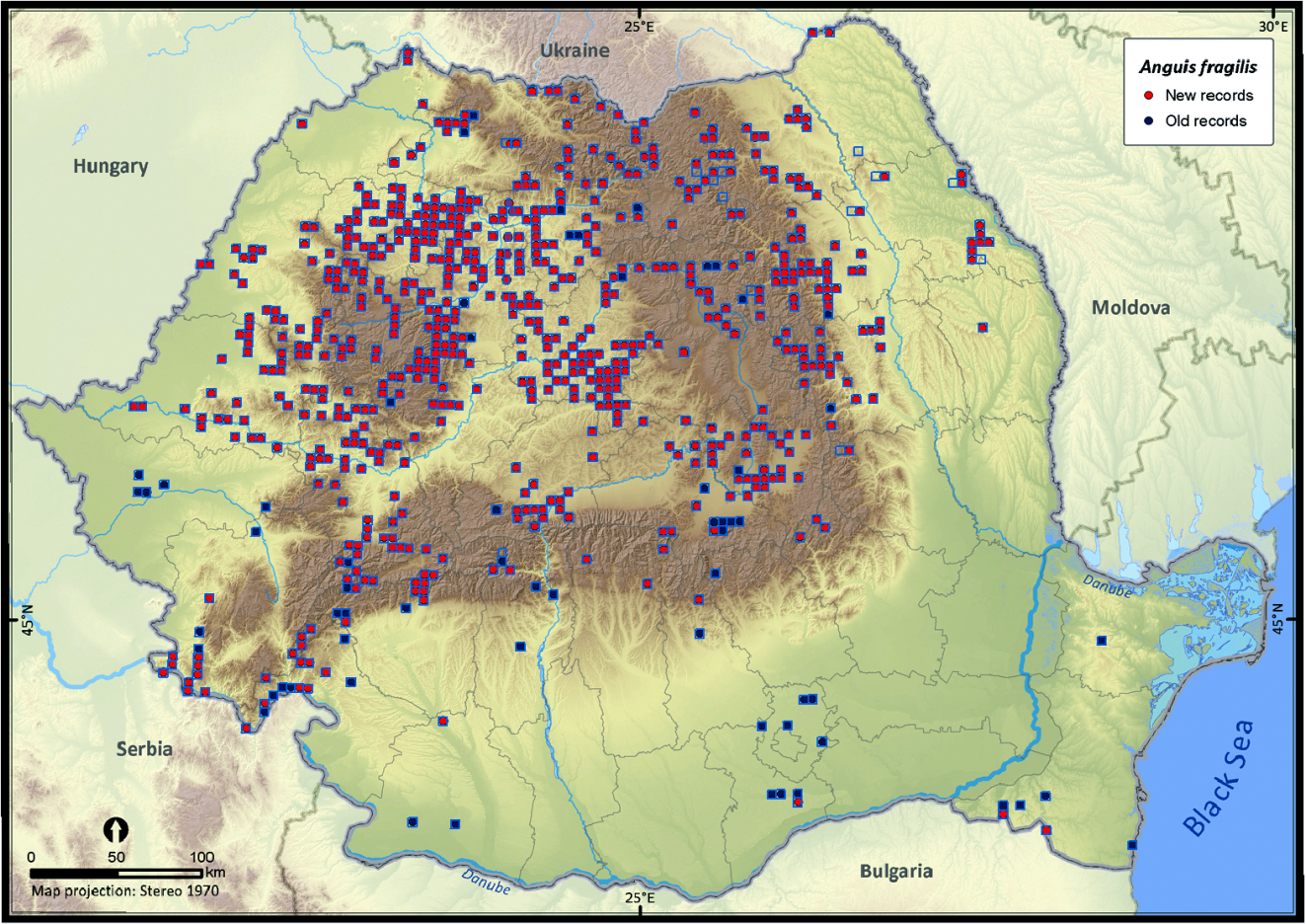


Figure 2 Original distribution data from the peer-reviewed articles^1,2^ with the corresponding 5km UTM cells superimposed (NOTE: the final version of the data is used in this example, which features supplementary cells added by the authors)

*Core areas*

In our experience, the species lists for Natura 2000 sites are rarely up to date as a result of the bureaucratic process involved in updating the species list. For this reason, we chose to manually revise the list of amphibians and reptiles found at each site by intersecting in ArcGIS the species’ distribution grids obtained by georeferencing and updating the maps created by ^1,2^ with the boundaries of the sites from the Natura 2000 network, readily available from the Romanian Ministry of Environment (http://www.mmediu.ro/).

*Corridor model*

The width of the corridor is particularly important because an increased width effectively counters the edge effect, contributes to a greater diversity of habitats, and the habitats will offer sufficient food and shelter from predator, or even reproductive space ^4,5^. However, the width of corridors is a frequent question with no real answer. As a rule of thumb, Harris and Scheck ^6^ suggest that the appropriate width should be measured in kilometers if the corridor is intended to function over a timespan of decades.

*Environmental variables & resistance layers*

One of the most contentious points when designing corridors has been on how to assign scores that accurately reflect the difficulty of a species to navigate certain aspects (quantified as variables) of a habitat. Different methods have been employed, either relying on the experience of the experts, using suitability models with their values reversed or using an evaluation scale to transform variables to a set of common values and applying a weight to each descriptor (see Beier, et al. ^7^ and references therein).

Our study uses scores assigned by the authors, but these scores are only partially based on “expert’s” opinion as the intervals used for scoring (see Supplementary Material S1 - Resistance rasters scoring) were defined through the use of spatial statistics.

After generating the full distribution grids for each species (see *Distribution data* in this supplementary file for the full process) we used ArcGIS and the 5km UTM cells to clip each environmental variable, and therefore we obtained an accurate image of the environmental conditions the species is able to tolerate from the range of environmental conditions available in Romania at the moment.

For example, the elevation in Romania ranges from approximately -15 meters to 2500 meters, but for *Bombina variegata* the range is from -7 meters to ~ 1353 meters. This also highlights why authors also had to make additional decisions regarding the resistance values, since this species does not occur in areas below sea level in Romania and its elevation limit does not exceed 1000 meters a.s.l.

Next, we extracted spatial statistics that reflect a certain set of intervals (C1 – C5, columns C to I in rasters_amphibians and rasters_reptiles and below the species’ names in the sheets clc_amphibians and clc_reptiles). The first interval contains values outside the minimal values from the species distribution range, the second contains values up to the first percentile, the third contains the bulk of the values from the range, the forth interval contains high values from the third percentile to the species’ maximum in its distribution range and the fifth interval contains high values outside those known from the animal’s range. These intervals were assumed to be biologically meaningful, as they reflect conditions outside the known range, conditions towards the limit of their range (below the first percentile and beyond the third percentile) as well as conditions which make up the bulk of the species’ range.

In the case of the landcover raster, statistics could not be calculated because the data are categorical; as such the values under the species’ name represent the number of pixels (with a resolution of 100 meters) within each category, therefore offering a picture of the conditions within the species’ range.


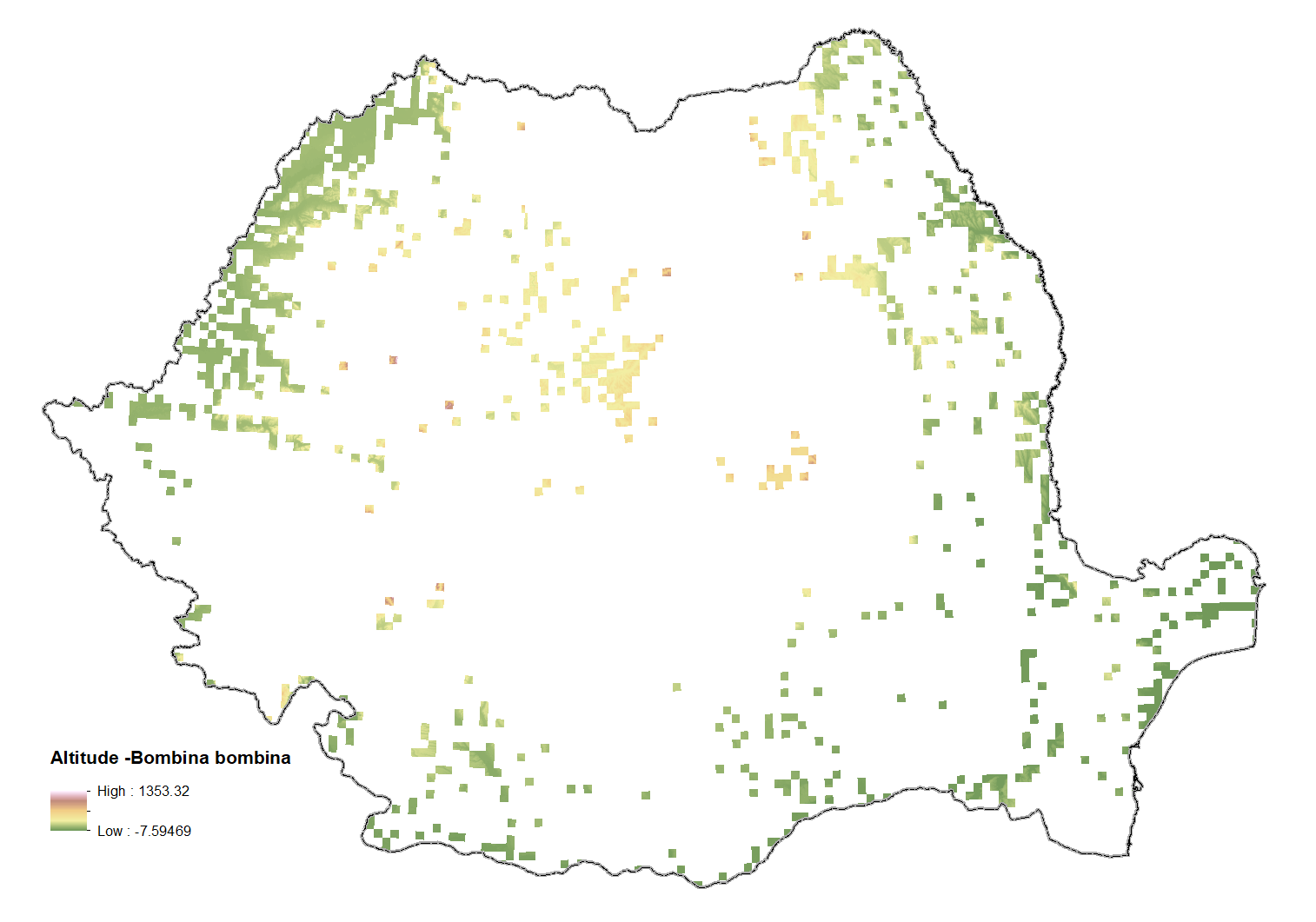


Figure 3 Actual elevation range of *Bombina bombina* based on the distribution grids generated

**Literature cited**

1 Cogălniceanu, D. *et al.* Diversity and distribution of amphibians in Romania. *ZooKeys* **296**, 35 - 57 (2013).

2 Cogălniceanu, D. *et al.* Diversity and distribution of reptiles in Romania. *ZooKeys* **341**, 49-76 (2013).

3 ArcGIS Release 10.4 (Redlands, CA, 2013).

4 Bennett, A. F. *Linkages in the Landscape: The Role of Corridors and Connectivity in Wildlife Conservation*. (IUCN, 2003).

5 Fleury, A. M. & Brown, R. D. A framework for the design of wildlife conservation corridors with specific application to southwestern Ontario. *Landscape and Urban Planning* **37**, 163-186 (1997).

6 Harris, L. D. & Scheck, J. in *Nature Conservation 2: The Role of Corridors* (eds D.A. Saunders & R.J. Hobbs) 189-220 (Surrey Beatty & Sons, 1991).

7 Beier, P., Majka, D. R. & Spencer, W. D. Forks in the Road: Choices in Procedures for Designing Wildland Linkages. *Conservation Biology* **22**, 836-851, doi:DOI: 10.1111/j.1523-1739.2008.00942.x (2008).
